# A Hierarchical Metal–Organic Framework Intensifying ROS Catalytic Activity and Bacterial Entrapment for Engineering Self‐Antimicrobial Mask

**DOI:** 10.1002/advs.202410703

**Published:** 2024-12-16

**Authors:** Wei Huang, Haitao Yuan, Huangsheng Yang, Yujian Shen, Lihong Guo, Ningyi Zhong, Tong Wu, Yong Shen, Guosheng Chen, Siming Huang, Li Niu, Gangfeng Ouyang

**Affiliations:** ^1^ School of Chemical Engineering and Technology Southern Marine Science and Engineering Guangdong Laboratory (Zhuhai) Sun Yat‐sen University Zhuhai 519082 P. R. China; ^2^ Center for Drug Research and Development Guangdong Provincial Key Laboratory of Advanced Drug Delivery System Guangdong Pharmaceutical University Guangzhou 510006 P. R. China; ^3^ School of Chemistry Sun Yat‐sen University Guangzhou 510006 P. R. China; ^4^ Department of Radiology The Third Affiliated Hospital of Southern Medical University Southern Medical University Guangzhou 510630 P. R. China; ^5^ Sun Yat‐sen University School of Chemistry and Guangdong Basic Research Center of Excellence for Functional Molecular Engineering Sun Yat‐sen University Guangzhou 510006 P. R. China; ^6^ Guangzhou Municipal and Guangdong Provincial Key Laboratory of Molecular Target & Clinical Pharmacology the NMPA and State Key Laboratory of Respiratory Disease School of Pharmaceutical Sciences and the Fifth Affiliated Hospital Guangzhou Medical University Guangzhou 511436 P. R. China

**Keywords:** antibacterial material, catalysis, metal–organic framework, nanozyme, reactive oxygen species

## Abstract

Leveraging functional materials to develop advanced personal protective equipment is of significant importance for cutting off the propagation of infectious diseases, yet faces ongoing challenges owing to the unsatisfied antimicrobial efficiency. Herein a hierarchically porous cerium metal–organic framework (Ce‐MOF) boosting the antimicrobial performance by intensifying catalytic reactive oxygen species (ROS) generation and bacterial entrapment simultaneously is reported. This Ce‐MOF presents dendritic surface topography and hierarchical pore channels where the Lewis acid Ce sites are dispersedly anchored. Attributing to this sophisticated nanoarchitecture rendering the catalytic Ce sites highly accessible, it shows a ca. 1800‐fold activity enhancement for the catalytic conversion of atmospheric oxygen to highly toxic ROS compared to traditional CeO_2_. Additionally, the dendritic and negative‐charged surface engineered in this Ce‐MOF substantially enhances the binding affinity toward positive‐charged bacteria, enabling the spatial proximity between the bacteria and the short‐lived ROS and therefore maximizing the utilization of highly toxic ROS to inactivate bacteria. It is demonstrated that this Ce‐MOF‐integrated face mask displays almost 100% antimicrobial efficacy even in insufficient light and dark scenarios. This work provides important insights into the design of antibacterial MOF materials by a pore‐ and surface‐engineering strategy and sheds new light on the development of advanced self‐antimicrobial devices.

## Graphical abstract

Herein, the development of a protective face mask based on a hierarchically porous cerium metal–organic framework intensifying the bacterial entrapment ability and catalytic reactive oxygen species (ROS) generation at ambient condition is presented. It presents almost 100% antimicrobial efficacies for different bacteria even though in insufficient light (e.g. on cloudy and rainy days) and dark scenarios (e.g. at night).

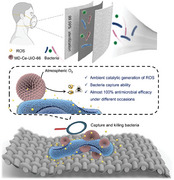

## 1 Introduction

Globalization leads to the transmission of infectious diseases more irregular and faster. This issue was well presented in the microbial infection events such as coronavirus disease 2019 (COVID‐19),^[^ ^1^ ^]^ influenza virus,^[^ ^2^ ^]^ Middle East Respiratory syndrome coronavirus (MERS‐CoV)^[^ ^3^ ^]^ and Ebola virus,^[^ ^4^ ^]^ which cost millions of lives and disturbed global commerce and pose a serious global safety concern. Infectious diseases are mainly transmitted from person to person through direct contact and/or respiration. To dispute the spread of infectious diseases, wearing protective equipment such as face mask, bioprotective clothing, and medical glove, is highly advised by the standard infection prevention and control guidance.^[^ ^5^, ^6^ ^]^ Despite wearing relevant protective equipment is capable of minimizing the biological threats, the infection risk still cannot be totally eradicated, as most of the available protective equipment can only intercept rather than kill the microbes.^[^ ^7^, ^8^ ^]^ Even worse, the accumulated microbes on/in protective equipment are prone to trigger cross‐contamination and aggravate infectious disease transmission.^[^ ^9^, ^10^ ^]^ Hence, it is highly desired to exploit next‐generation antibacterial protective devices capable of physical intercept and chemical neutralization of threatening germs.

A feasible approach to confer protective equipment antimicrobial is incorporating them with biocides.^[^ ^11^, ^12^, ^13^, ^14^, ^15^, ^16^ ^]^ To this point, photocatalytic antimicrobial materials have recently been extensively engineered to meet the basic demands for bioprotective applications, because of their ability to photocatalytically produce reactive oxygen species (ROS), which are capable of serving as highly active oxidants to inactivate harmful microorganisms.^[^ ^11^, ^12^, ^13^, ^15^, ^16^, ^17^, ^18^ ^]^ The photocatalytic equipment, however, are highly relied on the input of photo energy. Although sunlight is sustainably accessible, their immediate biocidal functions suffer from the fast decline or quench under dim light (e.g., on cloudy and rainy days) and/or dark conditions (e.g., at night). This limits the antimicrobial efficacy and practicability of the photocatalytic equipment in diverse occasions.

In nature, some oxidase enables the catalytic generation of ROS using well‐sourced oxygen as the electron acceptor in ambient condition.^[^ ^19^, ^20^, ^21^ ^]^ Limited by the structural vulnerability and expensive cost of natural oxidase,^[^ ^22^ ^]^ the stable and cost‐effective Ce nanoparticle, e.g. ceria (CeO_2_), has garnered great interest in mimicking the oxidase‐like catalytic reaction, attributing to its strong Lewis acidity and facile electron cycle between Ce^3+^ and Ce^4+^.^[^ ^23^, ^24^ ^]^ This offers new opportunities to design the next‐generation self‐antimicrobial protective equipment in an energy input‐ and chemical‐addition‐free principle. However, the ROS generation efficiency of Ce nanomaterials, to date, is far from satisfactory, because of their dense structure that significantly reduces the accessibility of active Ce sites. At the same time, the lifespan of catalytic ROS is extremely short with an effective radius of action less than 200 nm,^[^ ^19^, ^25^, ^26^ ^]^ complicating the utilizing of ROS for targeting and inactivating bacteria.

**Scheme 1:**
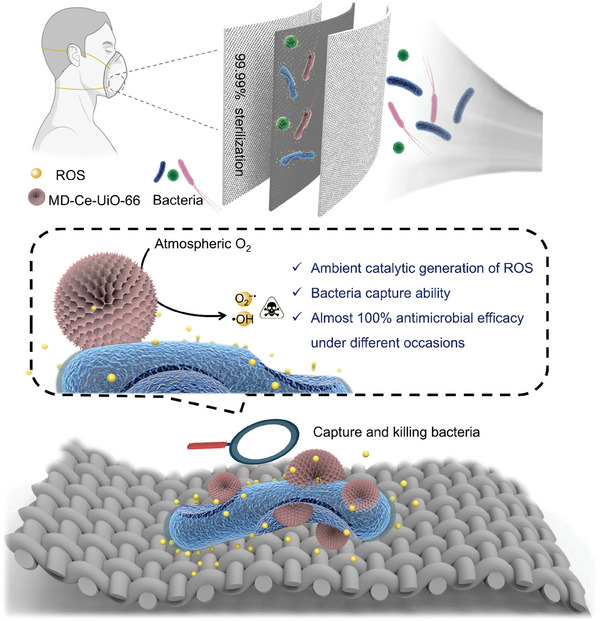
Schematic illustration of the hierarchically porous Ce‐MOF intensifying catalytic ROS generation and bacterial entrapment simultaneously for engineering antimicrobial mask.

**Figure 1:**
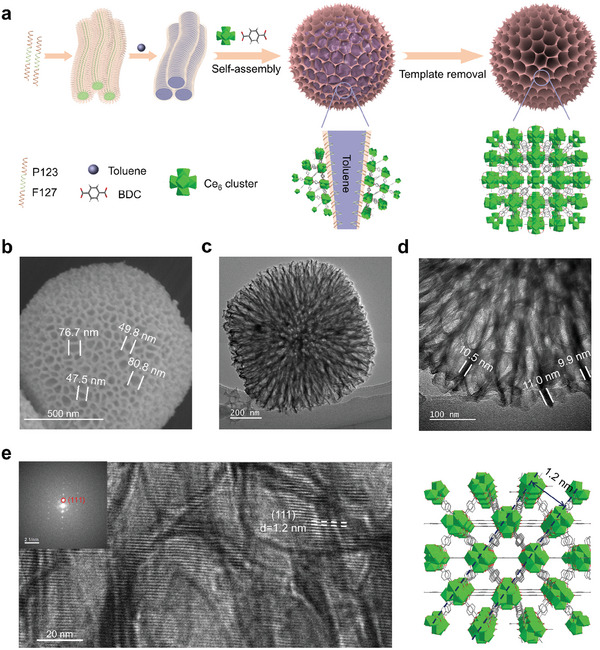
a) Schematic representation of the polymer microemulsion template‐mediated coordination assembly for synthesizing MD‐Ce‐UiO‐66. The SEM b) and TEM images c,d) of MD‐Ce‐UiO‐66 nanoagent. e) Left: The Cryo‐EM image showing the (111) lattice planes throughout the MD‐Ce‐UiO‐66 nanoagent. Right: The crystallographic model of UiO‐66 highlights the (111) lattice plane.

Herein, we present the development of a protective face mask based on a hierarchically porous cerium metal–organic framework (Ce‐MOF), which can boost self‐antimicrobial activity by virtue of intensifying ROS catalytic generation and bacterial entrapment simultaneously (**Scheme** **1**). The hierarchically porous Ce‐MOF is synthesized by a polymer microemulsion template‐directed coordination assembly method (**Figure** **1**a). In this method, the amphipathic block copolymers of Pluronic®P‐123 (PEO_20_PPO_70_PEO_20_, termed P123) and Pluronic®F‐127 (PEO_106_PPO_70_PEO_106_, termed F127) are aggregated and then form linear micelles in aqueous solution. The addition of hydrophobic toluene transforms these linear micelles into columnar microemulsion templates, which co‐stabilized by P123 and F127. Finally, the abundant PEO segments on the columnar microemulsion surface form crown‐ether‐type complexes with Ce^4+^, facilitating the in‐place growth of Ce‐UiO‐66 around the columnar microemulsion templates.^[^ ^27^, ^28^ ^]^ After removing the columnar microemulsion templates, the Ce‐MOF featuring dendritic surface topography and hierarchical pore channels is formed. This hierarchically porous Ce‐MOF renders the catalytic Ce sites highly accessible, facilitating the catalytic conversion of atmospheric oxygen to highly toxic ROS, which is ca. 1800‐fold higher than traditional CeO_2_. Importantly, the dendritic surface shows a much higher binding affinity toward bacteria, significantly shortening the migration distance of short‐life ROS to bacteria and thus maximally harnessing the ROS to inactivate bacteria. This Ce‐MOF is functionally and structurally stable, evidenced by the fact that 70% activity of ROS catalytic generation is preserved after storing at room temperature up to 300 days. The feasibility of utilizing the Ce‐MOF for fabricating antimicrobial protective mask is also demonstrated, presenting almost 100% antimicrobial efficacies for different bacteria even under insufficient light (e.g., on cloudy and rainy days) and dark scenarios (e.g., at night).

## 2 Results and Discussion

### 2.1 Synthesis of Ce‐MOF With Hierarchically Porous Channels and Dendritic Surface

The nanoarchitecture of a material dictates both its catalytic activity and interface chemistry, impacting the subsequent antimicrobial performance.^[^ ^29^, ^30^, ^31^ ^]^ With this in mind, we herein engineered a Ce‐MOF with hierarchically porous channels and dendritic surface, aiming at maximally harnessing the catalytic ROS to inactivate bacteria. The synthesis principle was based on a microemulsion polymer‐templated coordination assembly between cerium (IV) ammonium nitrate and 1,4‐benzenedicarboxylic acid (BDC)^[^ ^27^, ^28^ ^]^ (Figure 1a). After removing the microemulsion template, the Ce‐MOF with mesopore‐macropore channels and dendritic surface were obtained, named MD‐Ce‐UiO‐66 (M and D mean mesopore‐macropore channels and dendritic shape surface, respectively). Powder X‐ray diffraction (PXRD) revealed the high crystallinity of this MD‐Ce‐UiO‐66 nanoagent, and the Bragg diffraction pattern was consistent well with standard UiO‐66 MOF (Figure S1, Supporting Information), the structure of which is formed by the coordination linkage of 12‐connected Ce_6_ cluster and 1,4‐benzenedicarboxylic acid in a ***fcu*** topology.^[^ ^32^ ^]^ In addition, the elemental analysis by the inductively coupled plasma mass spectrometry (ICP‐MS) revealed the Ce ratio of 39.33 wt% in MD‐Ce‐UiO‐66. This was in good agreement with the theoretical value of 41.51%, estimated based on the chemical formula of Ce_6_O_4_(OH)_4_(BDC)_6_ of UiO‐66 (Figure S2 and Table S1, Supporting Information). The slightly decreased Ce content was plausible to be caused by the defective formation in 12‐connected ***fcu*** MOF^[^ ^33^ ^]^ (vide infra). Further insight into the crystallographic structure of MD‐Ce‐UiO‐66 was profiled by cryoelectron microscopy (Cryo‐EM) imaging. The lattice fringes of 1.2 nm, assigned to the (111) plane of ***fcu*** topology, were observed throughout the nanoagent, which was well in line with the simulated ***fcu*** UiO‐**66** model (Figure 1e).

The scanning electron microscopy (SEM) image showed that this as‐prepared MD‐Ce‐UiO‐66 nanoagent possessed a well‐defined spherical structure with a diameter ≈1 µm (Figure S3, Supporting Information). A closer observation disclosed the existing of numerous mesopore and macropore channels (ca. 40–80 nm, Figure 1b). The dendritic surface and highly open porous channels of MD‐Ce‐UiO‐66 were further demonstrated by the transmission electron microscopy (TEM) images (Figure S4, Supporting Information). The TEM image at higher amplification (Figure 1c) revealed that the opening pore channels deviated out from the core of the sphere and distributed radially in all directions. Besides, the pore channels were closely adjacent throughout the whole nanoparticle with a pore wall of ∼10 nm (Figure 1d), which can maximize the mass transfer rate and the accessibility of the interior Ce active sites. Additionally, the N_2_ adsorption/desorption isotherms (Figure S5, Supporting Information) described the type‐IV isotherm (Figure S5a, Supporting Information) with a large hysteresis loop at high relative pressure (P/P_0_) higher than 0.48, and the simulated pore‐size distributions (Figure S5b–d, Supporting Information) indicated the structural integration of micropore, mesopore and macropore in MD‐Ce‐UiO‐66. Collectively, these results demonstrated that the Ce‐MOF with a hierarchically porous structure and dendritic surface was formulated.

### 2.2 Catalytic Generation of ROS at Ambient Condition

**Figure 2:**
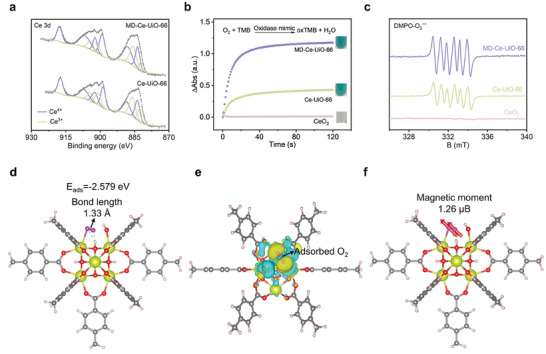
a) The Ce 3d XPS spectra of Ce‐UiO‐66 and MD‐Ce‐UiO‐66. b) The catalytic kinetics curves of bulky CeO_2_, microporous Ce‐UiO‐66, and MD‐Ce‐UiO‐66 under the same Ce amount (15 µg mL^−1^). c) The EPR spectra of DMPO‐O_2_^−•^ for CeO_2_, Ce‐UiO‐66, and MD‐Ce‐UiO‐66. d) The simulated structure of O_2_ molecule adsorbed on the defective Ce_6_ cluster, in which the ligated aqua was pre‐removed. The corresponding adsorption energy and O─O bond length of the adsorbed O_2_ molecule were highlighted. e) The charge density difference of O_2_ molecule adsorbed on defective Ce_6_ cluster. f) The magnetic moment of O_2_ molecule adsorbed on defective Ce_6_ cluster. Red ball represented O atom; purple ball represented adsorbed O atom; yellow ball represented Ce atom; gray ball represented C atom; pink ball represented H atom.

We first inspected the Ce valence of MD‐Ce‐UiO‐66 by X‐ray photoelectron spectroscopy (XPS). At the same time, the standard Ce‐UiO‐66 was also synthesized as a comparison (Figure S6, Supporting Information). The C, O, and Ce elements were recorded in both samples (Figure S7, Supporting Information), and the high‐resolution Ce 3d spectra disclosed the presence of Ce^3+^ and Ce^4+^ in both MD‐Ce‐UiO‐66 and Ce‐UiO‐66 samples (**Figure** **2**a). The Ce^4+^/Ce^3+^ ratio (Table S2, Supporting Information) of MD‐Ce‐UiO‐66 was calculated to be 1.68, which was close to that of standard Ce‐UiO‐66 (1.69), indicating that the pore and surface engineering in MD‐Ce‐UiO‐66 could not alter the chemical compositions and the valences of Ce_6_ clusters. The closer structural insights by thermogravimetric analysis (TGA) revealed that the ligand‐missing defects were formed in both MD‐Ce‐UiO‐66 (0.52 linker deficiency per Ce_6_ cluster) and Ce‐UiO‐66 (1.04 linker deficiency per Ce_6_ cluster) (detailed calculation method was described in Quantitative analysis of TGA and Figure S8, Supporting Information). Such defect formation is also observed in Zr‐UiO‐66 reported in the previous work.^[^ ^33^, ^34^, ^35^ ^]^ This can be interpreted by the fact that the highly ligand‐connected number and dense topology of UiO‐66 trends to form ligand‐ or cluster‐missing defects. Of specific note, these defects favor the exposure of Ce active sites, which enables the molecular oxygen binding and consequently the catalytic generation of ROS (vide infra).

The oxidase‐like activity was estimated using 3,3′,5,5′‐tetramethylbenzidine (TMB) as the oxidase substrate. TMB can be oxidized by O_2_ under the catalytic function of oxidase, and the catalytic product has a blue color, which is easy to be monitored by UV–vis spectrometer. Both Ce‐UiO‐66 and MD‐Ce‐UiO‐66 presented the catalytic activity for TMB oxidization at ambient condition, without the extra addition of chemical reagents or light irradiation (Figure 2b). In addition, the catalytic activity was not affected under either light irradiation or dark conditions (Figure S9, Supporting Information), suggesting a spontaneous catalytic process. The initial catalytic rate and conversion (at 90 s) of MD‐Ce‐UiO‐66 were 1839‐fold and 96.71‐fold as the bulky CeO_2_ under the same Ce amount (Figure S10, Supporting Information), indicating that the discrete Ce active sites in MD‐Ce‐UiO‐66 were favorable for the catalytic reaction compared to the dense Ce sites in CeO_2_ (Figure 2b). In addition, the MD‐Ce‐UiO‐66 presented 3.01‐fold initial catalytic rate and 2.77‐fold conversion (at 90 s) compared to Ce‐UiO‐66 (Figure 2b). Of note, as it was difficult to control the microemulsion templates, the resulting MD‐Ce‐UiO‐66 had a non‐uniform particle size. We concurrently prepared three batch samples of MD‐Ce‐UiO‐66 (Figure S11a, Supporting Information), all of which presented uneven particle size but formed rich mesopore and macropore channels in each particle (Figures S11b–d, Supporting Information). Upon assessing the oxidase activity of the three batch samples, we found a minimal variation of activity with a low RSD value of 3.2% (Figure S12a, Supporting Information). Additionally, we also conducted triplicate activity tests on the same batch sample and found that they presented negligible differences of activity (RSD = 0.49%, Figure S12b, Supporting Information). These results indicated that the heterogeneity in particle size of MD‐Ce‐UiO‐66 had a negligible impact on its oxidase activity.

Considering that both the Ce_6_ cluster formulation and valence state were identical in these two samples, the catalytic activity enhancement could be ascribed to the consecutive meso‐macropore channels in MD‐Ce‐UiO‐66 that facilitated the mass transfer and rendered the interior Ce active sites more accessible. This view was further supported by the observation of differential activity in MD‐Ce‐UiO‐66 before and after polymer template removal (Figure S13, Supporting Information). MD‐Ce‐UiO‐66 showed 2.88‐fold activity enhancement after polymer template removal, validating the key role of opening meso‐macropore channels for catalytic efficiency.

The mechanism of oxidase‐like catalytic activity was then investigated. To verify that the Ce_6_ cluster was indeed the catalytic center, we first synthesized an isostructural MOF, the Ce_6_ cluster of which was replaced by Zr_6_ cluster (denoted as Zr‐UiO‐66, Figure S14, Supporting Information). No oxidase‐like activity was recorded in Zr‐UiO‐66 (Figure S15, Supporting Information), manifesting that the catalytic center was originated from the Ce_6_ cluster in MD‐Ce‐UiO‐66. Under the N_2_ atmosphere, we found that the catalytic reaction of MD‐Ce‐UiO‐66 and Ce‐UiO‐66 completely stopped (Figure S16, Supporting Information), indicating that O_2_ was the indispensable electron acceptor in the oxidase‐like reaction. Further insight into the catalytic mechanism was surveyed by density functional theory (DFT) calculation. The anterior TGA analysis implied that the linker‐missing defect was formed in the Ce_6_ cluster of MD‐Ce‐UiO‐66 (Figure S8, Supporting Information), and the defect sites would be ligated by one hydroxo and one aqua ligand, respectively.^[^ ^36^ ^]^ Based on this consideration, we modeled the optimal Ce_6_ cluster with a linker‐missing site, onto which one hydroxo and one aqua ligand was ligated (Figure S17, Supporting Information). The calculated O_2_ adsorption energy (−2.579 eV, Figure 2d) onto this defective Ce site was much lower than the original aqua ligand adsorption energy (−1.183 eV, Figure S17, Supporting Information), indicating that the adsorption of O_2_ molecule onto this defective Ce site was energetically favorable. The charge density difference image suggested that the electron transferred from Ce_6_ cluster to the bonded O_2_ molecule, as well evidenced by the electron density increased in the O_2_ molecule while reduced in the Ce atoms (Figure 2e). Furthermore, the O‐O bond length of the bonded O_2_ molecule was determined to be 1.33 Å (Figure 2d) and the total magnetic moment of bonded O_2_ was ≈1.26 μB (Figure 2f), both of which were well in line with the characteristic parameters of a superoxide radical.^[^ ^14^ ^]^ These theoretical calculations revealed that the highly accessible Ce site in MD‐Ce‐UiO‐66 facilitated the catalytic conversion of O_2_ to superoxide radical, attributing to the distinct valence electronic structure of Ce (4f^1^5d^1^6s^2^) favoring the catalytic cycle of Ce^3+^/Ce^4+^.^[^ ^24^ ^]^ A closer examination of ROS species was carried out by the electron paramagnetic resonance (EPR) experiment, which indeed identified the strong signal of superoxide radical (O_2_^−•^) using (5,5‐diemthyl‐1‐pyrroline N‐oxide (DMPO) as a spin‐trap agent in both the catalytic reaction of MD‐Ce‐UiO‐66 and Ce‐UiO‐66 (Figure 2c). In contrast, almost no O_2_^−•^ signal was detected in CeO_2_ under the same Ce amount, well in line with its poor oxidase‐like catalytic activity. At the same time, the signal of hydroxyl radical (•OH) using DMPO as a spin‐trap agent was also identified (Figure S18a, Supporting Information), whereas no singlet oxygen (^1^O_2_) was found using 2,2,6,6‐tetramethylpiperidine (TEMP) as the spin‐trap agent during the catalytic reaction (Figure S18b, Supporting Information). Based on these observations, we infer the following catalytic process occurring at the Ce sites: After the adsorption of O₂ onto the Ce sites, an electron is transferred from the Ce site to the O₂ molecule, leading to the formation of O₂⁻^•^. Subsequently, the H₂O molecule is oxidized to •OH at the Ce site, which simultaneously regenerates the Ce sites for further catalysis.

The durability of this MD‐Ce‐UiO‐66 for ROS catalytic generation was then examined. As seen in Figure S19a,b (Supporting Information), MD‐Ce‐UiO‐66 could reserve more than 85% of its original oxidase‐like activity after soaking it in water for 12 d, and the catalytic abilities for O_2_^−•^ and •OH generation were also well retained (Figure S20, Supporting Information), attributing to the stable crystallographic structure (Figure S21, Supporting Information) and the negligible change of Ce constituents (Figure S22 and Table S3, Supporting Information). The long‐lasting activity of this MD‐Ce‐UiO‐66 was further estimated by storing it at room temperature up to 300 d. It still could maintain 70% catalytic activity (Figures S19c,d, Supporting Information), with a preserved catalytic function for O_2_^−•^ and •OH generation (Figure S23, Supporting Information).

### 2.3 The Interfacial Interaction Between MD‐Ce‐UiO‐66 and Bacteria

Apart from the catalytic activity, it entails the interfacial interactions between the materials and bacteria to make full use of the short‐life ROS. In nature, the macrophages in the human immune system can effectively capture bacteria, benefiting by its uneven surface which enhances the contacting area and binding affinity toward bacteria.^[^ ^37^, ^38^ ^]^ Previously reports have found that the electrophilic Ce can readily bind with electron‐rich phosphate groups,^[^ ^39^, ^40^ ^]^ which are abundantly expressed on the cytomembrane. Therefore, we speculated that the dendritic‐shaped surface engineered in MD‐Ce‐UiO‐66 might promote interfacial interactions with bacteria. To verify this, a batch of Ce‐MOFs with various morphologies were synthesized, including the 2D Ce‐BTB‐MOL, cuboidal Ce‐PCN‐224, clavate Ce‐NU‐1000 and octahedral Ce‐MOF‐808 (Figures S24 and S26, Supporting Information). All these Ce‐MOFs presented comparable Ce^4+^/Ce^3+^ constituents (Figures S27 and S28, and Table S2, Supporting Information), and also held the oxidase‐like activity at ambient condition (Figure S29, Supporting Information), owing to the generation of highly active O_2_^−•^ and •OH (Figure S30, Supporting Information). In addition, a similar catalytic mechanism like MD‐Ce‐UiO‐66 was also verified in these Ce‐MOFs (Figures S31–S34, Supporting Information). Of specific note, our MD‐Ce‐UiO‐66 still presented the highest catalytic activity (Figure S35, Supporting Information), ascribed to the widespread meso‐macropore channels favoring the mass transfer and rendering the interior Ce sites more accessible.

**Figure 3:**
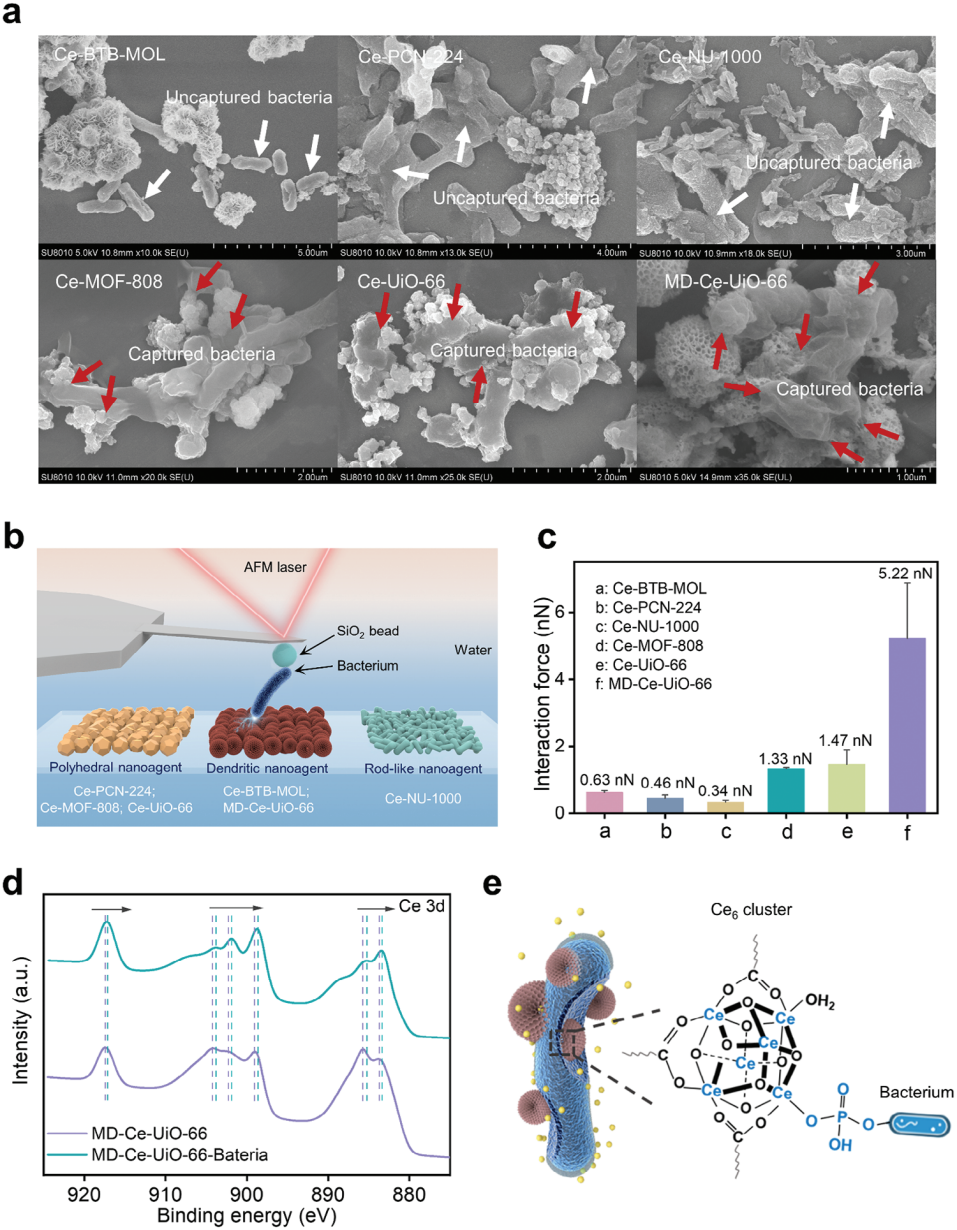
a) SEM images showing the interaction between the Ce‐MOFs and bacteria. b) The AFM set up for the interaction force estimations between various *E. coli* and different nanoagnets. c) The estimated interaction forces by AFM force‐distance curves. d) The Ce 3d XPS spectra of MD‐Ce‐UiO‐66 before and after incubation with *E. coli*. e) The schematic illustration of molecular interactions between the Ce_6_ cluster of MD‐Ce‐UiO‐66 and the phosphate moieties of bacteria cytomembrane.

The interfacial interaction between *Escherichia coli* (*E. coli*) bacteria and these Ce‐MOFs was then examined. We found that the surface charges of the Ce‐MOFs were different. Ce‐PCN‐224, Ce‐NU‐1000, and Ce‐BTB‐MOL possessed negative surface charges while MD‐Ce‐UiO‐66, Ce‐UiO‐66, and Ce‐MOF‐808 had positive surface charges (Figure S36, Supporting Information). This differential surface charge was plausible caused by the different surface termination patterns of Ce_6_ cluster termination and carboxylate linker termination in these MOFs. In this regard, MD‐Ce‐UiO‐66, Ce‐UiO‐66, and Ce‐MOF‐808 were easy to adhere negatively charged bacteria through electrostatic attraction, as evidenced by the SEM images (**Figure** **3**a). While Ce‐PCN‐224, Ce‐NU‐1000, and Ce‐BTB‐MOL exhibited very limited ability for bacteria capture because of the electrostatic repulsion (Figure 3a). A further inspection of the binding affinity between these Ce‐MOFs and bacteria was conducted by atomic force microscopy (AFM) (Figure 3b; Figure S37, Supporting Information). Both positively charged Ce‐MOFs of MD‐Ce‐UiO‐66, Ce‐UiO‐66, and Ce‐MOF‐808 presented higher binding forces toward bacteria compared to the negatively charged counterparts of Ce‐PCN‐224, Ce‐NU‐1000, and Ce‐BTB‐MOL. Of note, although the surface potentials were similarly positive among MD‐Ce‐UiO‐66, Ce‐UiO‐66, and Ce‐MOF‐808, the MD‐Ce‐UiO‐66 dominated the strongest interaction with bacteria, with an average binding force of 5.22 N, which was 3.55‐fold and 3.92‐fold higher than that of Ce‐UiO‐66 and Ce‐MOF‐808, respectively (Figure 3c; Figure S38, Supporting Information). Additionally, the confocal laser scanning microscope (CLSM) experiments were carried out to validate the strong interaction between MD‐Ce‐UiO‐66 and bacteria. The overlap of green and red fluorescence was observed after immediately mixing SYTO9‐labeled *E. coli* (green fluorescence) with rhodamine‐loaded MD‐Ce‐UiO‐66 (RhB‐MD‐Ce‐UiO‐66, red fluorescence, Figure S39, Supporting Information), suggesting the high bacteria‐capturing ability of the MD‐Ce‐UiO‐66. Furthermore, the Cryo‐EM imaging showcased that the rod‐shaped *E. coli* was tightly adhered to the dendritic‐shaped surface of MD‐Ce‐UiO‐66 (Figure S40, Supporting Information). At the same time, in the high‐resolution Ce 3d XPS spectra, we found that the binding energy of Ce 3d was shifted to lower energy after the interfacial interaction formation between MD‐Ce‐UiO‐66 and bacteria (Figure 3d), indicating that the molecular interactions between the surface electrophilic Ce site on MD‐Ce‐UiO‐66 and the electron‐rich phosphate moieties of bacteria cytomembrane (Figure 3e).^[^ ^38^, ^39^ ^]^

### 2.4 In Vitro Antibacterial Performances

**Figure 4:**
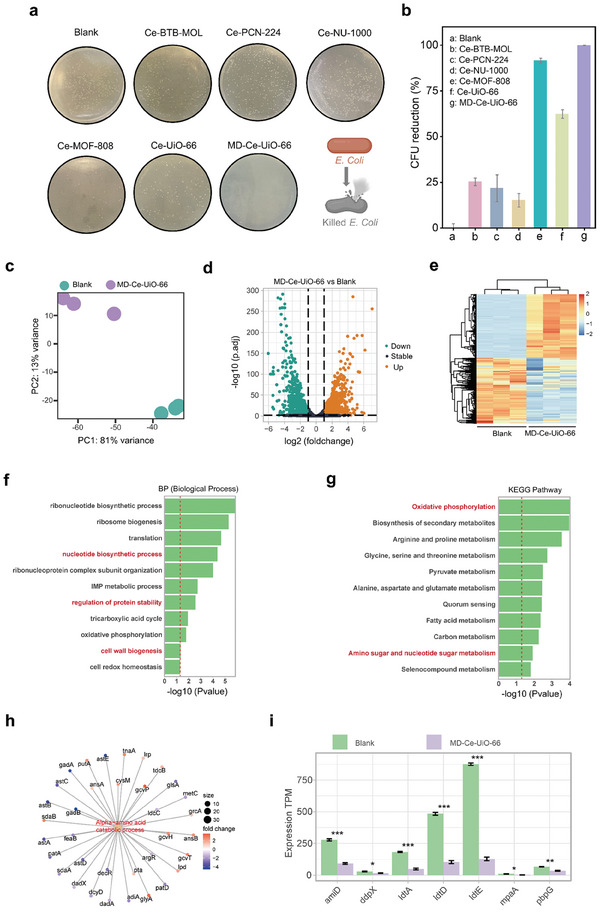
a) Digital images of bacterial colonies of *E. coli* after treatments with different Ce‐MOFs and b) the corresponding bacterial viabilities. c) Principal components analysis in blank group and MD‐Ce‐UiO‐66‐treated group. d) Volcano plot of upregulated and downregulated genes in blank group and MD‐Ce‐UiO‐66‐treated group. e) Heatmap showing the differentially expressed genes between MD‐Ce‐UiO‐66‐treated group and the blank group. f) Bar plot showing the biological processes differentially regulated by MD‐Ce‐UiO‐66 compared with the blank group by gene ontology (GO) enrichment analysis. g) Bar plot showing the pathways differentially regulated by MD‐Ce‐UiO‐66 compared with the blank group through Kyoto Encyclopedia of genes and genomes (KEGG) pathway enrichment analysis. h) Expression changes between MD‐Ce‐UiO‐66‐treated group and the blank group in the Alpha−amino acid catabolic process. i) The expression of cell wall organization‐related genes in MD‐Ce‐UiO‐66‐treated group and blank group.

After validating the dual functions of MD‐Ce‐UiO‐66 for ROS catalytic generation and bacteria capture, we then examined the antibacterial performances against the representative gram‐negative bacteria of *E. coli* at ambient condition using a classical plate counting method. As shown in **Figure** **4**a, incubating the *E. coli* with different Ce‐MOFs under the same Ce amount, the number of bacterial colonies in Ce‐BTB‐MOL (colony‐forming units reduction, CFU reduction = 25.2%, Figure 4b), Ce‐PCN‐224 (CFU reduction = 21.7%, Figure 4b) and Ce‐NU‐1000 (CFU reduction = 15.1%, Figure 4b) were only slightly lower than that of the blank group, implying that the antibacterial efficacies were feeble attributing to the inferior catalytic activities and poor bacteria‐capturing capacities (Figure 3a). Whereas the Ce‐MOF‐808 (CFU reduction = 91.5%, Figure 4b) and Ce‐UiO‐66 (CFU reduction = 62.3%, Figure 4b) presented an enhanced performance for combating *E. coli* bacteria, owing to their enhanced interactions with bacteria (Figure 3a). Of specific note, our MD‐Ce‐UiO‐66 showed nearly 100% antibacterial efficacy (CFU reduction = 99.9%, Figure 4b). At the same time, the two‐color living/dead staining experiment confirmed that when incubating *E. coli* bacteria with Ce‐MOF‐808 and Ce‐UiO‐66, respectively, partial bacteria were still survive (Figure S41, Supporting Information), in line well with classical plate counting results (Figure 4b). While in the case of using MD‐Ce‐UiO‐66 under the same Ce amount, no survival *E. coli* bacteria was found, suggesting the best antibacterial ability.

To deeply understand the molecular mechanism for the antibacterial activity of MD‐Ce‐UiO‐66, RNA sequencing (RNAseq) technology was applied to unveil the relevant pathways involved in the treatment of *E. coli* with MD‐Ce‐UiO‐66. The distinct differences in transcriptome landscapes between the MD‐Ce‐UiO‐66‐treated group and the blank group were found in the principal component analysis (PCA), indicating the obvious changes in gene expression patterns of *E. coli* after MD‐Ce‐UiO‐66 treatment (Figure 4c). Further analysis revealed that after MD‐Ce‐UiO‐66 treatment, there were a total of 1451 diversely expressed genes (DEGs) (flod change > = 2, adj. *p*‐value < 0.01), containing 716 upregulated and 735 downregulated genes (Figure 4d). Figure 4e presented a heat map of the hierarchical clustering of DEGs, showing the expression levels of each gene in the MD‐Ce‐UiO‐66‐treated group and the blank group. Enrichment analysis indicated that several signaling pathways, such as transcriptional regulation, nucleotide metabolism, protein homeostasis, and cell wall maintenance, were enriched by treating with MD‐Ce‐UiO‐66 (Figure 4f,g). Notably, a large part of genes associated with amino acid metabolism were suppressed, suggesting that MD‐Ce‐UiO‐66 affected the protein utilization in bacteria and triggered the depletion of biological processes including energy metabolism and oxidative phosphorylation. In addition, we noticed the significant inhibition of genes associated with bacterial cell wall maintenance (Figure 4h,i), which might be caused by the strong adhesion of MD‐Ce‐UiO‐66 on *E. coli*.

### 2.5 Self‐Antimicrobial Mask Workable in Different Occasions

**Figure 5:**
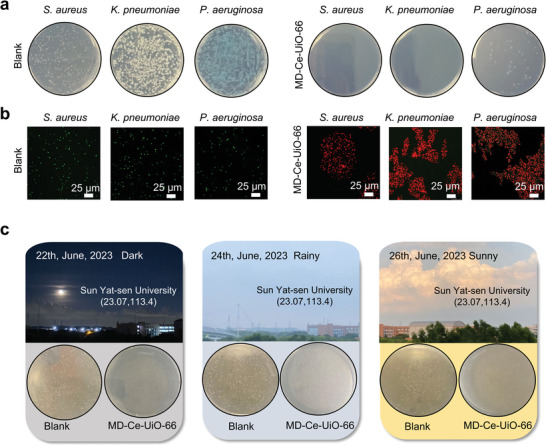
Self‐antimicrobial mask workable in different occasions. a) Digital images of bacterial colonies of *S. aureus*, *K. pneumoniae* and *P. aeruginosa* in the blank groups and the MD‐Ce‐UiO‐66 groups, and b) the corresponding SYTO9/PI two‐color fluorescent images for the live (green fluorescence) and dead (red fluorescence) bacterial staining assay of different bacteria. Scale bar: 25 µm. c) The antibacterial plate assays (for *E. coli*) showing the antibacterial performances of MD‐Ce‐UiO‐66 in the dark and on rainy and sunny days.

We next investigated the antibacterial activities of MD‐Ce‐UiO‐66 against other bacteria including *Staphylococcus aureus* (*S. aureus*), *Klebsiella pneumoniae* (*K. pneumoniae*) and *Pseudomonas aeruginosa* (*P. aeruginosa*), the most common antibiotic‐resistant pathogens causing hospital‐associated infections. At ambient condition, it was found that the colony counts in the experimental groups of *S. aureus* (CFU reduction = 97.6%), *K. pneumoniae* (CFU reduction = 100%) and *P. aeruginosa* (CFU reduction = 94.7%) treated with MD‐Ce‐UiO‐66 were much lower than the blank groups without MD‐Ce‐UiO‐66 (**Figures** **5**a and S42a, Supporting Information). Furthermore, the two‐color dead/live staining analysis validated the nearly entire dead bacteria with red fluorescence (labeled by propidium iodide, PI, λ_ex_ = 488 nm, λ_em_ = 635 nm) in all experimental groups treated by MD‐Ce‐UiO‐66. As a striking contrast, numerous alive bacteria with bright green fluorescence (labeled by SYTO‐9, λ_ex_ = 488 nm, λ_em_ = 500 nm) were identified in the blank groups (Figure 5b). These results verified the broad‐spectrum antibacterial performance of our MD‐Ce‐UiO‐66 at ambient condition.

We next carried out antibacterial plate‐killing assays to verify the bacteriostatic efficiencies of MD‐Ce‐UiO‐66 in the dark, as well as on rainy and sunny days (Figure 5c). As indicated in agar plate images, the bacterial survival rates in all experimental groups treated by MD‐Ce‐UiO‐66 were close to zero and the CFU reduction in the dark and on rainy and sunny days were calculated to be 99.9%, 99.9% and 99.8%, respectively (Figure S42b, Supporting Information), demonstrating the spontaneous antimicrobial behavior of this MD‐Ce‐UiO‐66 without the dependency on light energy.

**Figure 6:**
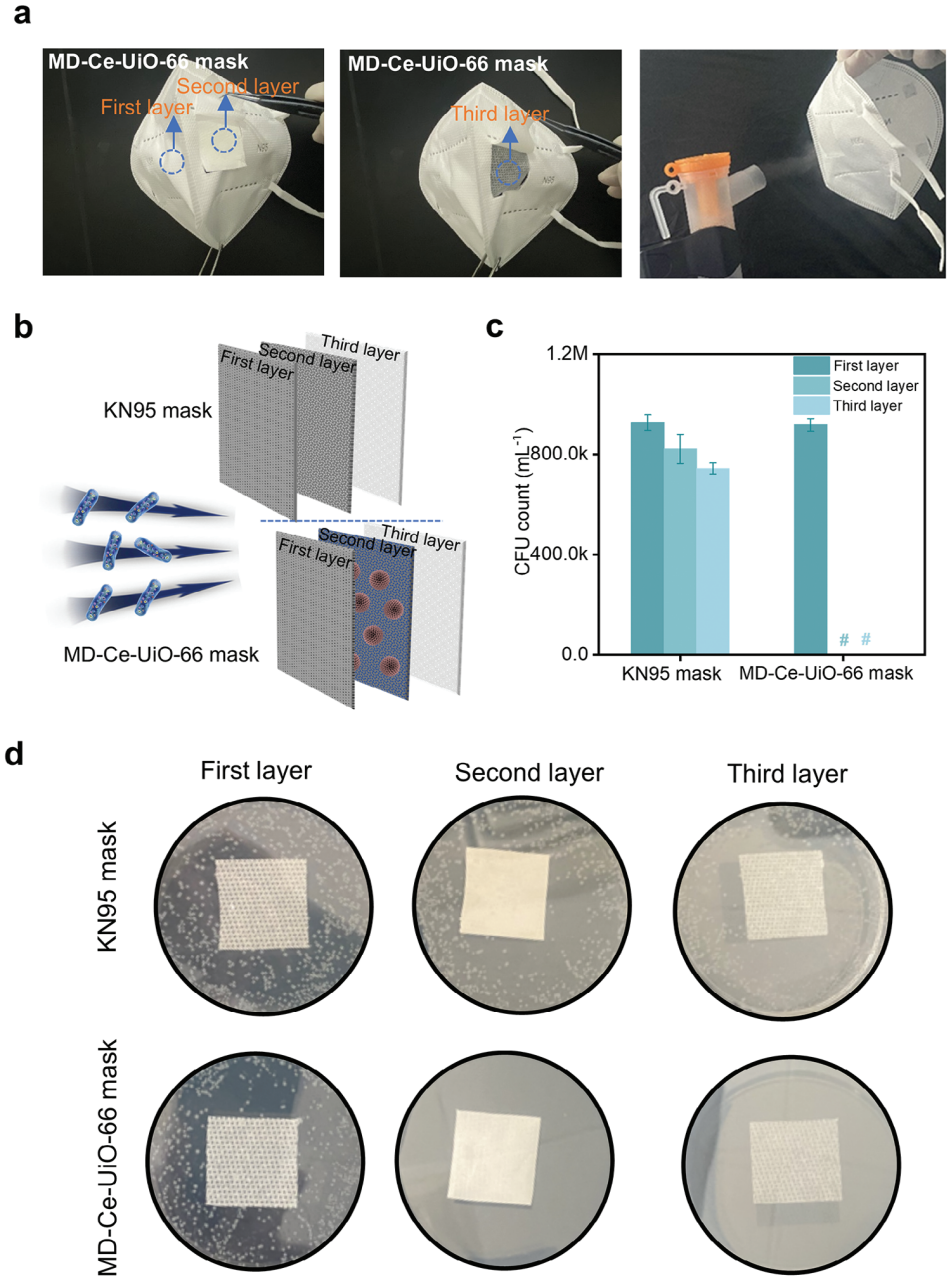
a) Photographs of bioaerosol generation apparatus as well as the trilaminar KN95 mask and MD‐Ce‐UiO‐66 mask. b) Schematic representation of the bioaerosol passing across the face masks. (c, d) *E. coli* residual levels on the first, second, and third layers of our MD‐Ce‐UiO‐66 mask and the KN95 mask counterpart. # in c) indicates an undetectable level of CFU mL^−1^ count.

The feasibility of using MD‐Ce‐UiO‐66 as a biocidal coating for the fabrication of personal protective equipment was then examined. As a proof of concept, we integrated MD‐Ce‐UiO‐66 into commercially available KN95 face masks through a simple spray deposition method. The formed MD‐Ce‐UiO‐66 mask consisted of three layers, of which the first and third layers were the woven fabric similar to KN95 masks and the middle layer was the MD‐Ce‐UiO‐66‐coated woven fabric, with a loading of 5 mg MD‐Ce‐UiO‐66 per 3×3 cm^2^ square woven fabric (Figure S43, Supporting Information). The sterilization efficiency of this conceptual MD‐Ce‐UiO‐66 face mask was evaluated. In this sterilization experiment, the outermost surfaces of the MD‐Ce‐UiO‐66‐integrated masks and original KN95 face masks were sprayed with the *E. coli* aerosols for 5 min using an aerosol generator (flow rate: 0.3 mL min^−1^, **Figure** **6**a,b) for simulating the propagation process of the respiratory droplet. After incubating at 37 °C for 4 h, each layer of the as‐treated masks was then soaked into sterile water and vortexed. The resulting supernatants were further diluted and plated onto Luria‐Bertani agar plates, followed by incubation at 37 °C overnight for residual analysis of adhered visible colonies. As seen in Figure 6c,d, we noticed that many living bacteria were visible on the first layers of both the MD‐Ce‐UiO‐66 mask and KN95 mask. Whereas in our MD‐Ce‐UiO‐66 mask, no alive bacterium was identified in the middle or third layers that close to or in direct contact with the human face. In stark contrast, numerous living bacteria are still located on the middle and third layers of the KN95 mask (Figure 6c,d). Furthermore, we conducted a series of antimicrobial experiments on the MD‐Ce‐UiO‐66 face mask under varying humidity and temperature conditions. Specifically, we tested the mask in the following environments: 53% humidity at 23.5 °C; 36% humidity at 36.9 °C; 86% humidity at 10.2 °C. As illustrated in Figure S44 (Supporting Information), in our MD‐Ce‐UiO‐66 masks, no alive bacterium was identified in the middle or third layers that close to or in direct contact with the human face, regardless of varying humidity levels and temperatures, indicating that our MD‐Ce‐UiO‐66 mask showed considerable antibacterial activity under different humidity and temperature conditions.

It was worth noting that the preparation of this MD‐Ce‐UiO‐66 was not associated with any energy‐intensive steps and the whole synthesis procedure was completed within 40 min in an aqueous solution at 40 °C. The total price of MD‐Ce‐UiO‐66 for one face mask is ca. $0.4, and the cost of this antimicrobial MD‐Ce‐UiO‐66 mask is estimated to be ca. $0.43 per mask (detailed analysis was described in The cost analysis of MD‐Ce‐UiO‐66 mask and Table S4, Supporting Information). Considering the functional stability for ROS catalytic generation over 300 d, this versatile and cost‐efficient MD‐Ce‐UiO‐66 would hold huge potential for scale‐up fabrication of multifunctional protective equipment. We also assessed the biosafety of the MD‐Ce‐UiO‐66 mask by evaluating both the water stability of the loaded particles and their migration potential. First, we studied the structural integrity of MD‐Ce‐UiO‐66 in an aqueous solution. In this experiment, 5 mg of MD‐Ce‐UiO‐66 powder was immersed in 1 mL of water for three days. ICP analysis showed that the Ce ratio in the supernatant was only 0.2%, indicating that the MD‐Ce‐UiO‐66 particles were highly water‐stable and exhibited excellent resistance to leaching in aqueous conditions. In addition, after the bioaerosol simulation experiment, the third layer of MD‐Ce‐UiO‐66 mask, which was in direct contact with the human face, was removed and examined by SEM. It showed the absence of MD‐Ce‐UiO‐66 powder (Figure S45, Supporting Information), indicating that the MD‐Ce‐UiO‐66 coated in the middle layer could not migrate to the innermost layer of the mask. Based on these results, we concluded that the MD‐Ce‐UiO‐66‐loaded mask was biosafe.

## 3 Conclusion

In summary, by virtue of pore and surface engineering, we develop an antimicrobial Ce‐MOF intensifying ROS catalytic activity and bacteria entrapment. This synergistic effect can maximally harness the short‐life ROS to inactivate bacteria, achieving a dynamical promotion of antibacterial efficacy against different kinds of pathogens at ambient condition. Note that this antimicrobial Ce‐MOF is functionally stable for up to 300 days and its synthesis is procedurally simple and low‐cost, with the cost of the antimicrobial face mask estimated to be as low as $0.43 per face mask, holding great potential for the scale‐up industrial manufacture. This work provides new insights into the access to next‐generation antibacterial materials by MOF pore‐ and surface‐engineering approach, advancing the development of self‐antibacterial protective devices.

## Conflict of Interest

The authors declare no conflict of interest.

## Supporting information



Supporting Information

## Data Availability

The data that support the findings of this study are available from the corresponding author upon reasonable request.
