# Supplementary material for: A Hierarchical Metal–Organic Framework Intensifying ROS Catalytic Activity and Bacterial Entrapment for Engineering Self‐Antimicrobial Mask
Source: Adv Sci (Weinh). 2024 Dec 16;12(6):2410703. doi: 10.1002/advs.202410703 (PMC11809350; doi:10.1002/advs.202410703)
Supplement: Supplementary file 1 — Supporting Information [file ADVS-12-2410703-s001.docx]

**Supporting Information**

**A Hierarchical Metal-Organic Framework Intensifying ROS Catalytic Activity and Bacterial Entrapment for Engineering Self-Antimicrobial Mask**

*Wei Huang, Haitao Yuan, Huangsheng Yang, Yujian Shen, Lihong Guo, Ningyi Zhong, Tong Wu, Yong Shen, Guosheng Chen*, Siming Huang*, Li Niu*, and Gangfeng Ouyang****

W. Huang, Prof. G. Chen, Prof. L. Niu, Prof. G. Ouyang

School of Chemical Engineering and Technology, Southern Marine Science and Engineering Guangdong Laboratory (Zhuhai), Sun Yat-sen University

Zhuhai 519082, China

E-mail: chengsh39@mail.sysu.edu.cn (G. Chen); lniu@gzhu.edu.cn (L. Niu); cesoygf@mail.sysu.edu.cn (G. Ouyang)

Y. Yang, Yujian Shen, L. Guo, N. Zhong, Prof. Yong Shen, Prof. G. Chen, Prof. G. Ouyang

School of Chemistry, Sun Yat-sen University

Guangzhou 510006, China

Prof. G. Chen, Prof. G. Ouyang

Sun Yat-sen University School of Chemistry and Guangdong Basic Research Center of Excellence for Functional Molecular Engineering, Sun Yat-sen University

Guangzhou 510006, China

H. Yuan

Center for Drug Research and Development Guangdong Provincial Key Laboratory of Advanced Drug Delivery System, Guangdong Pharmaceutical University

Guangzhou 510006, P. R. China

T. Wu

Department of Radiology, The Third Affiliated Hospital of Southern Medical University, Southern Medical University

Guangzhou 510630, China

Prof. S. Huang

Guangzhou Municipal and Guangdong Provincial Key Laboratory of Molecular Target & Clinical Pharmacology, the NMPA and State Key Laboratory of Respiratory Disease, School of Pharmaceutical Sciences and the Fifth Affiliated Hospital, Guangzhou Medical University

Guangzhou 511436, China.

E-mail: huangsm@gzhmu.edu.cn (S. Huang)

**Experimental Section**

**Reagent and materials**

Sodium perchlorate monohydrate (NaClO_4_·H_2_O，99%), zirconium (IV) chloride (ZrCl_4_, 99.9%), meso-Tetra(4-carboxyphenyl)porphine (H_6_TCPP, 97%), formic acid (HCOOH, 96%), acetic acid (AA, 99.9%), glycine (99%), sodium chloride (NaCl, 99.5%) trifluoroacetic acid (TFA, 99%), benzene-1,3,5-tricarboxylic acid (H_3_BTC, 98%), poly-L-Lysine solution (PLL, 150000-300000, 0.1% (w/v) in H_2_O), cerium oxide (CeO_2_, 99.99%) and 3,3',5,5'-tetramethylbenzidine (TMB, 99%) were purchased from Aladdin Chemistry Co., Ltd. (Shanghai, China). Cerium ammonium nitrate ((NH_4_)_2_Ce(NO_3_)_6_, 99.99%) and 1,3,6,8-tetrakis(p-benzoic aicd)pyrene (H_4_TBAPy, 97%) were purchased from J&K Scientific Ltd. (Beijing, China). Ploy(ethylene glycol)-block-ploy(propylene glycol)-block-ploy(ethylene glycol) Pluronic®P-123 (P123, average Mn ~5800), Pluronic®F-127 (F127, BioReagent), Zirconium (IV) oxychloride (ZrOCl_2_.8H_2_O, 98%), and 1,4-dicarboxybenzene (BDC, 98%) were obtained from Sigma-Aldrich. 1,3,5-tri(4-carboxyphenyl)benzene (H_3_BTB, 98%), monodisperesed silica microspheres (SiO_2_, ~ 3.0 μm， 2.5% w/v), glutaraldehyde (2.5%), rhodamine B isothiocyanate (RhB) were supplied by Macklin Biochemical Technology Co., Ltd. (Shanghai, China). benzoic acid (BA, 99%) and 5,5-diemthyl-1-pyrroline N-oxide (DMPO, 97%) were supplied by TCI Chemical Industry Development Co., LTD. (Shanghai, China). SYTO 9 was purchased from Thermo Fisher Scientific, Inc. Dimethyl formamide (DMF), hydrochloric acid (HCl), nitric acid (HNO_3_), acetone, ethanol and toluene were purchased from Guangzhou Chemical Reagent Factory (Guangzhou, China). All chemicals and reagents were purchased from commercial sources and used without further purification.

**Characterization**

Powder X-ray diffraction (PXRD) patterns were collected (0.02°/step, 0.06 seconds/step) on a Bruker D8 Advance diffractometer (Cu Kα) at room temperature.

N_2_ adsorption isotherms were collected with a JW-DX Surface Area Analyzer at -196 °C. All the samples were pre-treated under 100 °C for 12 h before measurements.

Thermogravimetric analyses (TGA) were run up to 900 ^o^C under air atmosphere with temperature increasing at 5 ^o^C/min using a TA-Q50 system.

The ultraviolet-visible (UV-vis) absorbance measurement was carried out on a 2800S spectrophotometer (SOPTOP, Shanghai), while the UV-Visible diffuse reflectance spectrum was performed with a 3600 spectrophotometer (Shimadzu, Japan).

Fluorescence spectra were performed on an F-97 Pro fluorescence spectrometer.

Zeta potentials were measured on a Nano ZS 90 system.

The morphology images of the crystals were taken on a SU8010 ultra-high resolution field emission scanning electron microscope (SEM, Hitachi, Japan). Transmission electron microscope (TEM) images were taken on a JEM-2010HR microscope operating at 200 kV.

Electron paramagnetic resonance (EPR) experiment was carried out on Bruker EMX plus 10/12 equipped with Oxford ESR910 Liquid Helium cryostat. The test temperature was set at 5 K.

X-ray photoelectron spectroscopy (XPS) signals were analyzed by a Nexsa X-ray photoelectron spectrometer (Thermo Scientific, MA, USA).

The Ce ratio within MOFs were measured with inductively coupled plasma mass (ICP-MS, Prodigy, Leeman, USA).

Atomic force microscopy (AFM) analysis was performed on an atomic force microscopy (dimension fastscan bio, Bruker).

Fluorescence images were captured by Confocal laser scanning microscope (CLSM 880 NLO, Carl Zeiss, Göttingen, Germany).

Cryo-electron microscopy (Cryo-EM) experiments were performed on a ThermoFisher Scientific Titan Krios G3i electron microscopes operated at 300 kV. The dispersive nanomaterials (in ethanol) were mounted to a carbon-coated TEM-grid and dried under vacuum. The specimen was then dropped into liquid nitrogen and transferred by a Cryo-transfer loader into the microscope. Cryo-EM images were collected by a K3 Summit direct electron detector equipped with a GIF Quantum energy filter (slit width 20 eV) in the counting mode (Bin 0.5). Data acquisition was performed using SerialEM 3.864 with a nominal magnification of 350,000 ×, corresponding to a physical pixel size of 0.34 Å. The dose rate was ~15 counts/pixel/second, and the exposure time in a frame was 0.023 s. Each micrograph stack contains 10 frames (the total exposure time was 0.23 s), and the total dose rate was ca. 30 e-/Å^2^ per micrograph. The motion correction was performed using MotionCorr265 with 2×2 binning, and the non-dose-weighted sum of all frames from each movie was used for all image processing steps. The lattice spacing of MD-Ce-UiO-66 unit cells were analyzed using DigitalMicrograph (Gatan) software.

**Synthesis of Ce-UiO-66**

Ce-UiO-66 was prepared according to a previous literature with some modifications.^[1]^ Firstly, 35.4 mg BDC was dissolved in 1.2 mL DMF, followed by adding a (NH_4_)_2_Ce(NO_3_)_6_ (116 mg) aqueous solution (0.4 mL). Then, the mixture was stirred at 100 ^o^C for 15 min. The resultant solid was isolated by centrifugation and washed with DMF twice and acetone once. Finally, the Ce-UiO-66 powder was dried at 60 ^o^C under vacuum.

**Synthesis of MD-Ce-UiO-66**

The preparation of MD-Ce-UiO-66 was based on a polymer microemulsion template-mediated coordination assembly strategy.^[2]^ First, 100 mg Pluronic®P-123 and 50 mg Pluronic®F-127 were dissolved in 6 mL deionized water. Subsequently, 240 μL toluene was added to the solution mentioned above and thoroughly mixed. Next, 300 mg sodium perchlorate monohydrate and 100 μL acetic acid were added. The microemulsion solution was formed by stirring the mixture. The aforementioned mixture was then supplemented with 548 mg cerium ammonium nitrate and 120 mg 1,4-dicarboxybenzene. After that, the mixture was stirred for 40 min at 40 ^o^C. The resultant solid was isolated by centrifugation and washed with water twice and dimethyl formamide once. The collected solid sample was further soaked in ethanol for two days at 60 ^o^C to remove the microemulsion templates, with daily ethanol renewals. Finally, the MD-Ce-UiO-66 powders were dried overnight at 60 ^o^C under vacuum.

**Synthesis of Ce-BTB MOL**

Ce-BTB-MOL was prepared according to a previous literature with some modifications.^[3]^ Typically, 117 mg (NH_4_)_2_Ce(NO_3_)_6_ was dissolved in 0.4mL water by vigorous stirring at 80 ^o^C, and followed by the dropwise addition of a H_3_BTB (31.3 mg) solution in DMF (1.2 mL). The mixture was stirred at 80 ^o^C for 15 min and then cooled to room temperature. The mixture was reheated to 80 ^o^C and held at this temperature for another 15 min. The resultant solid was centrifuged and washed with DMF three times and acetone once. Finally, the Ce-BTB-MOL powder was dried at 60 ^o^C under vacuum.

**Synthesis of Ce_6_ cluster**

[Ce_6_O_4_(OH)_4_(NH_3_CH_2_COO)_8_(NO_3_)_4_(H_2_O)_6_Cl_8_·8H_2_O] was scaled up from a previously reported protocol.^[4]^ First, 3 g (NH_4_)_2_Ce(NO_3_)_6_ and 0.9 g glycine were dissolved in 2.7 g water. Then, 32 g of a saturated NaCl solution was added and the resulting solution was left at room temperature for 48 h. Finally, the resultant yellow crystals were filtered off and dried at 60 ^o^C under vacuum.

**Synthesis of Ce-PCN-224**

The synthesis of Ce-PCN-224 was based on a previous report.^[5]^ Firstly, 30 mg pre-prepared Ce_6_ cluster was added to 0.3 mL water and the obtained suspension was immediately added to a solution of H_6_TCPP (11.9 mg) and BA (300 mg) in 1.2 mL DMF. This mixture was then stirred at 100°C for 15 min. The resultant precipitate was removed from the mother liquor by centrifugation. The collected Ce-PCN-224 was washed with DMF twice and acetone once, and finally dried at 60 ^o^C under vacuum.

**Synthesis of Ce-NU-1000**

The synthesis of Ce-NU-1000 was based on a previous report.^[5]^ Firstly, 23.5 mg pre-prepared Ce_6_ cluster was added to 0.25 mL water and the obtained suspension was immediately added to a solution of H_4_TBAPy (10 mg) and BA (676 mg) in 2 mL DMF. This mixture was then stirred at 100 °C for 15 min. Finally, the resultant precipitate was removed from the mother liquor by centrifugation. The collected Ce-NU-1000 was washed with DMF twice and acetone once, and finally dried at 60 ^o^C under vacuum.

**Synthesis of Ce-MOF-808**

Ce-MOF-808 was prepared according to the reported method with some modifications.^[6]^ First, 22.4 mg H_3_BTC was introduced into the mixture of DMF/HCOOH (1.2 mL/0.257 mL), followed by adding a (NH_4_)_2_Ce(NO_3_)_6_ (174 mg) aqueous solution (0.6 mL). The mixture was further stirred at 100 °C for 15 min. The white precipitate was centrifuged from mother liquor, and washed with DMF twice and acetone once. Finally, the Ce-MOF-808 powder was dried at 60 ^o^C under vacuum.

**Synthesis of Zr-UiO-66**

The synthesis of Zr-UiO-66 was based on a previous report.^[7]^ First, 40.8 mg ZrCl_4_ and 26.6 mg BDC were ultrasonically dispersed in the mixture of DMF/AA (10 mL/0.5 mL). Following that, the mixed solution was sealed into a 20 mL vessel and allowed to react at 120 °C for one day. Finally, the as-synthesized Zr-UiO-66 was centrifuged and washed with acetone and dried at 60 °C under vacuum.

**Synthesis of Zr-PCN-224**

The synthesis of Zr-PCN-224 was based on a previous report.^[8]^ First, 30 mg ZrCl_4_, 10 mg H_6_TCPP, and 400 mg BA were ultrasonically dissolved in 2 mL DMF. Then, the mixture was heated in an oven at 120 °C one day. After cooling down to room temperature, the resultant cubic dark purple crystals were harvested by centrifugation and washed with DMF twice and acetone once. Finally, the Zr-PCN-224 powder was dried at 60 ^o^C under vacuum.

**Synthesis of Zr-NU-1000**

The synthesis of Zr-NU-1000 was based on a previous report with some modifications.^[9]^ Firstly, 97 mg ZrOCl_2_∙8H_2_O and 1.6 g BA were dissolved in 8 mL DMF and heated in an oven at 100 °C to obtain Solution A. 20 mg H_4_TBAPy was dissolved in 8 mL DMF and heated in an oven at 100 °C to obtain Solution B. After that, 1 mL of solution A and 1 mL of solution B were mixed and placed into an oven at 120 °C for 1 h. After cooling down to room temperature, the resultant suspension was isolated by centrifugation and washed with DMF twice and acetone once. Finally, the Zr-NU-1000 powder was dried at 60 ^o^C under vacuum.

**Synthesis of** **Zr-MOF-808**

The synthesis of Zr-MOF-808 was carried out using a previously reported procedure with slight modifications.^[10]^ First, 289 mg ZrOCl_2_·8H_2_O and 63 mg H_3_BTC were introduced in a mixture of DMF/HCOOH (9 mL/9 mL). The suspension was further sonicated for 10 min. After sonication, the mixture was kept at 100 °C for 18 h. Finally, the as-synthesized Zr-MOF-808 was centrifuged and washed with DMF twice and acetone once, and dried at 60 °C under vacuum.

**Quantitative analysis of TGA**

The quantitative analysis of TGA data for Ce-UiO-66 and MD-Ce-UiO-66 was based on an important assumption that the residues in each TGA experiment are pure CeO_2_, with reference to the previous literature.^[11]^

The following reaction is considered as the complete combustion of defect-free Ce-UiO-66, Ce_6_O_6_(BDC)_6_ (Equation 1):

$\mathrm{Ce}_{6}O_{6}{(BDC)}_{6} \left( s \right)+45 O_{2} \left( g \right)\to6 \mathrm{CeO}_{2}+48 \mathrm{CO}_{2} \left( g \right)+12 H_{2}O (g)$ (1)

The molar mass of Ce_6_O_6_(BDC)_6_ is 1921.48 g mol^-1^, with 1.861 higher than that of solid residue-6 moles of CeO_2_ (6 × 172.12 = 1032.70 g mol^-1^). Therefore, if the end weight (i.e. the weight at 900 °C) of a TGA running on Ce-UiO-66 is normalized to 100 % (defined as W_End_), the TGA plateau that represents the empty, solvent free, and dehydroxylated MOF, should ideally be located at 186.1 % (defined as W_Ideal_._Plat_.) on the TGA trace. However, it is typically much lower than this theoretical weight, which means the Ce-UiO-66 framework is lighter than that formulated in the ideal equation. Such an observation contributes to the initial hypothesis that the Ce-UiO-66 framework might be linker deficient.^[12]^

We set out to quantify the deficiencies in our synthesized Ce-UiO-66 and MD-Ce-UiO-66 samples. It is assumed that each missing linker is charge compensated by an additional oxide anion on the cluster, resulting in an average composition as follows:

$$\mathrm{Ce}_{6}O_{6+x}\left( \mathrm{BDC} \right)_{6-x}$$

Where: x represents the number of linker deficiencies per Ce_6_ formula unit.

The weight contribution per BDC linker (Wt. PL_Theo._) can be arrived at by simply taking the difference between the TGA plateau of the ideal dehydroxylated material (W_Ideal_._Plat_. = 186.1 %) and the end weight of the TGA run (W_End_ = 100 %), and dividing by the number of linkers in the ideal Ce_6_ formula unit (NL_Ideal._ = 6):

$$\begin{aligned} {Wt. PL}_{Theo.}=\frac{\left( W_{Ideal.plat.}-W_{\mathrm{End}} \right)}{\mathrm{NL}_{Ideal.}}\#\left( 2 \right) \end{aligned}$$

As discussed above, inserting these values (W_Ideal_._Plat_. = 186.1 %, W_End_ = 100 % and NL_Ideal._ = 6) into Equation (2) allows Wt. PL_Theo_. to be determined:

$${Wt. PL}_{Theo.}=\frac{(186.1\%-100\%)}{6}=14.35\%$$

It can determine the experimental number of linkers per defective Ce_6_ formula unit (NL_Exp._ = 6-x) by rearranging Equation (2) and replacing the idealized NL_Ideal._ and W_Ideal_._Plat_. values with the real experimental values, NL_Exp._ and W_Exp.Plat._:

$$\begin{aligned} \mathrm{NL}_{Exp.}=\left( 6-x \right)=\frac{\left( W_{Exp.plat.}-W_{\mathrm{End}} \right)}{{Wt. PL}_{Theo.}}\#\left( 3 \right) \end{aligned}$$

Since NL_Exp._ =6-x, the number of linker deficiencies per Ce6 formula unit (x) is effortless to be calculated by rearranging Equation (3):

$$\begin{aligned} x=6-\mathrm{NL}_{Exp.}=6-\frac{\left( W_{Exp.plat.}-W_{\mathrm{End}} \right)}{{Wt.PL}_{\mathrm{Theo}}}\#\left( 4 \right) \end{aligned}$$

As described above, the values of Wt. PL_Theo_. (14.35 %) and W_End_ (100 %) are already known. In the Equation (4), the W_Exp.Plat._ is the only unknown value, and thus the correct choice of plateau is the most vital part of the analysis. The calculation processes for MD-Ce-UiO-66 and Ce-UiO-66 are described in **Figure S8**.

**1.15. Ce concentration measurement**

The Ce content in Ce-MOF materials was evaluated by ICP-MS. In a typical test, about 1 mg of material was decomposed into 200 μL of heated HNO_3_ for 30 min and the solution was then diluted by 2% HNO_3_ for ICP-MS detection. The Ce concentrations in the digested samples were quantified by a standard calibration curve (**Figure S2**). The Ce content in the materials could be converted from the measured Ce mass in the digested solution (**Table S1**).

**Oxidase-like activity measurement**

The oxidase-like activity of Ce-MOF nanoagent was assessed by monitoring the catalytic generation of oxTMB at 652 nm using a UV-vis spectrophotometer. The Ce amounts in each trial were controlled at 15 μg/mL. Typically, 20 µL of Ce-MOF solution, 500 µL water and 100 µL of prepared TMB solution were mixed. The catalytic generation of oxTMB could be monitored at 652 nm by a UV-vis spectrophotometer using a time-scanning mode.

**Computational method**

All the calculations were carried out using Density functional theory (DFT) with the Perdew-Burke-Ernzerhof (PBE) functional as implemented in the VASP code.^[13-15]^ The ionic cores were described by the projector-augmented wave (PAW) method^[16,17]^. The cut-off energy for plane wave expansion was set to 400 eV. The electronic self-consistent-loop criterion was set to 10^-8^ eV. During geometry optimization, the structures were relaxed to forces on all the movable atoms smaller than 0.02 eV/Å. A Gaussian smearing method was employed with 0.05 eV width. Grimme’s DFT-D3 methodology^[18]^ was used to describe the dispersion interactions. The Brillouin zone integration was performed using 2×2×2 Monkhorst Pack k-point sampling for all the structures.

The charge difference was computed through the following equation 5:

ρ_diff_ = ρ_AB_ – ρ_A_ – ρ_B_ (5)

Where ρ_AB_ is the electron distribution of the overall composite system, ρ_A_ is that of the structure without the absorbents, ρ_B_ is that of the absorbents (O_2_ or H_2_O).

During optimization of O_2_ and H_2_O adsorption, the substrate atoms were fixed to allow a time-efficient relaxation of the adsorbed species. The adsorption energies (E_ads_) were calculated as follow (Equation 6):

E_ads_ = E_ad/sub_ - E_ad_ - E_sub_ (6)

Where E_ad/sub_, E_ad_ and E_sub_ are the total energies of the optimized adsorbate/substrate system, the adsorbate in the structure and the clean substrate, respectively.

**SEM characterization for bacterial samples**

Firstly, a single colony of bacteria on a solid Luria-Bertani (LB) agar plate was transferred to 10 mL of liquid LB culture medium and was grown at 37 ^o^C for 10-12 hours. Bacteria were harvested by centrifuging (7300 rpm for 3 min), and then were washed with deionized water by twice. The supernatant was discarded and the remaining bacteria were resuspended in water, and diluted to an optical density of 1.0 at 600 nm (OD_600_ = 1.0). Afterwards, bacteria solutions (OD_600_ = 1.0) were incubated with different Ce-MOFs at 37 ^o^C for 4 hours. The bacterial suspension treated by Ce-MOFs were washed with water, and collected by centrifugation at 7300 rpm for 3 min. The collected bacteria were resuspended into 100 μL of water. Then, 2-3 µL of bacterial suspension was dropped onto the clean silicon slice, and dried in air naturally. After that, 2.5% glutaraldehyde solution was added to fix the bacteria overnight. Next, the specimens were washed twice with sterile and dehydrated using a gradient of ethanol solutions (20%, 40%, 60%, 80%, and 100%, each for 5 min). Another group of untreated bacteria was used as a control group. Finally, all samples were sputter-coated with Au for SEM observation.

**AFM Analysis**

The adhesion forces between Ce-MOFs and bacteria were measured by a triangular-shaped standard AFM cantilever (MLCT-O10, Bruker), which was modified with 3-μm-radius silica beads.^[19]^ The silica beads were washed with ultrapure water and then glued on triangular-shaped tipless cantilevers (**Figure S37a)** using UV-curable glue.^[20]^ After 30 min of ultraviolet light exposure, the AFM cantilevers with silica beads were cleaned with ultrapure water. To immobilize bacteria on the poly-L-Lysine functionalized AFM cantilevers, the cleaned AFM probe was subsequently submerged in poly-L-Lysine solution (0.1% (w/v) in H_2_O) and bacterial suspension (OD_600_ = 1.0) for 1 min, respectively.^[21]^ The Ce-MOFs powders were adhered to the glass sheet by double-sided adhesive to create the Ce-MOFs-coated substrates. All force curves were measured in water contact mode. The force curves were drawn at a scanning rate of 0.5 Hz. The trigger threshold and contact time were set to 2 nN and 2 s, respectively.

**The interaction between MD-Ce-UiO-66 and bacteria**

SYTO9-labeled bacteria were obtained by adding SYTO9 staining (50 μL, 0.2 mM) into bacterial suspensions (1mL, OD_600_=1). After 1 h staining, the suspensions were centrifuged (7300 rpm, 3 min), resuspended to obtain the SYTO9-labeled bacteria. The RhB labeled MD-Ce-UiO-66 (RhB-MD-Ce-UiO-66) were prepared by mixing 1 mL MD-Ce-UiO-66 dispersion (1 mg/mL) with 10 μL RhB solution (in DMSO, 1 mg/mL) for 1 h. The mixture was centrifuged (5000 rpm, 3 min) and redispersed. The fluorescence co-localization was conducted by adding the SYTO9-labeled bacteria into RhB-MD-Ce-UiO-66 with CLSM directly.

**Antibacterial experiments**

Firstly, A single colony of bacteria on a solid Luria-Bertani (LB) agar plate was transferred to 10 mL of liquid LB culture medium and was grown at 37^o^ C for 10-12 hours. Bacteria were harvested by centrifuging (7300 rpm for 3 min), and then were washed with deionized water by twice. The supernatant was discarded and the remaining bacteria were resuspended in water, and diluted to an optical density of 1.0 at 600 nm (OD_600_ = 1.0). Afterwards, bacteria solutions (OD_600_ = 1.0) were incubated with different Ce-MOF materials at 37 ^o^C for 4 hours. And then all of the bacteria suspensions were serially diluted 1×10^4^ fold with water. A 100 μL portion of the dilution with bacteria was spread on the solid LB agar plate, and the colonies formed after 12 h incubation at 37 ^o^C were counted. The number of colony-forming units (CFU) was recorded to evaluate the antibacterial performance. The inhibition ratio (IR) was calculated based on the following equation (Equation 7):

$$\begin{aligned} IR=\frac{C_{0}-C}{C_{0}}\times100\%\#\left( 7 \right) \end{aligned}$$

Where C is the CFU of the experimental group treated with the sample solution, and C_0_ is the CFU of the control group without any treatment.

**Bacterial dead/live viability assays**

The bacterial suspension treated by MD-Ce-UiO-66 were washed with water, and collected by centrifugation at 7300 rpm for 3 min. The collected bacteria were resuspended into 100 μL water, and were stained by the mixture of SYTO 9 (50 μL, 0.2 mM) and propidium iodide (PI) (50 μL, 0.2 mM) fluorescent dyes in the dark at 37 ^o^C for 30 min. After washing with water, the samples (10 µL) were dropped onto the slide for fixation. The fluorescence images of SYTO9 and PI were measured in the ranges of 500–540 nm (λ_ex_: 488 nm) and 570–650 nm (λ_ex_: 488 nm), respectively.

**General procedure for MD-Ce-UiO-66 coating on fibrous membrane**

An ethanol solution of the MD-Ce-UiO-66 (5 mg in 1 mL) was sprayed on the square fibrous membrane (3×3 cm^2^) for 20 s and put in an oven under 80 ^o^C for 5 min. The procedure was repeated many times until the ethanol solution in sprayer was empty.

**Antibacterial assays of KN95 mask and** **MD-Ce-UiO-66 mask**

The first layer of the KN95 mask was cut, the fibrous membrane we prepared (Fabric or Fabric/MD-Ce-UiO-66) as the second layer was gently placed between the first layer and the third layer of the KN95 mask, and it was clamped with a clip. *E. coli* was used as a representative for bacteria, and *E. coli*-laden aerosol generated with an aerosol generator was used as a model for bacterial-laden aerosols. Briefly, 20 mL *E. coli* bacterial suspension (OD_600_=1) was added into the tank of an aerosol generator (Qingdao Xincheng Environmental Protection Equipment Co, Ltd., TC-FK), to generate the *E. coli*-laden aerosol upon spraying. The outermost surfaces of KN95 mask and MD-Ce-UiO-66 mask was sprayed with an *E. coli*-laden aerosol (at a flow rate of 0.3 mL/min for 5 min), followed by incubation at 37 ºC for 4 hours. After that, each layer of KN95 mask and MD-Ce-UiO-66 mask was soaked into 20 mL sterile water and then vortexed with a lab dancer for ~1 min. The resulting supernatant was then subjected to serial 250-fold dilutions with sterile water, and 100 µL resultant dilutions were plated onto LB agar plates and incubated at 37 ºC overnight for residual analysis of adhered visible colonies. The reported results are the averages of three independent trials.

**RNAseq analysis**

Fastp software was used to trim the adapters before further processing. Genes were annotated according to GTF files from Ensembl database. The RNAseq reads were mapped onto the Escherichia coli k12 substr mg1655 reference genome by using STAR software. In order to quantify the relative abundance of genes, the RSEM was used to get the normalized metric TPM (million transcripts per kilobase). Genes with expression fold change >= 2 and adjusted p-value <= 0.01 form DEseq2 results were considered differentially expressed. The R package “cluster Profiler” was used for enrichment analysis.

**The cost analysis of** **MD-Ce-UiO-66 mask**

According to the typical synthesis process of MD-Ce-UiO-66, the total price of raw materials required is $0.3 (The unit prices and dosages are shown in **Table S4**), and it affords MD-Ce-UiO-66 with a mass of about 100 mg each time. In the general procedure of MD-Ce-UiO-66 coating on fibrous membrane, 5 mg MD-Ce-UiO-66 is sprayed onto the 3×3 cm^2^ square fibrous membrane. The MD-Ce-UiO-66 dosage (D_a_) of the whole middle layer of the mask is calculated based on the following equation (Equation 8):

$$\begin{aligned} D_{a}=\frac{D_{b}\times M_{a}}{M_{b}}\#\left( 8 \right) \end{aligned}$$

Where: D_b_ (5 mg) is the MD-Ce-UiO-66 dosage of the 3×3 cm^2^ square fibrous membrane. M_a_ (2878.1 mg) represents the mass of the entire middle layer of the mask. M_b_ (108.4 mg) represents the mass of 3×3 cm^2^ square fibrous membrane.

With D_b_, M_a_ and M_b_ known, we can use Equation (8) to calculate the MD-Ce-UiO-66 dosage of the entire middle layer of the mask, D_a_:

$$D_{a}=\frac{5\times2878.1}{108.4}$$

$$D_{a}=132.8$$

As discussed above, it costs $0.3 to produce 100 mg of MD-Ce-UiO-66, and 132.8 mg of MD-Ce-UiO-66 is required for spraying onto the entire middle layer of the mask. Thus, the corresponding raw material cost for the entire middle layer of the mask is estimated to be $0.4.

An ordinary KN95 mask currently costs about $0.03, therefore, the costs of our MD-Ce-UiO-66 mask is estimated to be ca $0.43 per one.

**Tables**

**Table S1.** The Ce contents in different nanoagents based on ICP-MS

| Sample | Chemical  formula | Theoretical Ce content (%) | |  | Measured Ce content (%) | |  |
| --- | --- | --- | --- | --- | --- | --- | --- |
| Ce-BTB-MOL | Ce_6_O_4_(OH)_4_(BTB)_2_(OH)_6_(H_2_O)_6_ |  | 40.94 | | | 29.10 | |
| Ce-PCN-224 | Ce_6_O_4_(OH)_4_(BA)_6_(H_2_TCPP)_1.5_ |  | 29.20 | | | 25.06 | |
| Ce-NU-1000 | Ce_6_O_4_(OH)_4_(BA)_4_(TBAPy)_2_ |  | 29.87 | | | 24.4 | |
| Ce-MOF-808 | Ce_6_O_4_(OH)_4_(BTC)_2_(OH)_6_(H_2_O)_6_ |  | 52.64 | | | 46.32 | |
| Ce-UiO-66 | Ce_6_O_4_(OH)_4_(BDC)_6_ |  | 41.51 | | | 40.68 | |
| MD-Ce-UiO-66 | Ce_6_O_4_(OH)_4_(BDC)_6_ |  | 41.51 | | | 39.33 | |

**Table S2.** The valence states of Ce in different nanoagents based on XPS spectra.

| Sample | Ce^4+^ (%) | Ce^3+^ (%) | Ce^4+^/Ce^3+^ |
| --- | --- | --- | --- |
| Ce-BTB-MOL | 70.19 | 29.80 | 2.36 |
| Ce-PCN-224 | 68.30 | 31.70 | 2.15 |
| Ce-NU-1000 | 70.34 | 29.66 | 2.37 |
| Ce-MOF-808 | 67.40 | 32.60 | 2.07 |
| Ce-UiO-66 | 62.52 | 37.48 | 1.69 |
| MD-Ce-UiO-66 | 62.63 | 37.37 | 1.68 |

**Table S3.** The valence states of Ce before and after soaking in water for 12 d.

| Sample | Ce^4+^ (%) | Ce^3+^ (%) | Ce^4+^/Ce^3+^ |
| --- | --- | --- | --- |
| MD-Ce-UiO-66 | 62.63 | 37.37 | 1.68 |
| MD-Ce-UiO-66 soaking in water for 12 d | 62.84 | 37.16 | 1.69 |

**Table S4.** The cost of raw materials for the preparation of 100 mg MD-Ce-UiO-66.

| Raw material | Unit price ($/mL or $/g) | Dosage (mL or g) | Price ($) | Total price ($) |
| --- | --- | --- | --- | --- |
| P123 | 0.46 | 0.1 | 0.046 | 0.30 |
| F127 | 0.26 | 0.05 | 0.013 |  |
| Toluene | 0.0098 | 0.24 | 0.0024 |  |
| AA | 0.0012 | 0.1 | 0.00012 |  |
| NaClO_4_·H_2_O | 0.11 | 0.3 | 0.033 |  |
| BDC | 0.13 | 0.12 | 0.016 |  |
| (NH_4_)_2_Ce(NO_3_)_6_ | 0.34 | 0.548 | 0.186 |  |

**Figures**


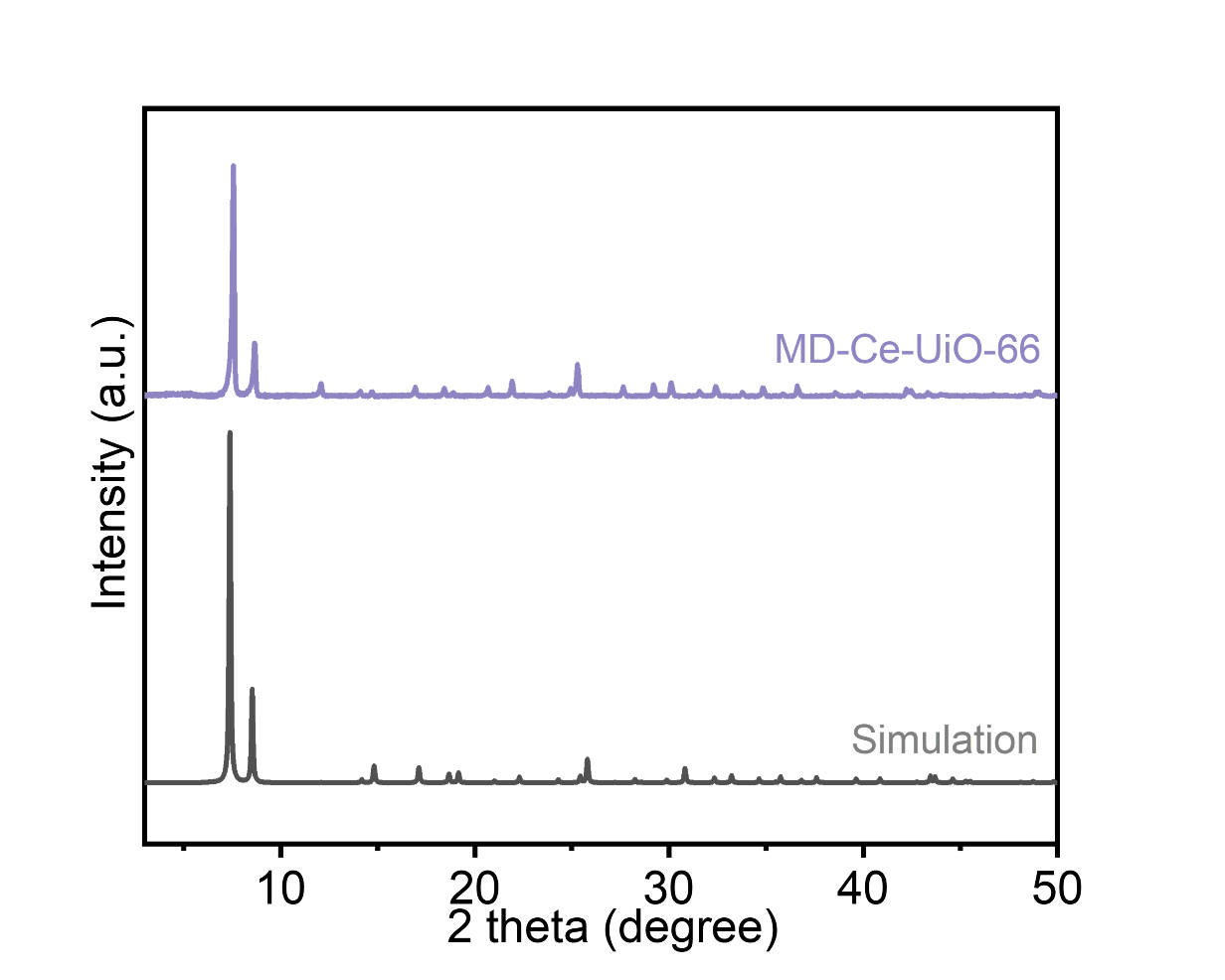


**Figure S1**. The PXRD pattern of MD-Ce-UiO-66.

**
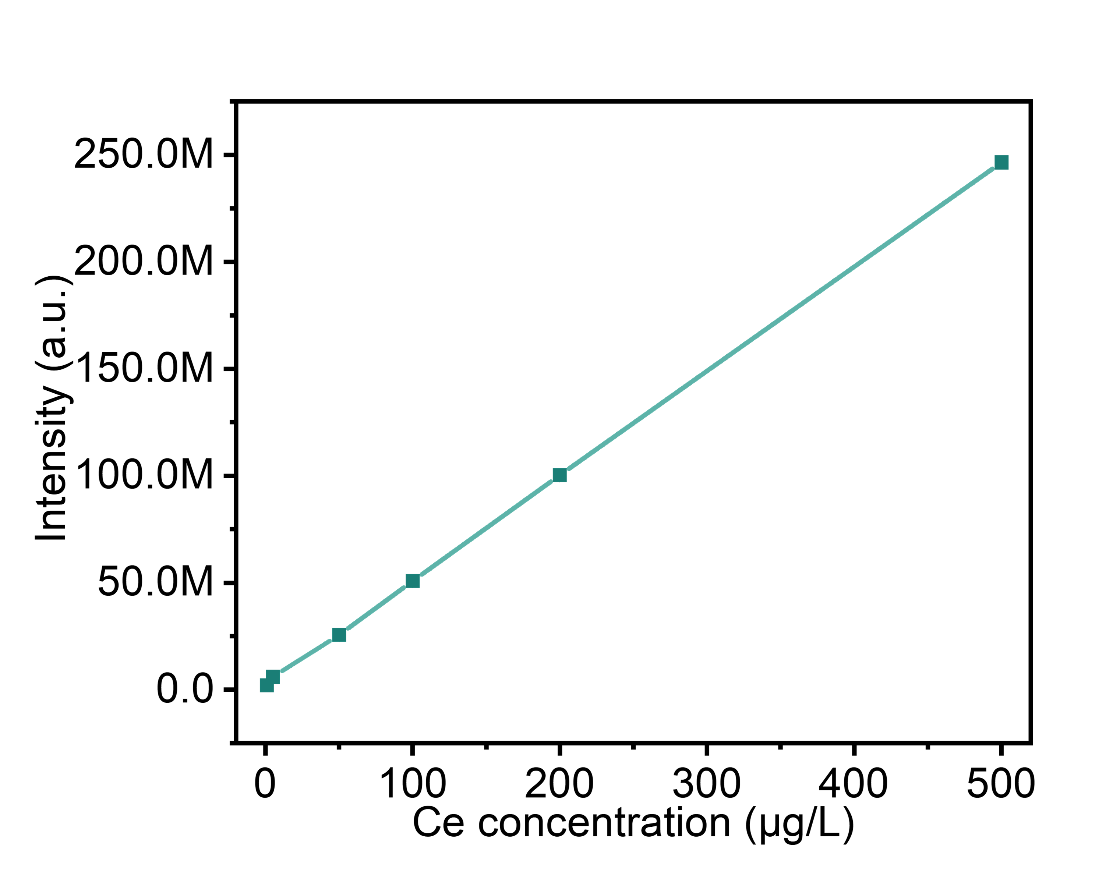
**

**Figure S2**. The calibration curve for Ce quantification using ICP-MS. The error bars are representative of the standard deviation of the triplicates.





**Figure S3**. SEM image of MD-Ce-UiO-66.





**Figure S4**. TEM image of MD-Ce-UiO-66.

**
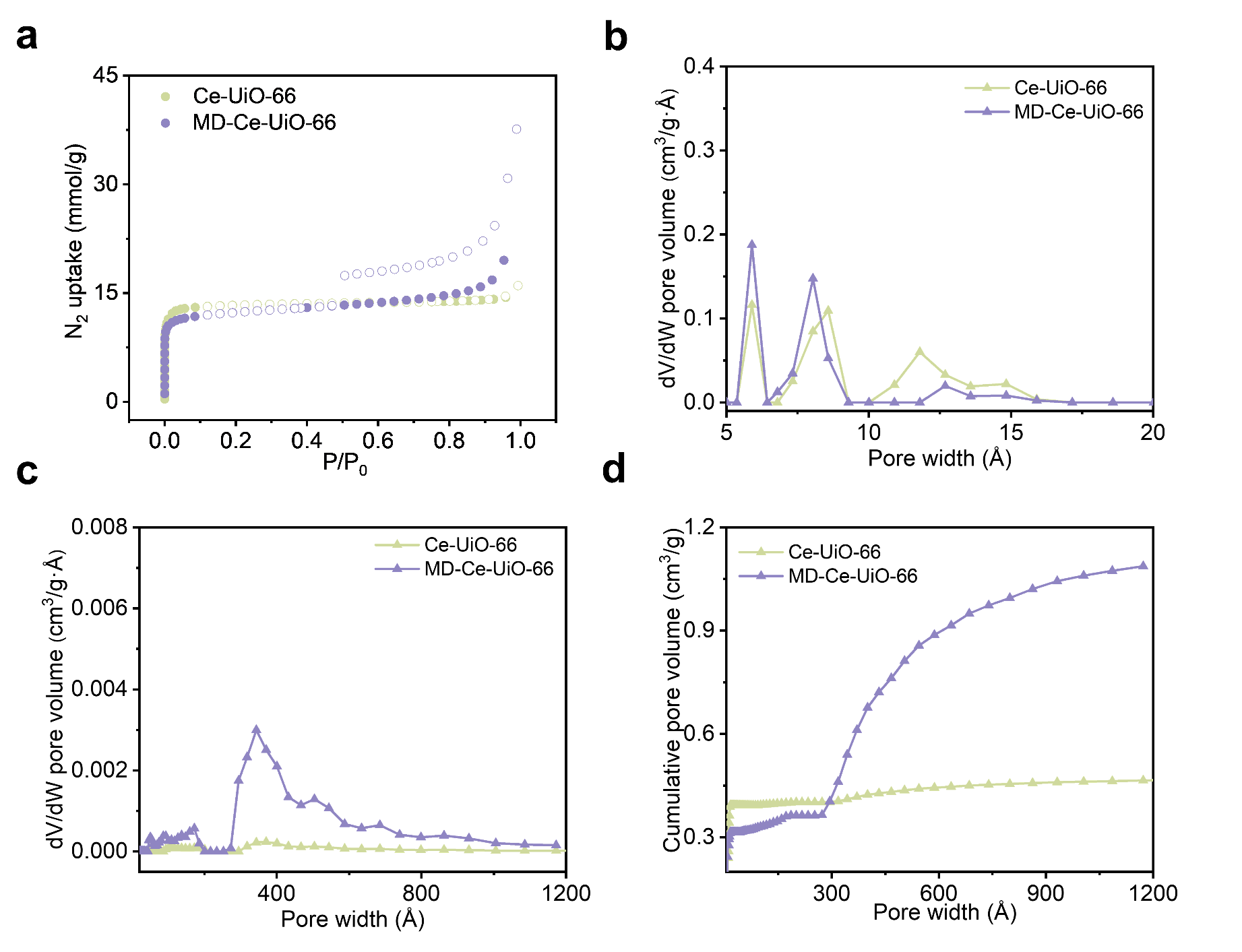
**

**Figure S5**. (a) The N_2_ sorption isotherms of Ce-UiO-66 and MD-Ce-UiO-66. (b-d) The pore-size distributions of Ce-UiO-66 and MD-Ce-UiO-66 based on NLDFT method.

**
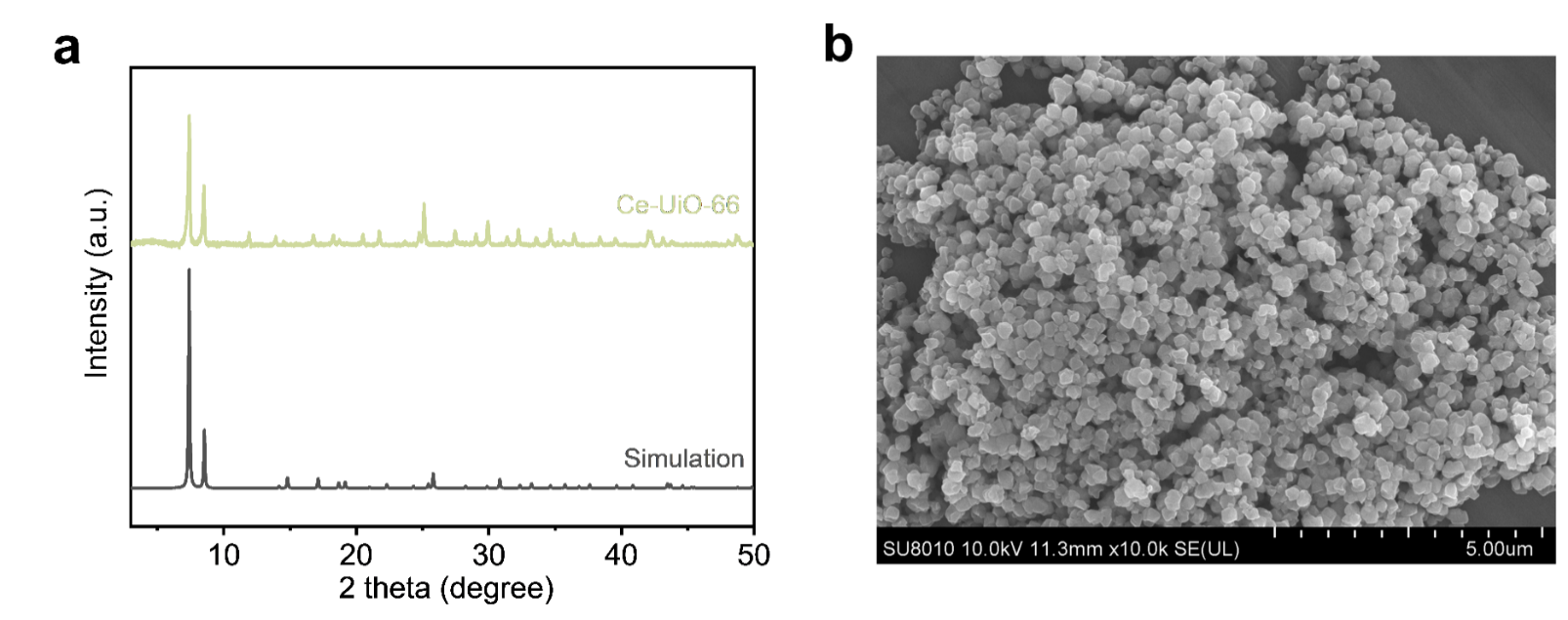
**

**Figure S6**. The PXRD pattern (a) and SEM image (b) of Ce-UiO-66.

**
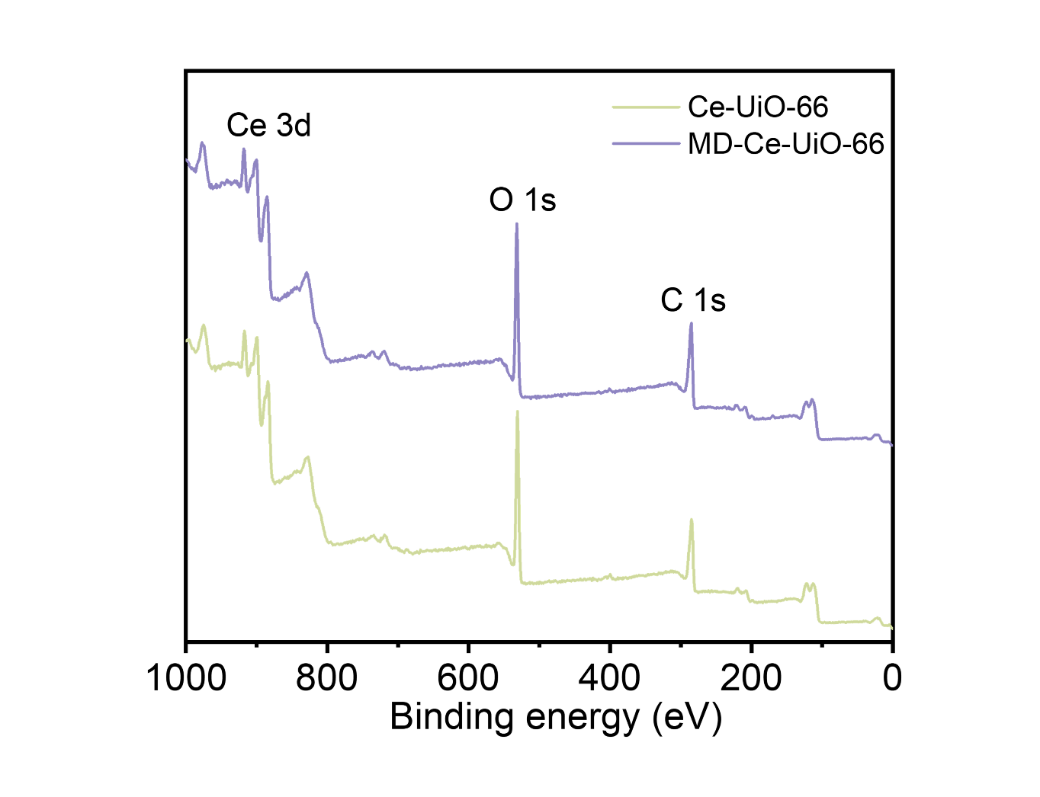
**

**Figure S7**. The XPS wide-scan spectra of Ce-UiO-66 and MD-Ce-UiO-66.


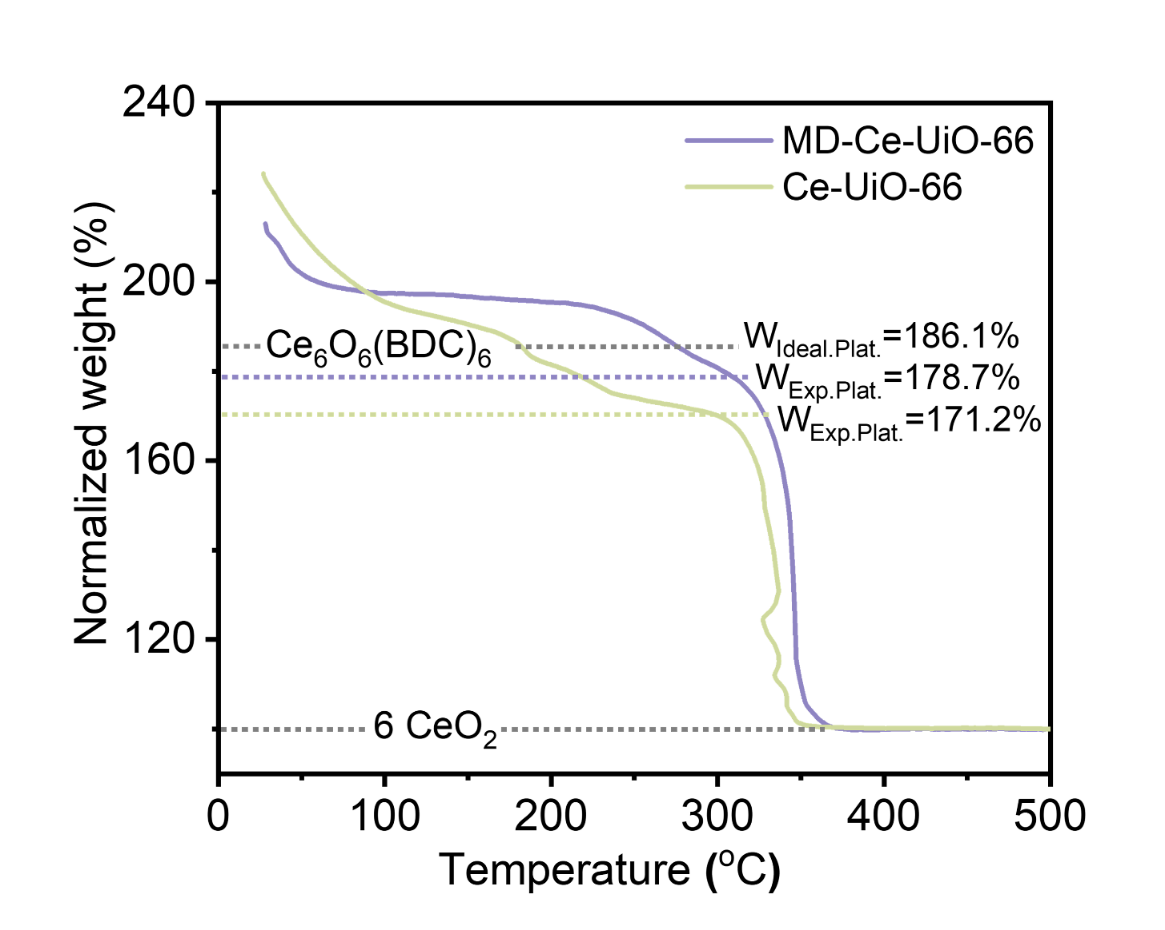


**Figure S8**. TGA analysis of the synthesized MD-Ce-UiO-66 and Ce-UiO-66. Solid curve, left axis -TGA trace (normalized such that end weight = 100%). The horizontal dashed lines pinpoint the relevant TGA plateaus.

**Note**: The selected plateau must represent the state in which the composition of the material is Ce_6_O_6+x_(BDC)_6-x_. That is, when all of solvent, hydroxyl groups and modulator have been eliminated and only the linker remains. In the TGA curves, the experimental plateau weights (W_Exp.Plat._) of the synthesized MD-Ce-UiO-66 and Ce-UiO-66 are determined as 178.7% and 171.2%, respectively. These are much lower than the plateau theoretically expected for an ideal Ce-UiO-66 sample (W_Ideal.Plat._ = 186.1 %). It indicates that our synthesized MD-Ce-UiO-66 and Ce-UiO-66 are linker deficient.

With W_Exp.Plat._ known, we can use Equation (4) to calculate the number of linker deficiencies per Ce_6_ formula unit, x:

$x=6-\mathrm{NL}_{E\mathrm{xp}.}=6-\frac{(W_{Exp.plat.}-W_{\mathrm{End}})}{{Wt.PL}_{Theo.}}$ (4)

For the synthesized MD-Ce-UiO-66:

$$x=6-\mathrm{NL}_{Exp.}=6-\frac{(W_{Exp.plat.}-W_{\mathrm{End}})}{{Wt.PL}_{Theo.}}$$

$$x=6-\frac{(178.7-100\%)}{14.35}$$

$$x=6-5.48$$

$$x=0.52$$

For the synthesized Ce-UiO-66:

$$x=6-\mathrm{NL}_{Exp.}=6-\frac{(W_{Exp.plat.}-W_{\mathrm{End}})}{{Wt.PL}_{Theo.}}$$

$$x=6-\frac{(171.2-100\%)}{14.35}$$

$$x=6-4.96$$

$$x=1.04$$

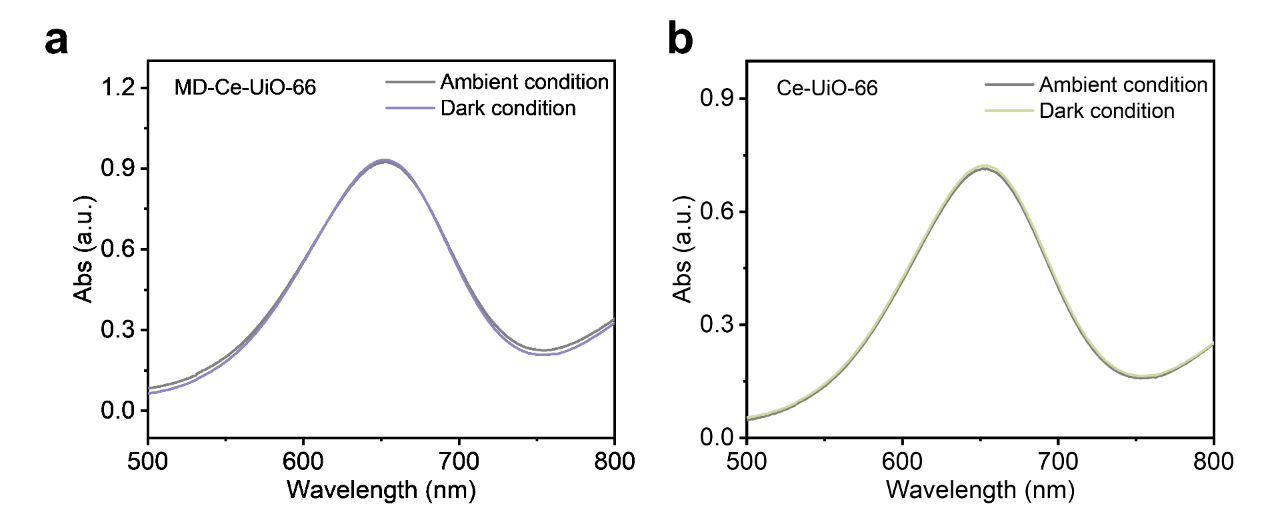


**Figure S9**. Oxidase-like activity of MD-Ce-UiO-66 (a) and Ce-UiO-66 (b) under ambient condition or dark condition. The UV-vis spectra presented the absorbance of the catalytic product after 2 min reaction.


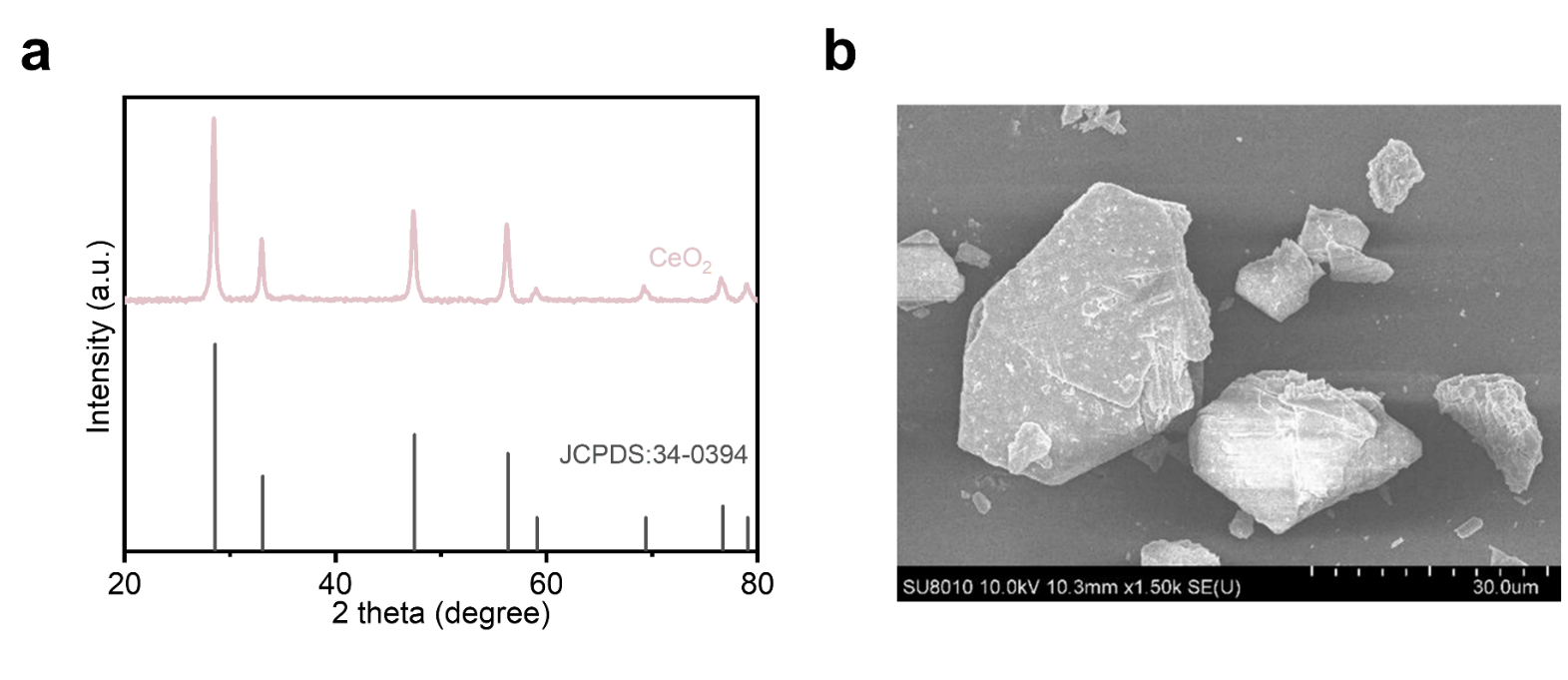


**Figure S10**. The PXRD pattern (a) and SEM image (b) of bulky CeO_2_.

**
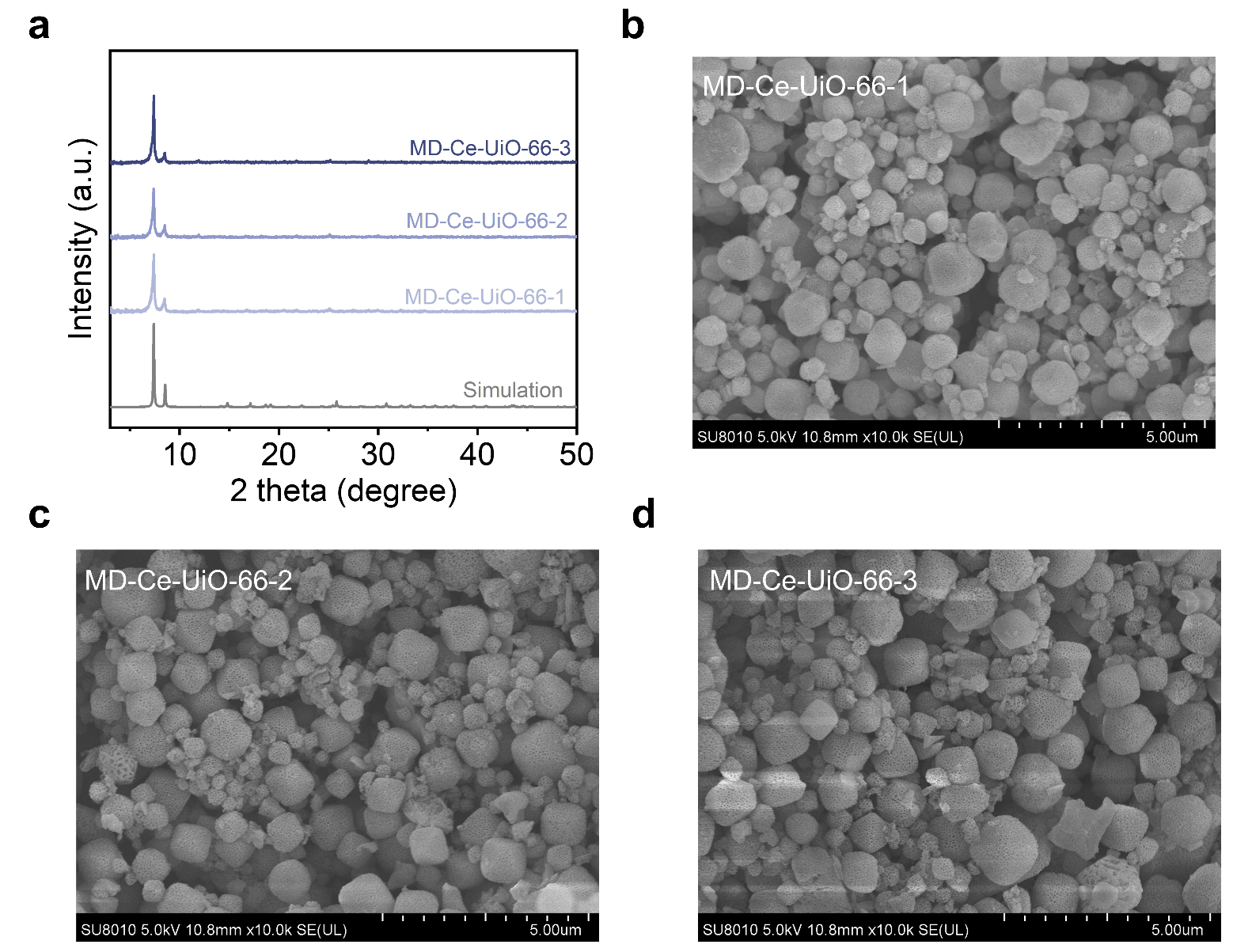
**

**Figure S11**. The PXRD patterns (a) and SEM images (b-d) for three batch samples of MD-Ce-UiO-66.

**
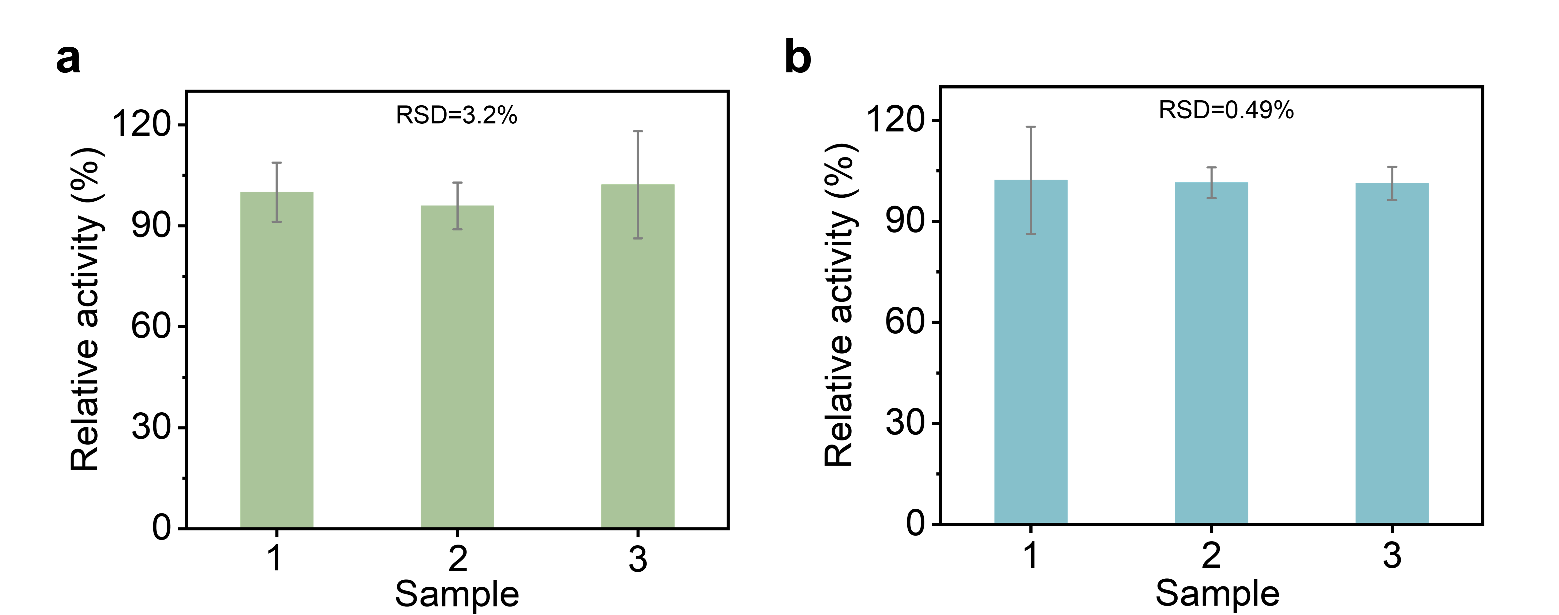
**

**Figure S12.** The variations of oxidase-like activity in the three batch samples of MD-Ce-UiO-66 (a) and in the triplicate samples from the same batch (b)

**Note:** We concurrently prepared three batch samples of MD-Ce-UiO-66 (**Figure S11a**), all of which presented uneven particle size but formed rich mesoporous channels in each particle (**Figures S11b** to **S11d**). Upon assessing the oxidase activity of the three batch samples, we found minimal variation of activity with a low RSD value of 3.2% (**Figure S12a**). Additionally, we also conducted triplicate activity tests on the same batch sample and found that they presented negligible differences of activity (RSD=0.49%, **Figure S12b**). These results indicated that the heterogeneity in particle size of MD-Ce-UiO-66 had a negligible impact on its oxidase activity.


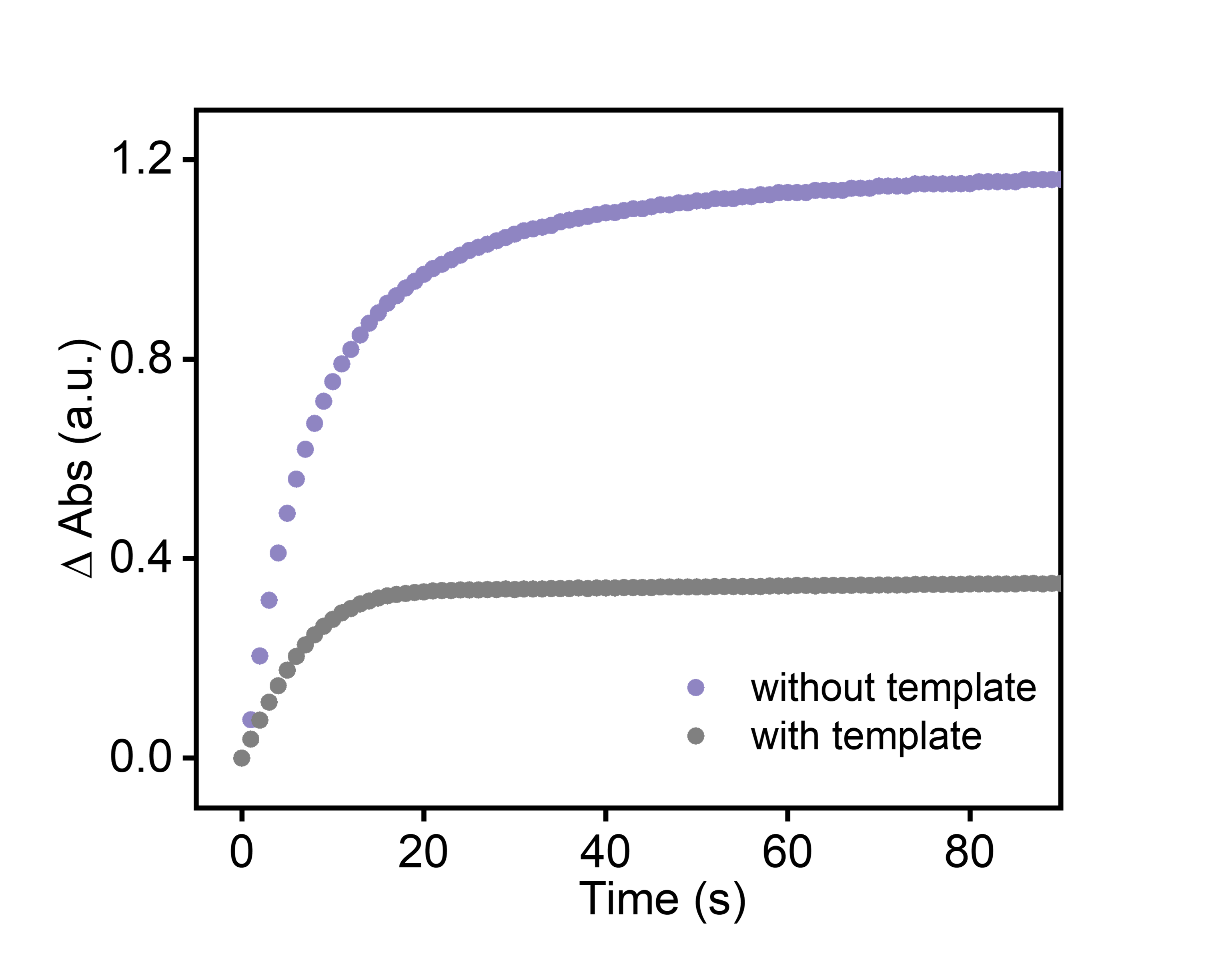


**Figure S13**. The catalytic kinetics curves of MD-Ce-UiO-66 before and after polymer template removal under the same Ce amounts.


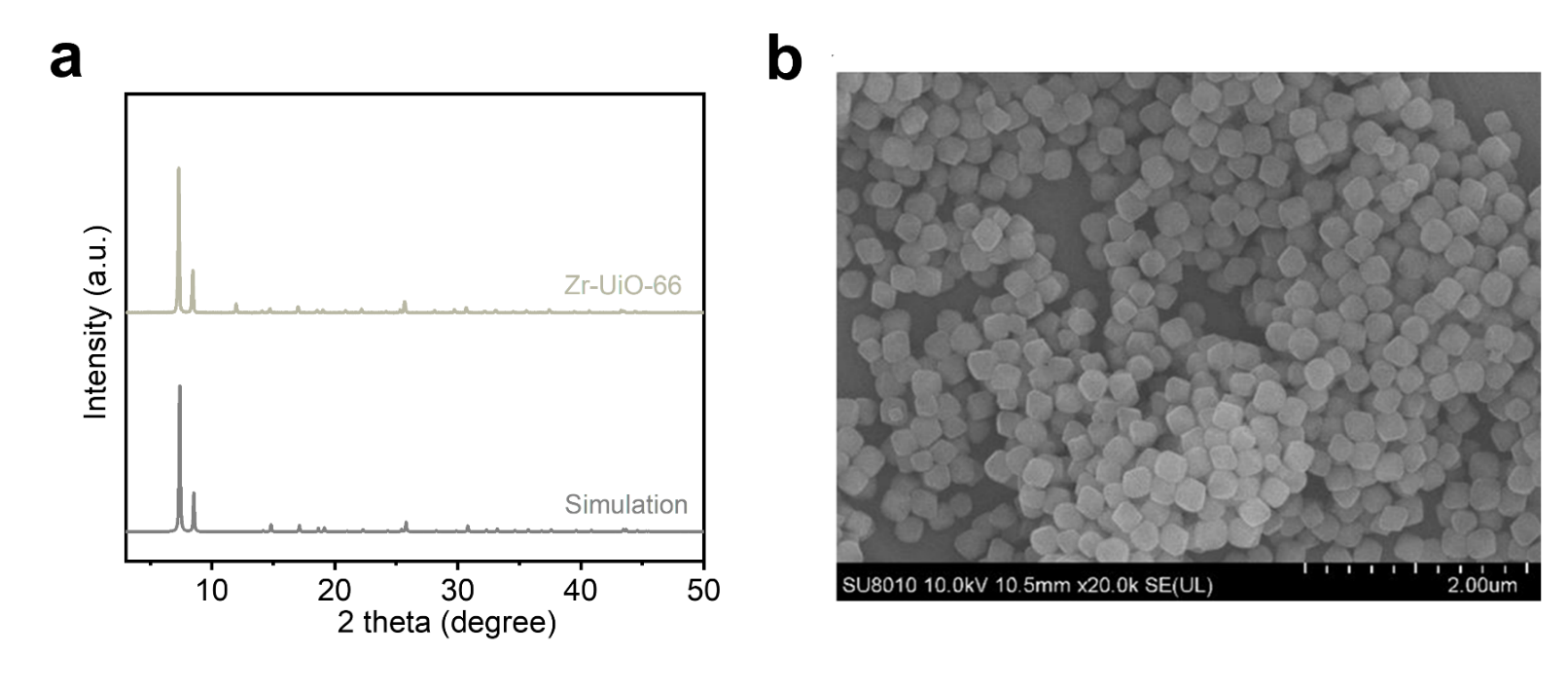


**Figure S14**. The PXRD pattern (a) and SEM image (b) of Zr-UiO-66.


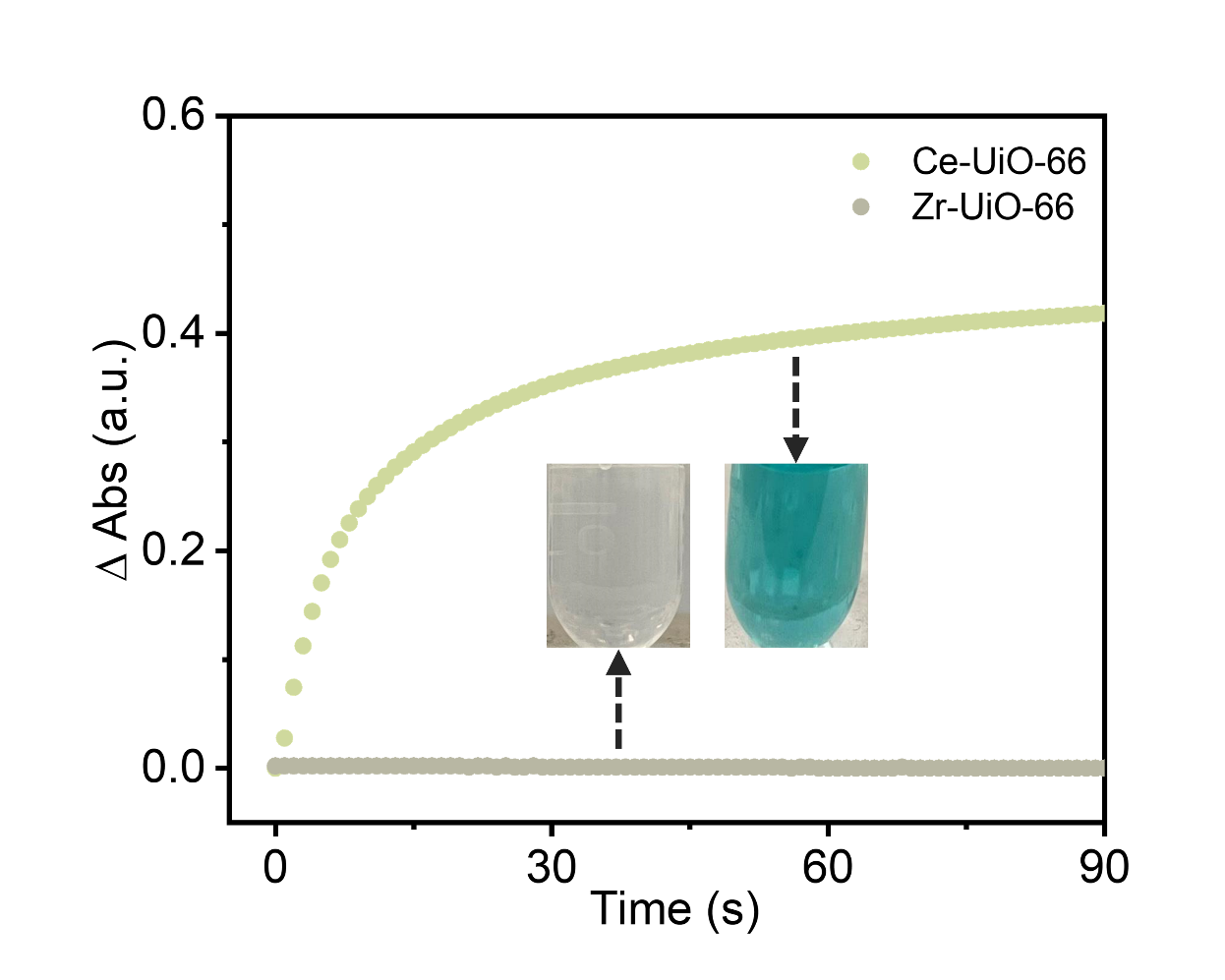


**Figure S15**. The catalytic kinetics curves of Zr-UiO-66 and Ce-UiO-66. Insets were the photographs recording the apparent color change.


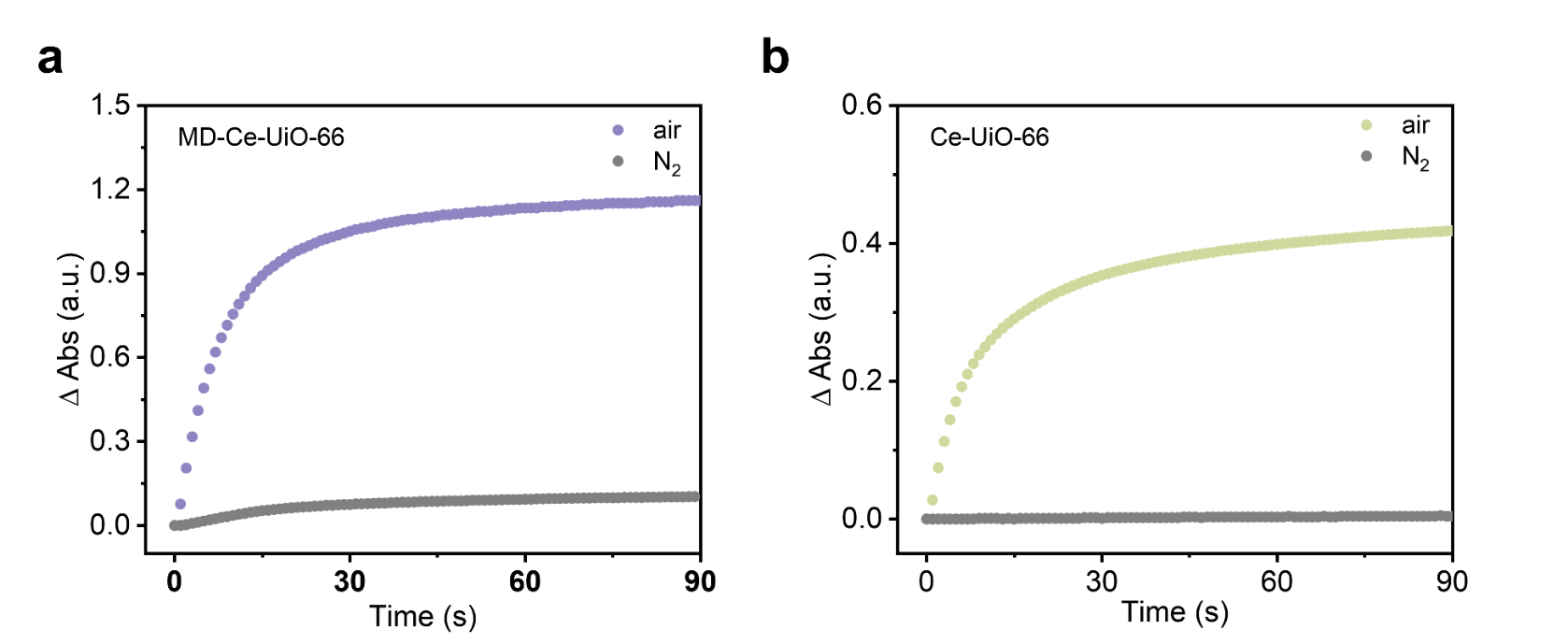


**Figure S16**. The catalytic kinetics curves of MD-Ce-UiO-66 (a) and Ce-UiO-66 (b) under the air or N_2_ atmospheres. The Ce in each group was kept at the same amounts.


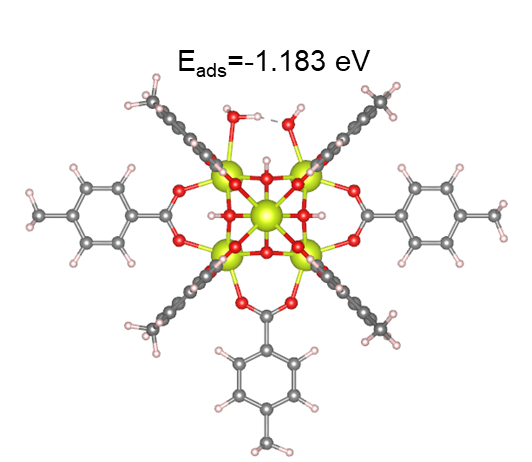


**Figure S17**. The structural model of Ce_6_ cluster with a linker-missing site, onto which one hydroxo and one aqua ligand was ligated. Red ball represented O atom; yellow ball represented Ce atom; gray ball represented C atom; pink ball represented H atom.


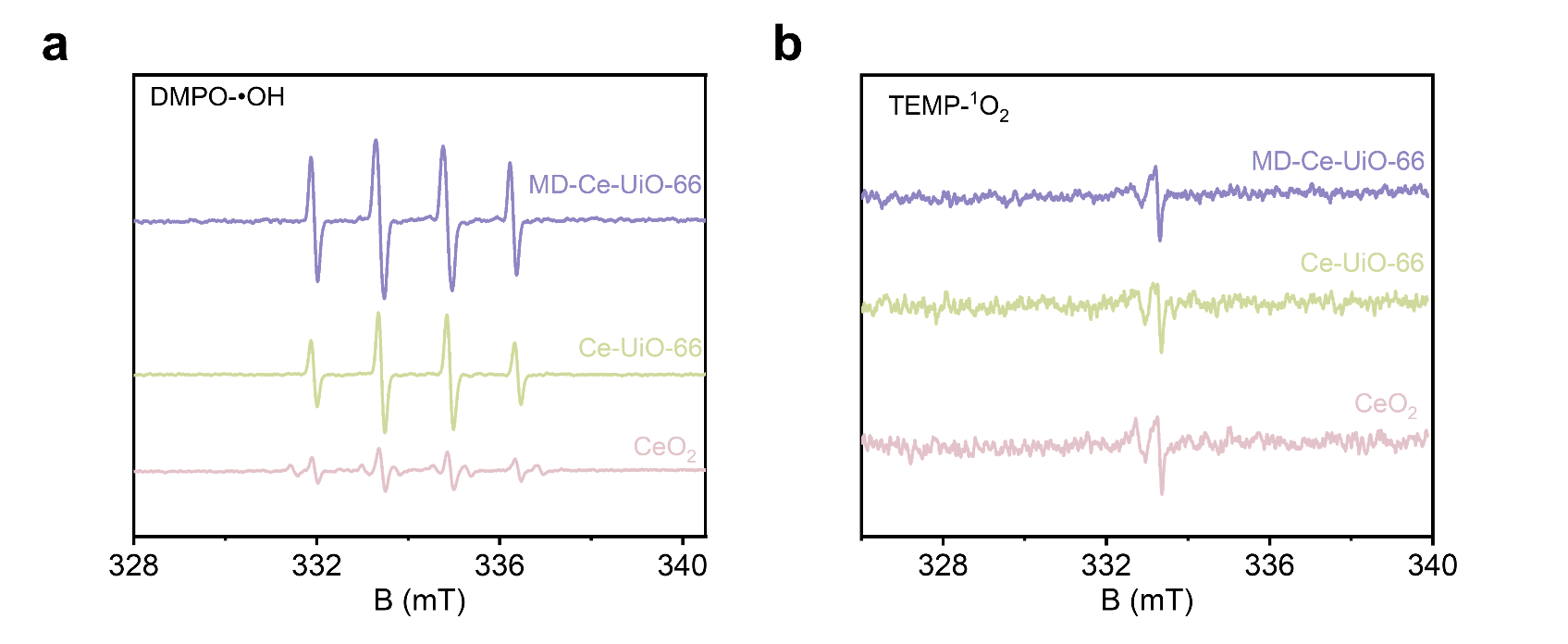


**Figure S18**. (a) The EPR spectra of DMPO-•OH (a) TEMP-^1^O_2_ (b) for CeO_2_, Ce-UiO-66 and MD-Ce-UiO-66.


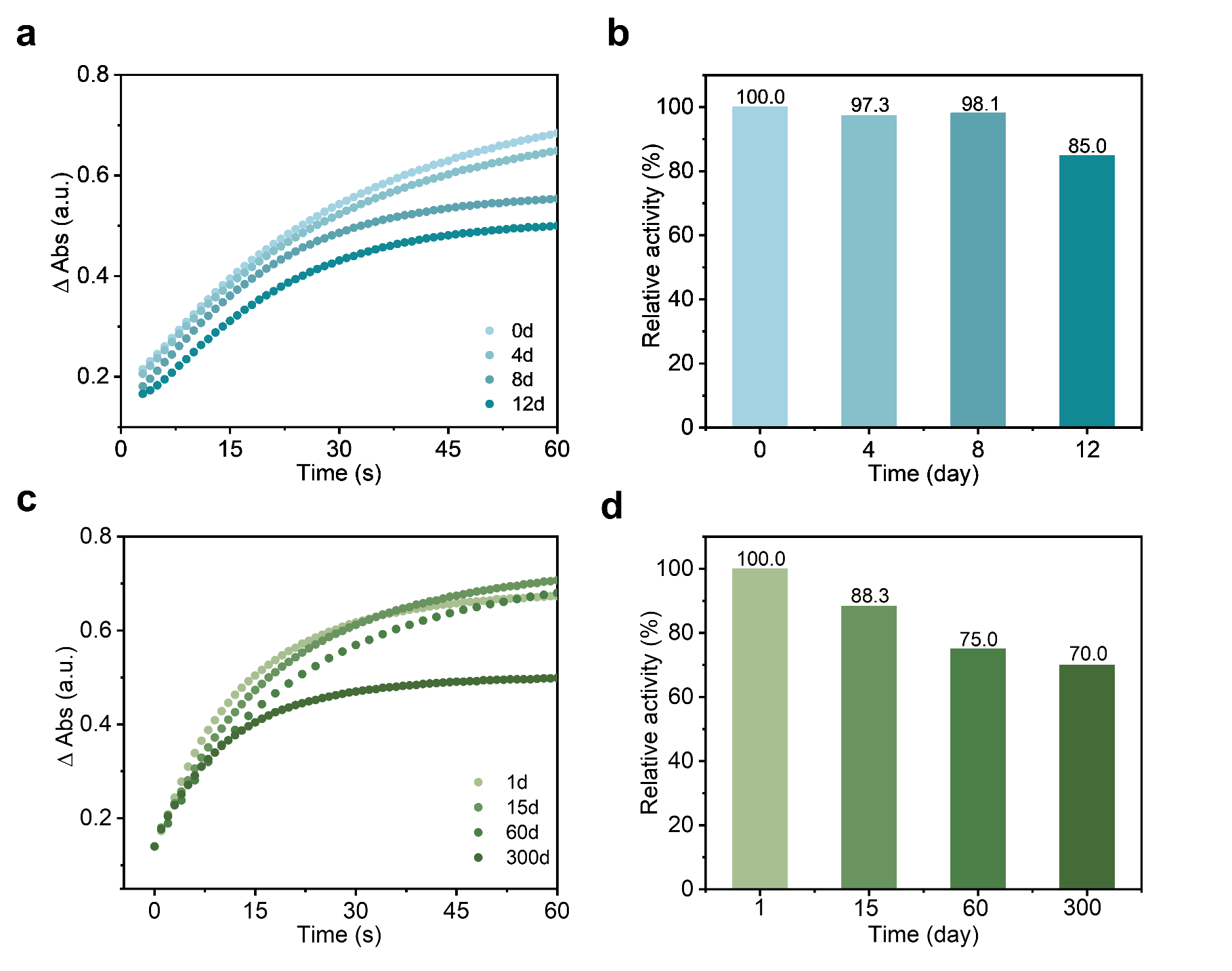


**Figure S19**. The oxidase-like catalytic kinetics curves (a) and reactive activities (b) of MD-Ce-UiO-66 after soaking in water for different days. The catalytic kinetics curves (c) and reactive activities (d) of MD-Ce-UiO-66 after storing it at room temperature for different days. The concentration of MD-Ce-UiO-66 used in these tests was set at 32 μg/mL.


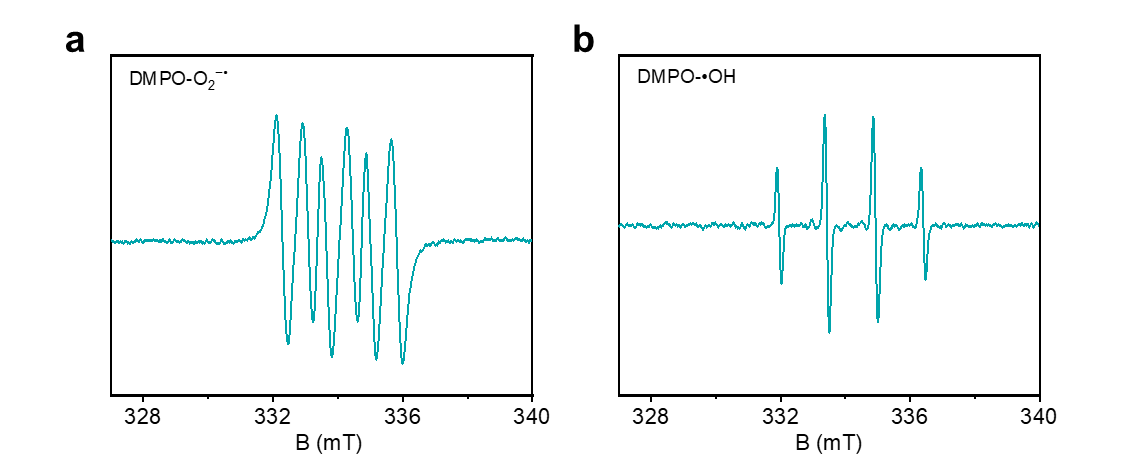


**Figure S20**. The EPR spectra of DMPO-O_2_^−•^ (a) and DMPO-•OH (b) of MD-Ce-UiO-66 sample after soaking it in water for 12 d.

**
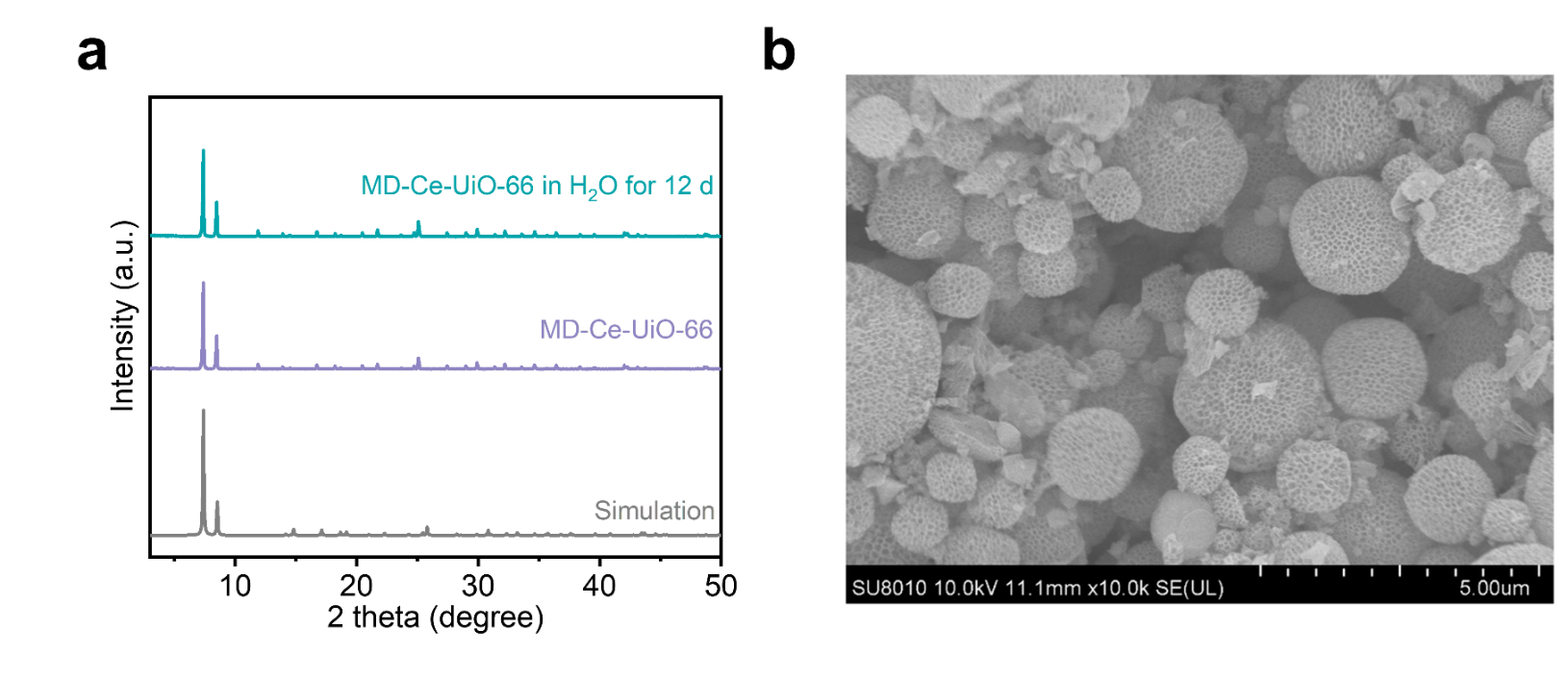
**

**Figure S21**. (a) The PXRD patterns and (b) SEM image of MD-Ce-UiO-66 before and after soaking it in water for 12 d.


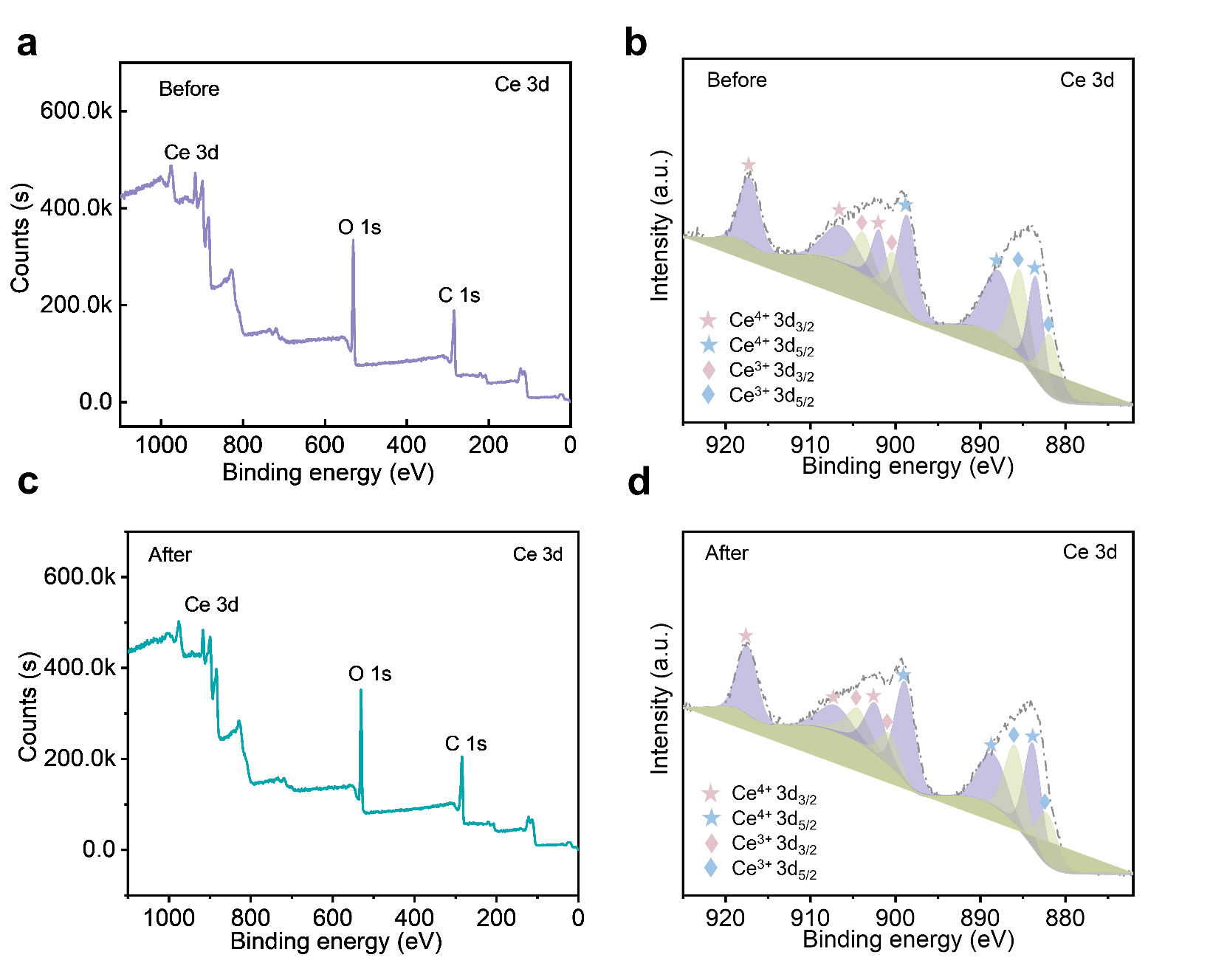


**Figure S22**. The XPS wide-scan spectra (a and c) and Ce 3d XPS spectra (b and d) of MD-Ce-UiO-66 before and after soaking it in water for 12 d.


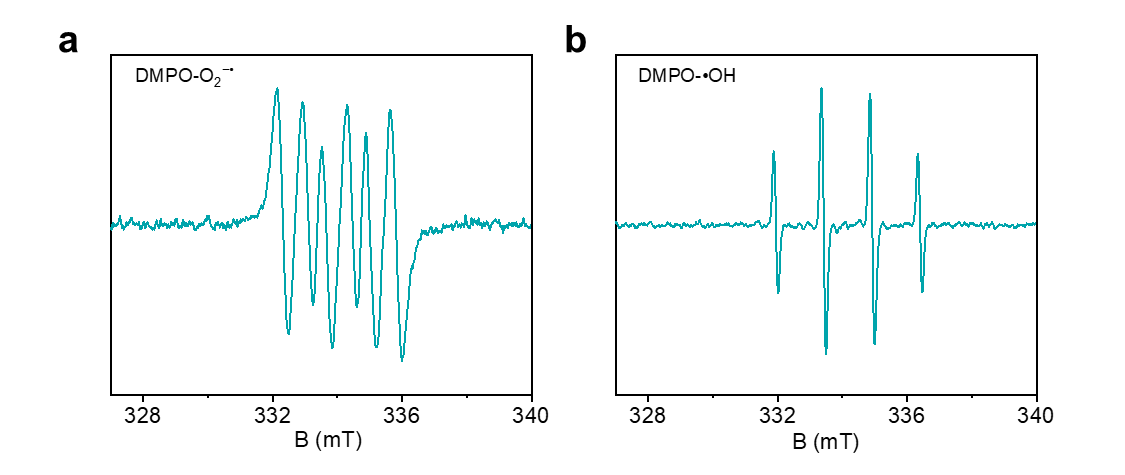


**Figure S23**. The EPR spectra of DMPO-O_2_^−•^ (a) and DMPO-•OH (b) of MD-Ce-UiO-66 sample after storing it at room temperature after 300 d.


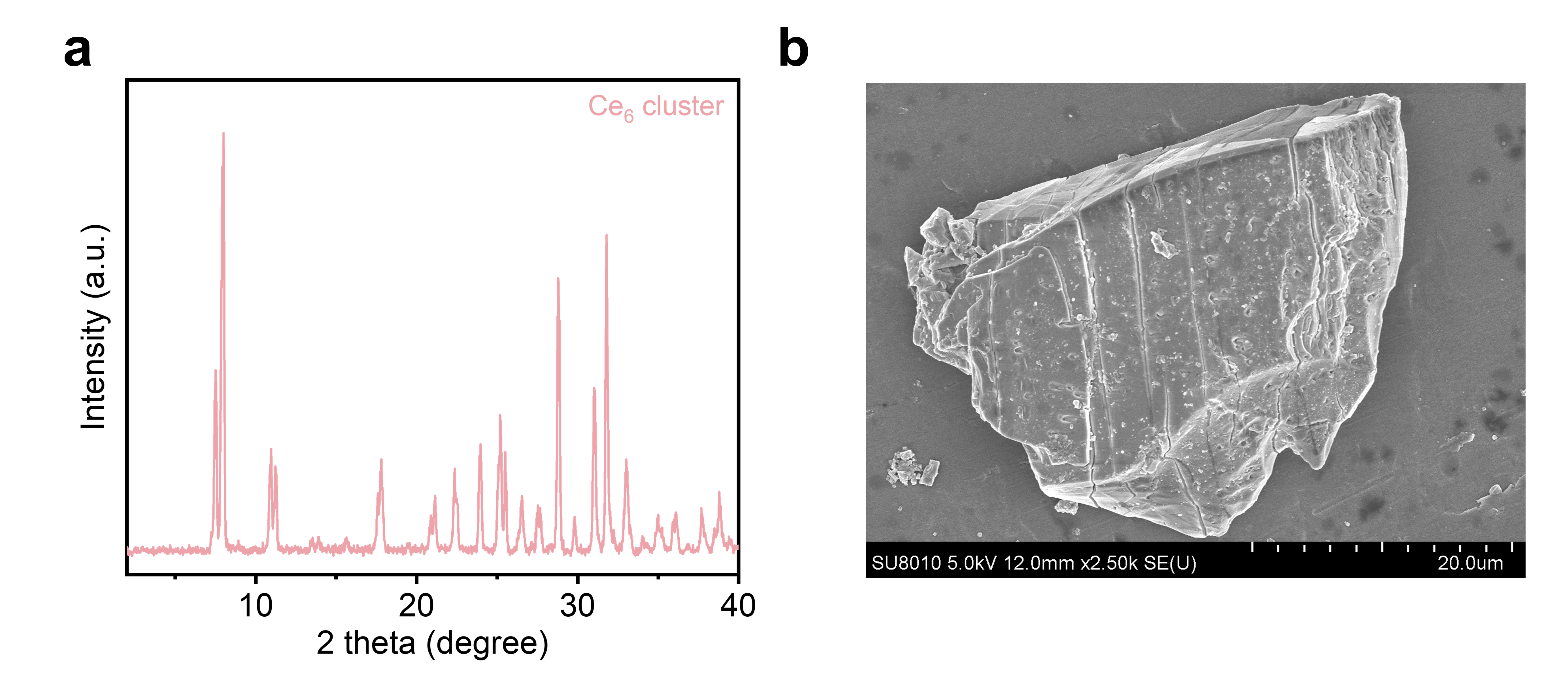


**Figure S24**. The PXRD pattern (a) of and SEM image (b) of Ce_6_ cluster.


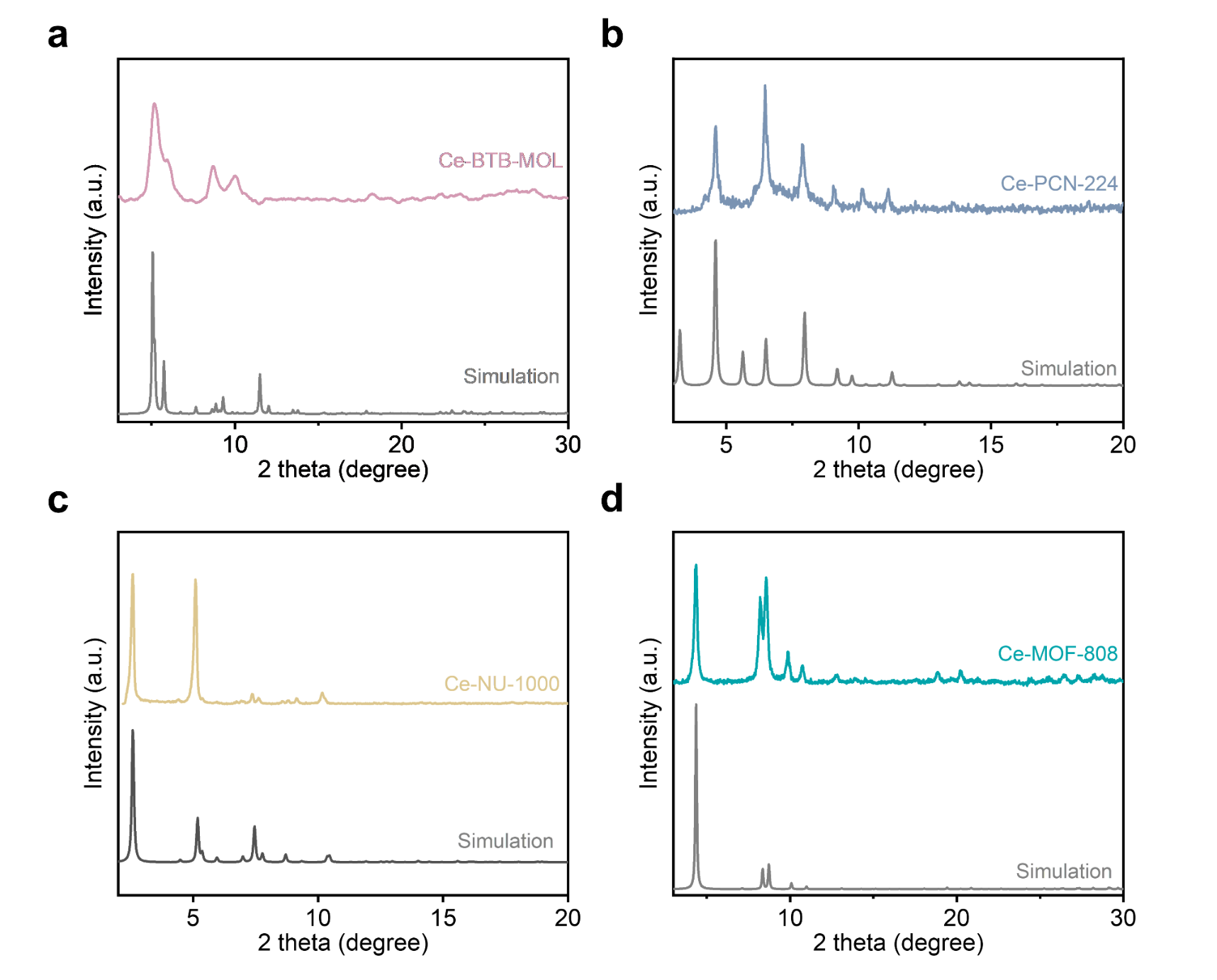


**Figure S25**. The PXRD patterns of Ce-BTB-MOL (a), Ce-PCN-224 (b), Ce-NU-1000 (c) and Ce-MOF-808 (d). All of them showed strong Bragg diffraction peaks, matching well with the corresponding simulated structures.


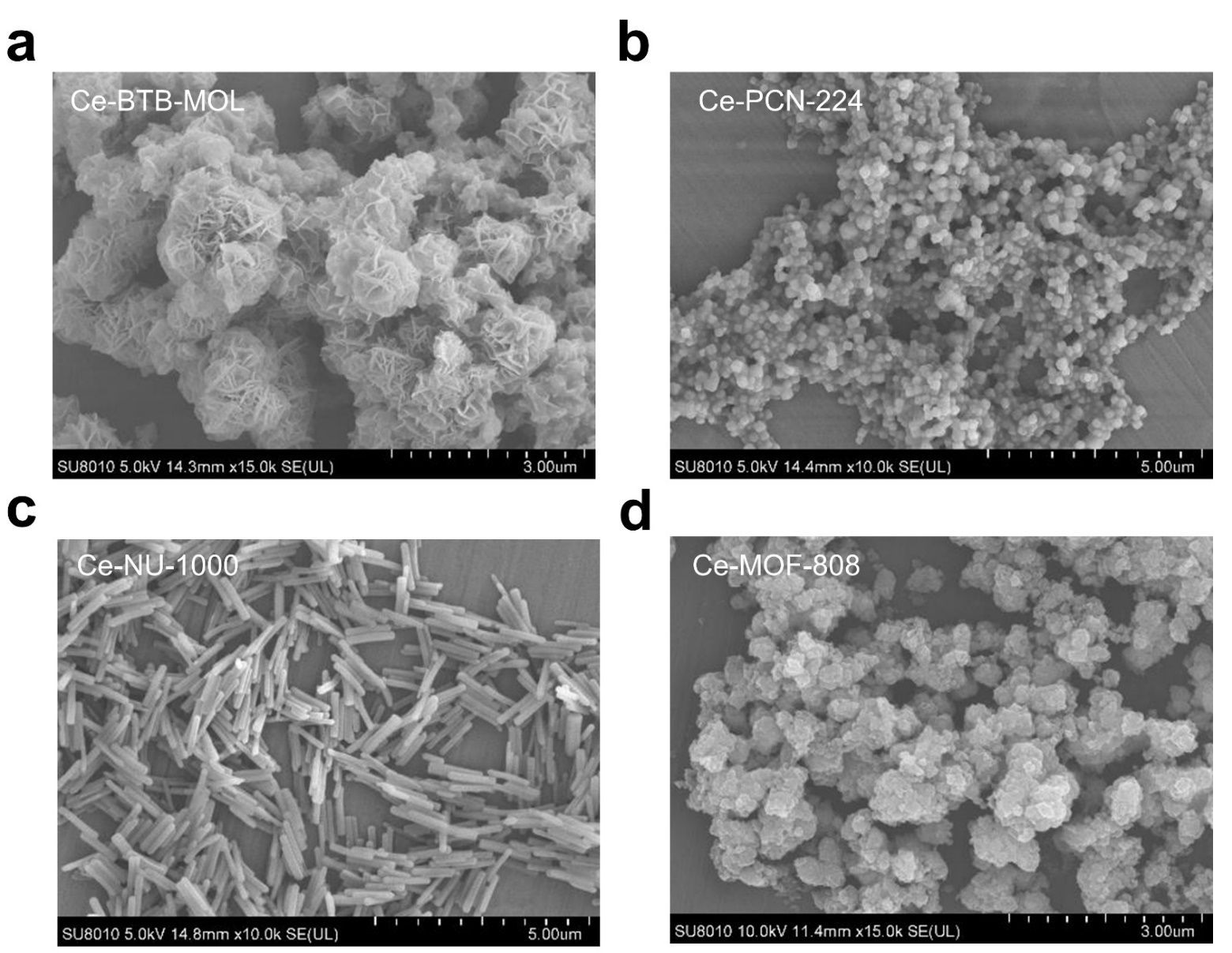


**Figure S26**. The SEM images of Ce-BTB-MOL (a), Ce-PCN-224 (b), Ce-NU-1000 (c) and Ce-MOF-808 (d).


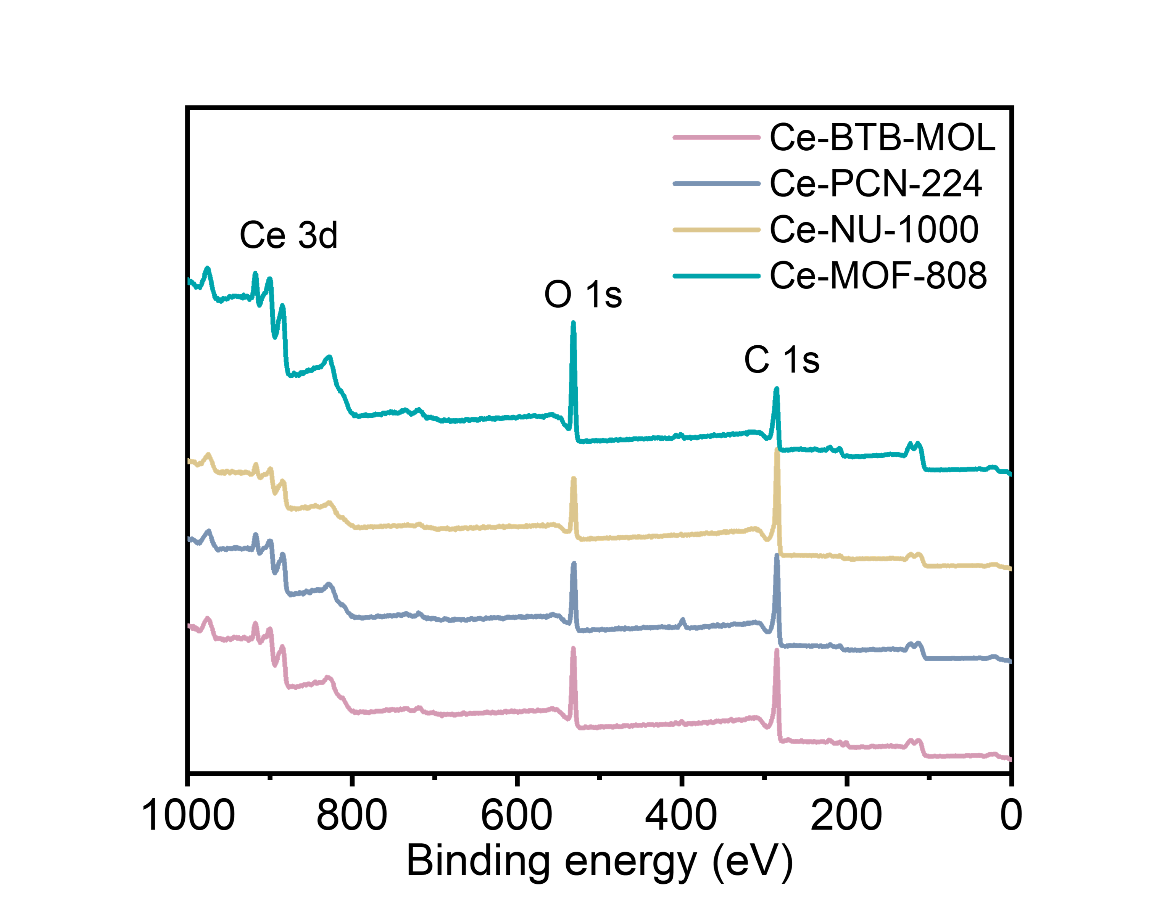


**Figure S27**. XPS wide-scan spectra of Ce-BTB-MOL, Ce-PCN-224, Ce-NU-1000 and Ce-MOF-808.


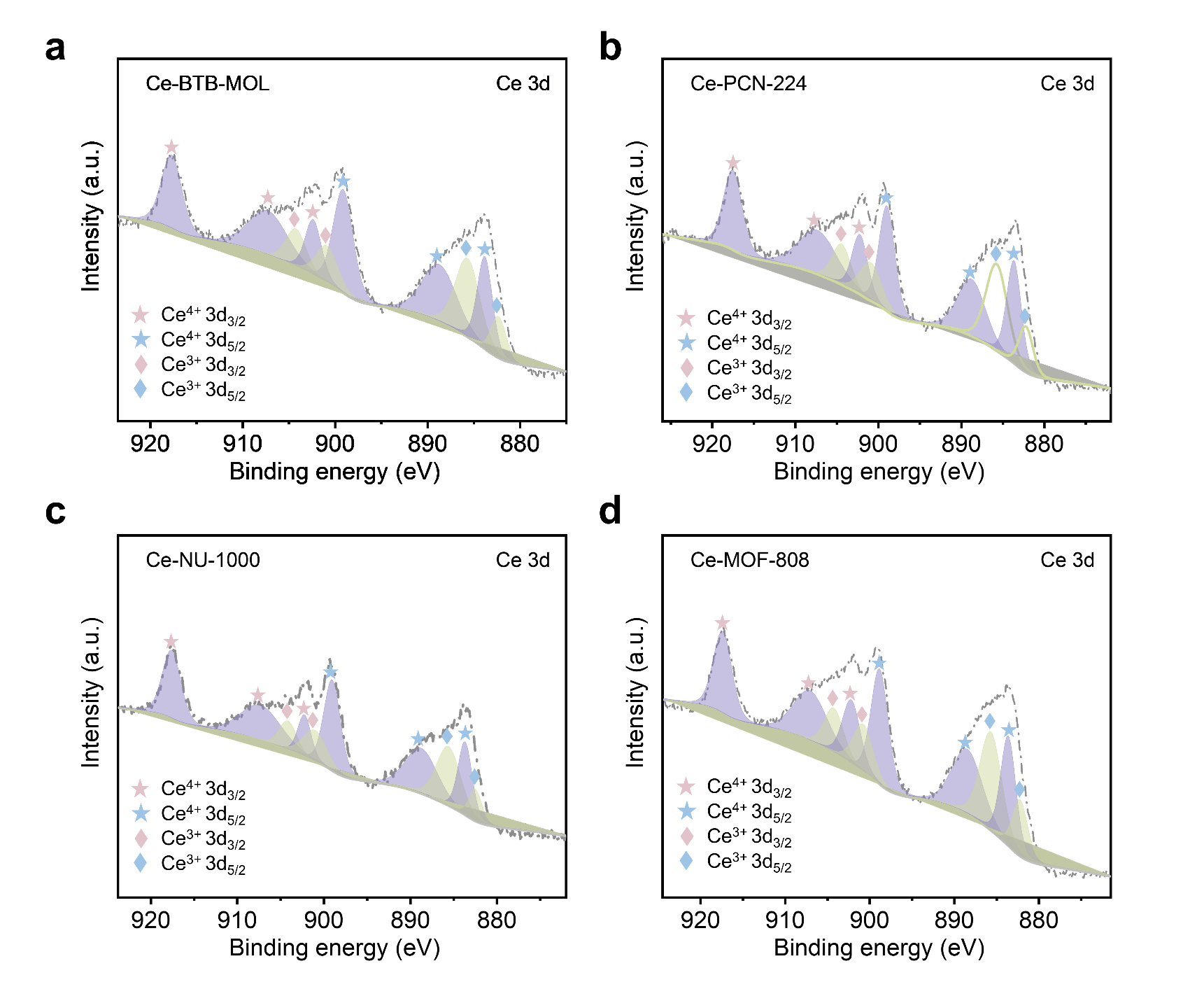


**Figure S28**. The XPS Ce 3d XPS spectra of Ce-BTB-MOL (a), Ce-PCN-224 (b), Ce-NU-1000 (c) and Ce-MOF-808 (d).


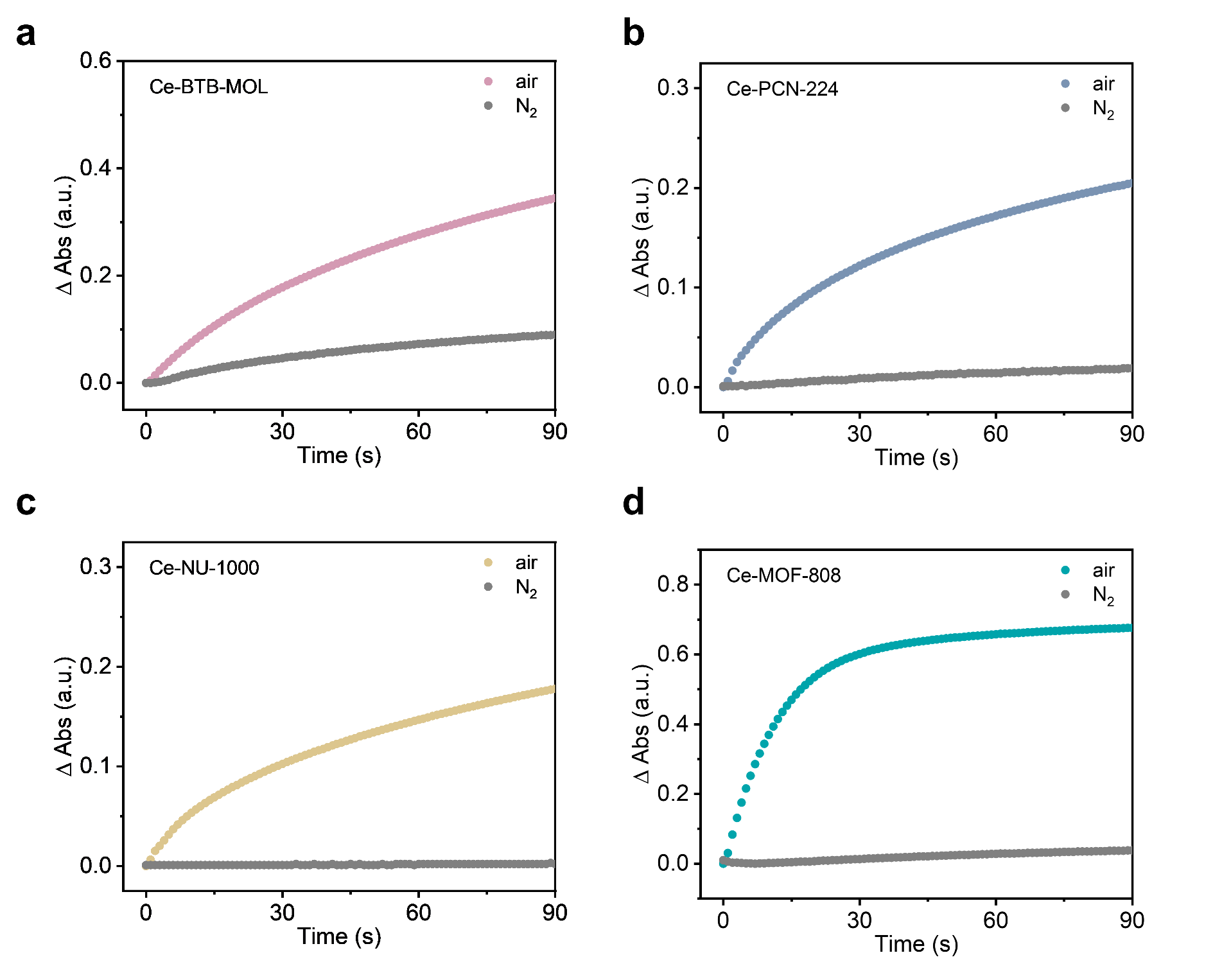


**Figure S29**. The oxidase-like catalytic kinetics curves of Ce-BTB-MOL (a), Ce-PCN-224 (b), Ce-NU-1000 (c) and Ce-MOF-808 (d) under the air and N_2_ atmospheres.

**Note:** Under N_2_ atmosphere, we found that the catalytic activities of these Ce-MOFs significantly decreased, which was similar to the observation in MD-Ce-UiO-66. These indicating that O_2_ was the electron acceptor in all the reactions.


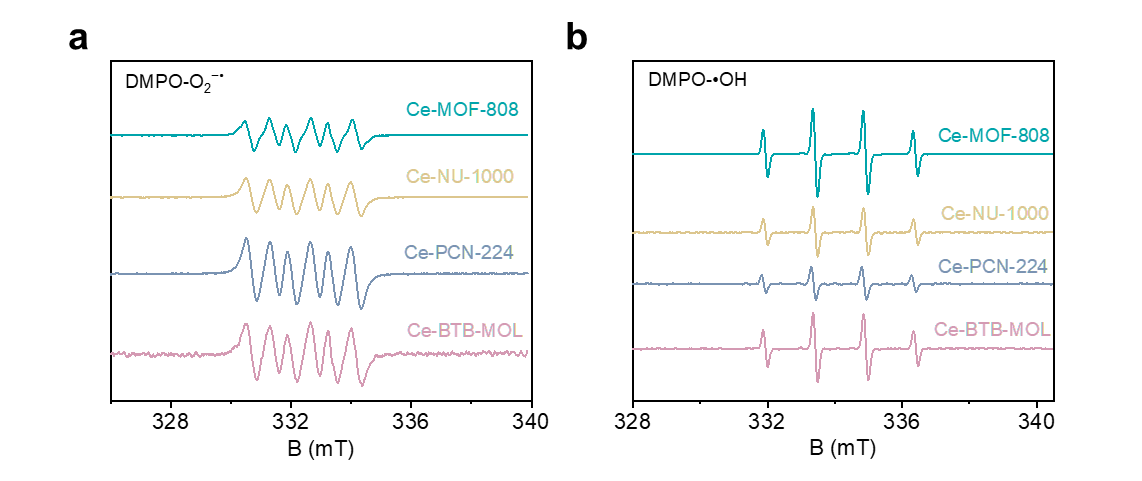


**Figure S30**. The EPR spectra of DMPO-O_2_^−•^ (a) and DMPO-•OH (b) for Ce-BTB-MOL, Ce-PCN-224, Ce-NU-1000 and Ce-MOF-808.


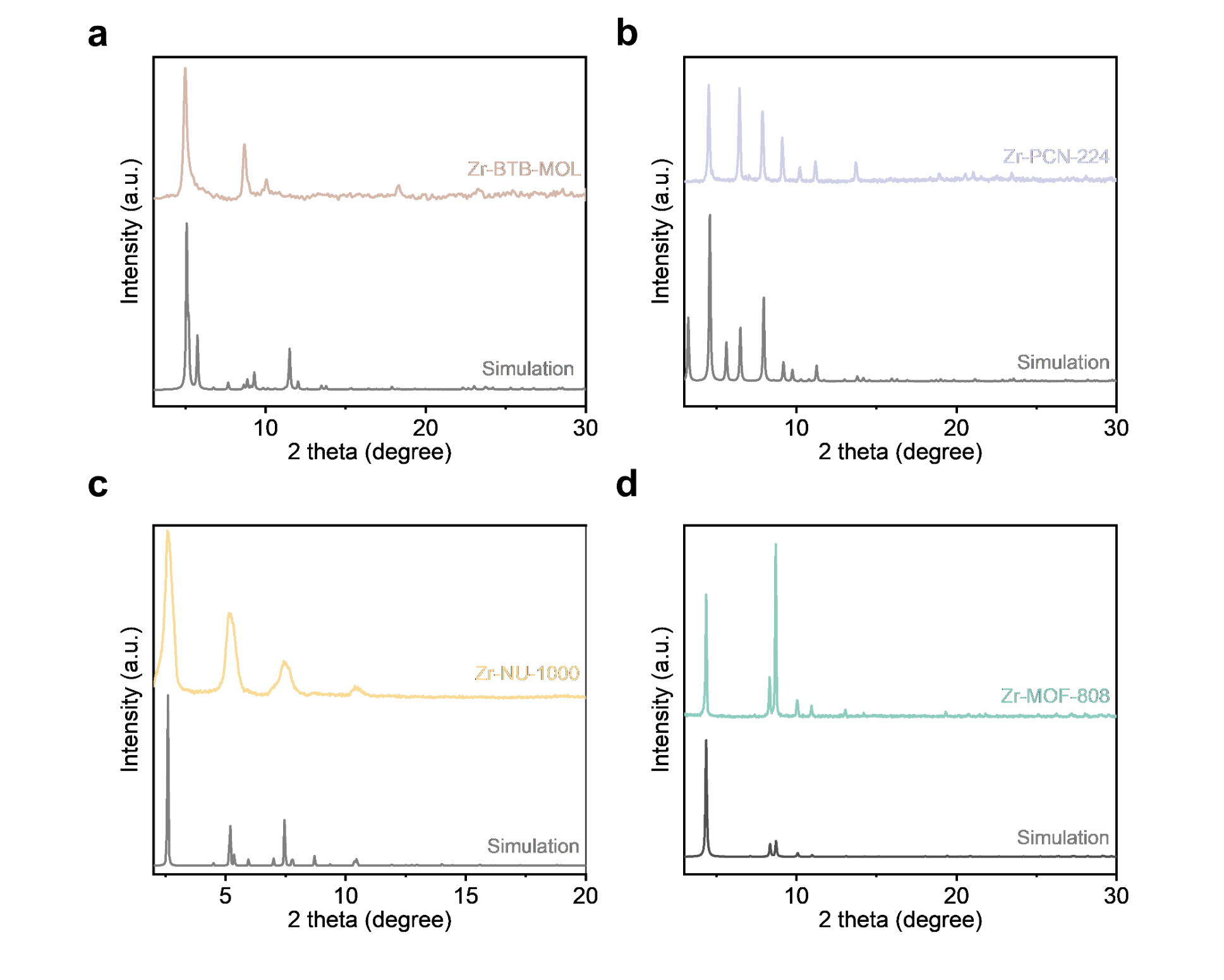


**Figure S31**. The PXRD patterns of Zr-BTB-MOL (a), Zr-PCN-224 (b), Zr-NU-1000 (c) and Zr-MOF-808 (d). All of them showed strong Bragg diffraction peaks, matching well with the corresponding simulated structures.


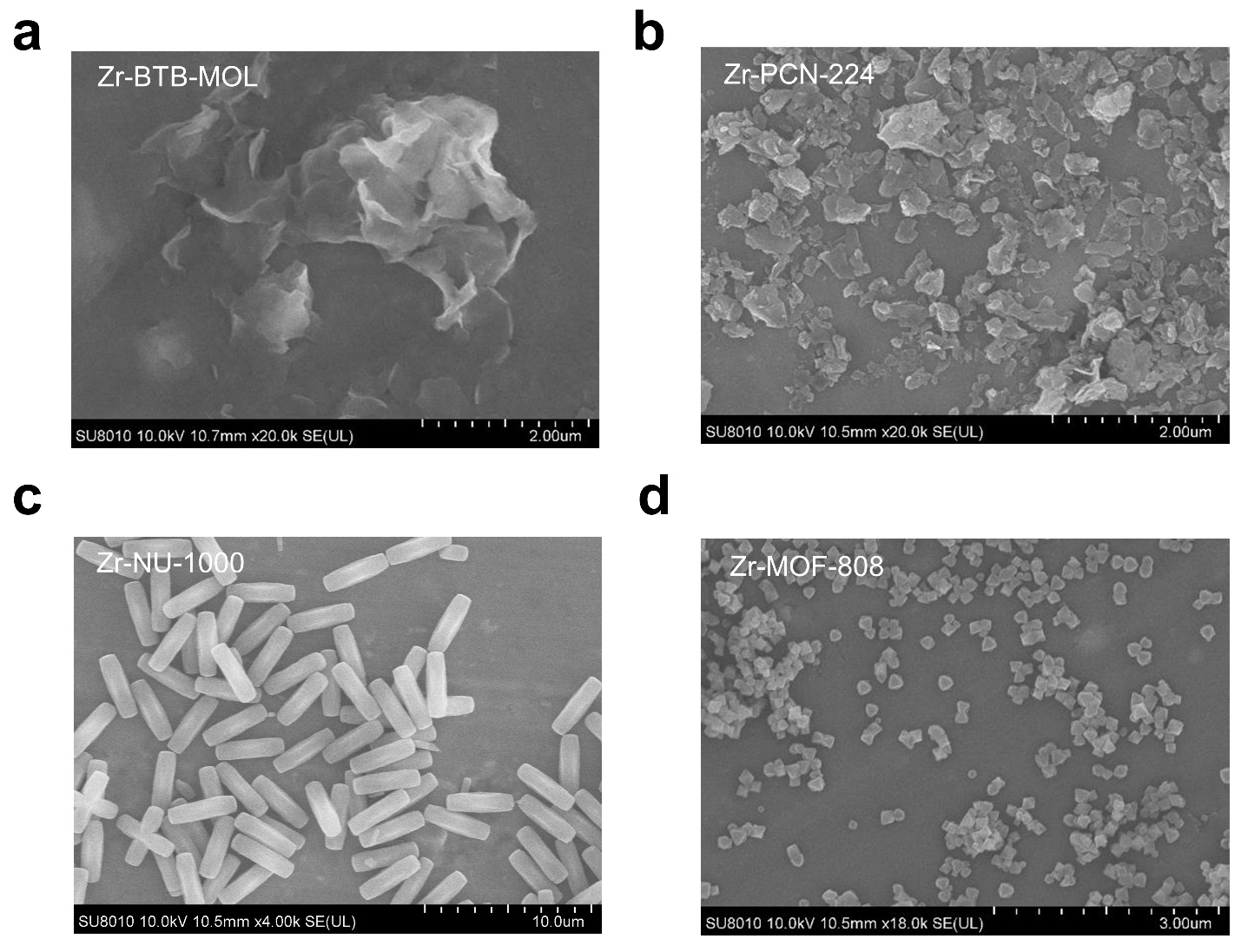


**Figure S32**. The SEM images of Zr-BTB-MOL (a), Zr-PCN-224 (b), Zr-NU-1000 (c) and Zr-MOF-808 (d).


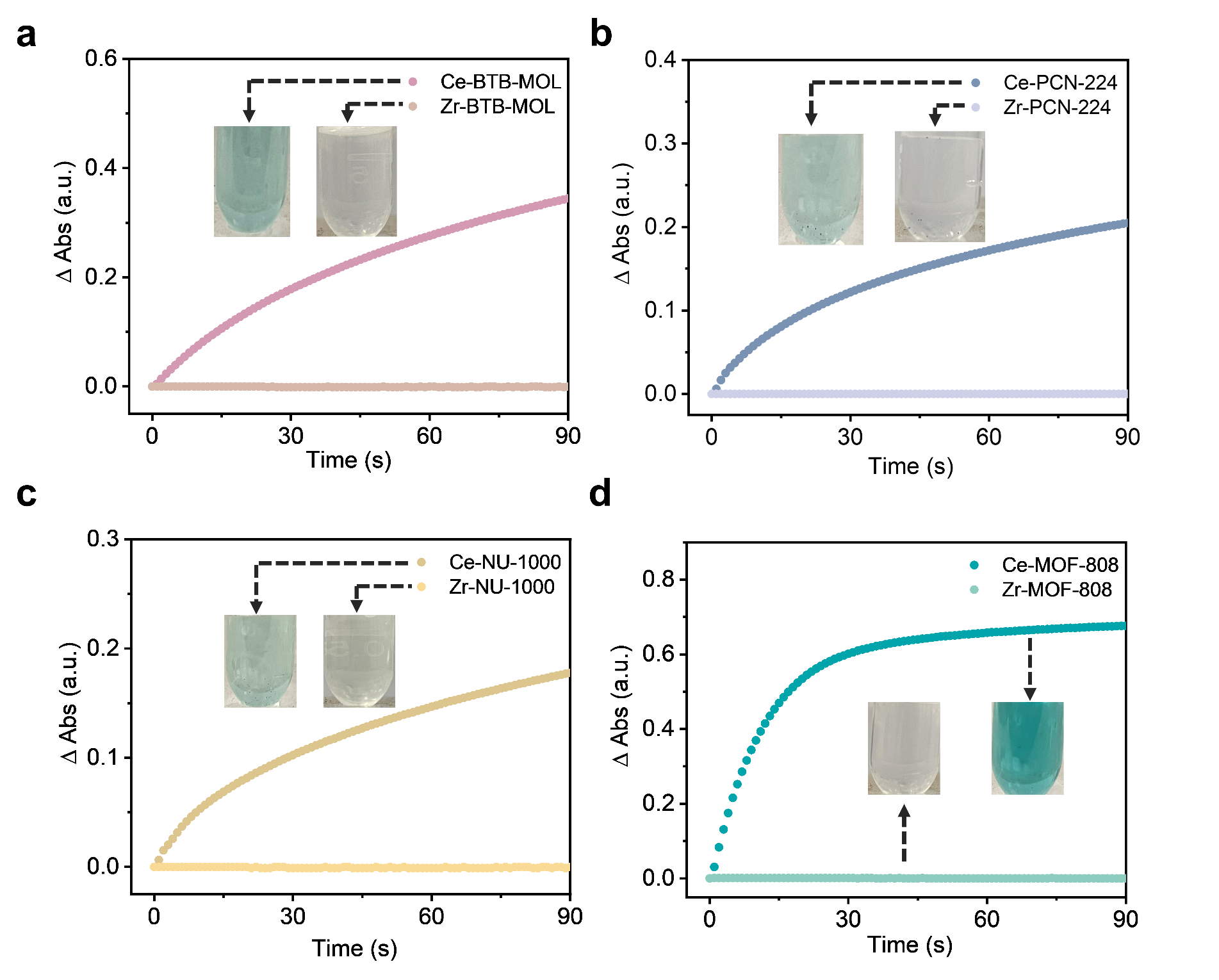


**Figure S33**. The oxidase-like catalytic kinetics curves: (a) Zr-BTB-MOL and Ce-BTB-MOL; (b) Zr-PCN-224 and Ce-PCN-224; (c) Zr-NU-1000 and Ce-NU-1000; (d) Zr-MOF-808 and Ce-MOF-808. Insets are the photographs recording the apparent color changes after 2 min reaction. No oxidase-like activity was found in all isostructural Zr-MOFs, manifesting that the catalytic activity was originated from the Ce_6_ clusters.


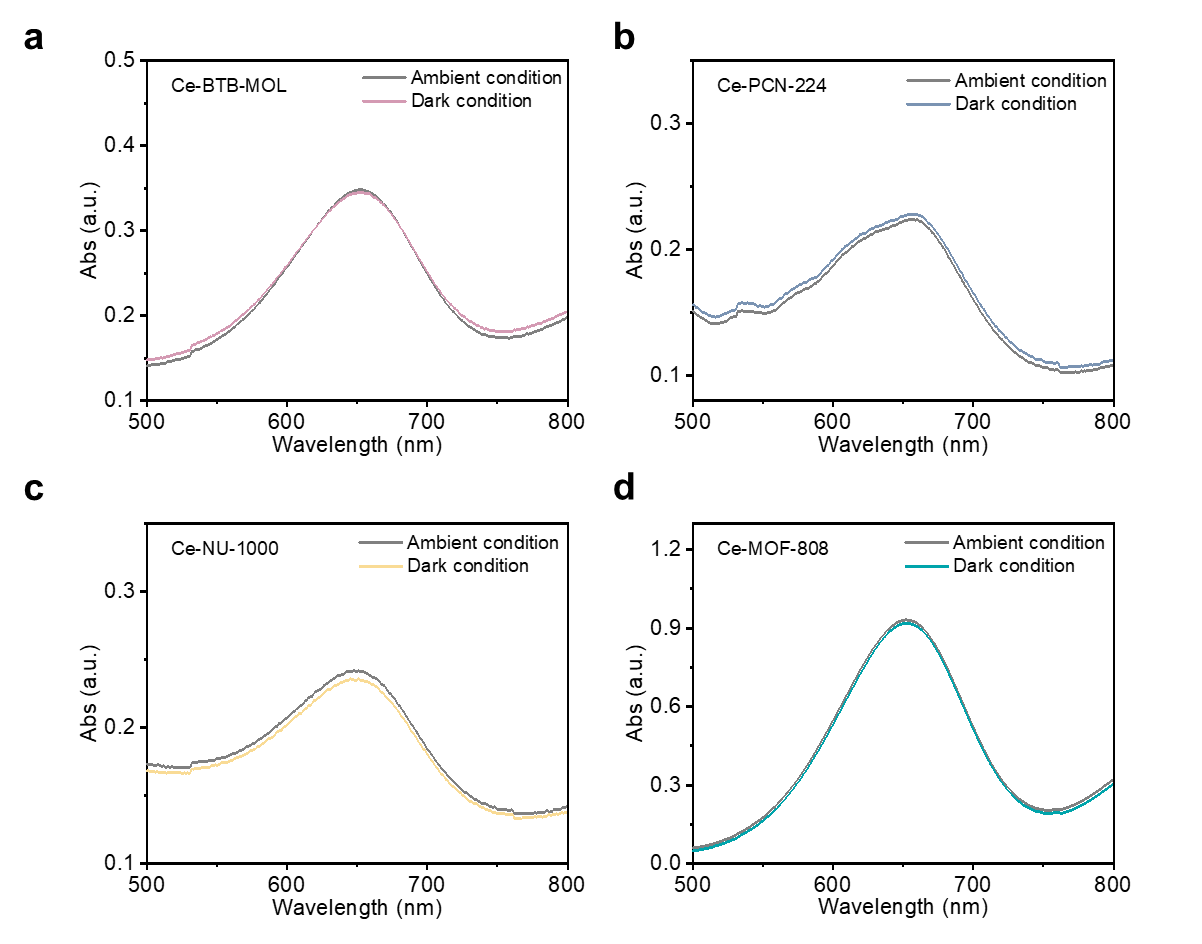


**Figure S34**. Oxidase-like activity of Ce-BTB-MOL (a), Ce-PCN-224 (b), Ce-NU-1000 (c) and Ce-MOF-808 (d) under ambient condition or dark condition. The UV-vis spectra presented the absorbance of the catalytic product after 2 min reaction. Obviously, the catalytic activities of these Ce-MOFs were not affected by light irradiation.


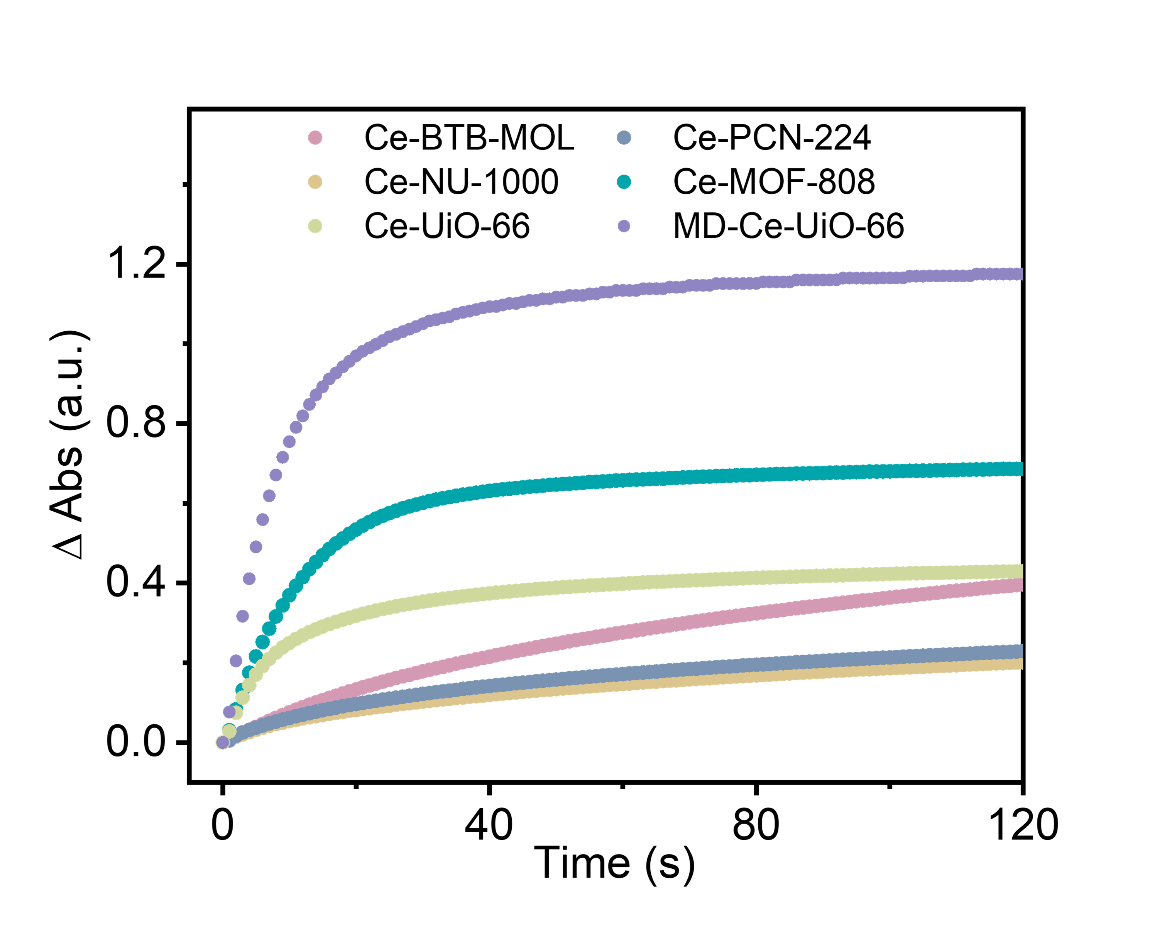


**Figure S35**. The oxidase-like catalytic kinetics curves of Ce-BTB-MOL, Ce-PCN-224, Ce-NU-1000, Ce-MOF-808, Ce-UiO-66 and MD-Ce-UiO-66 under same Ce amount. The Ce concentration in each trial was controlled to be 15 μg/mL.


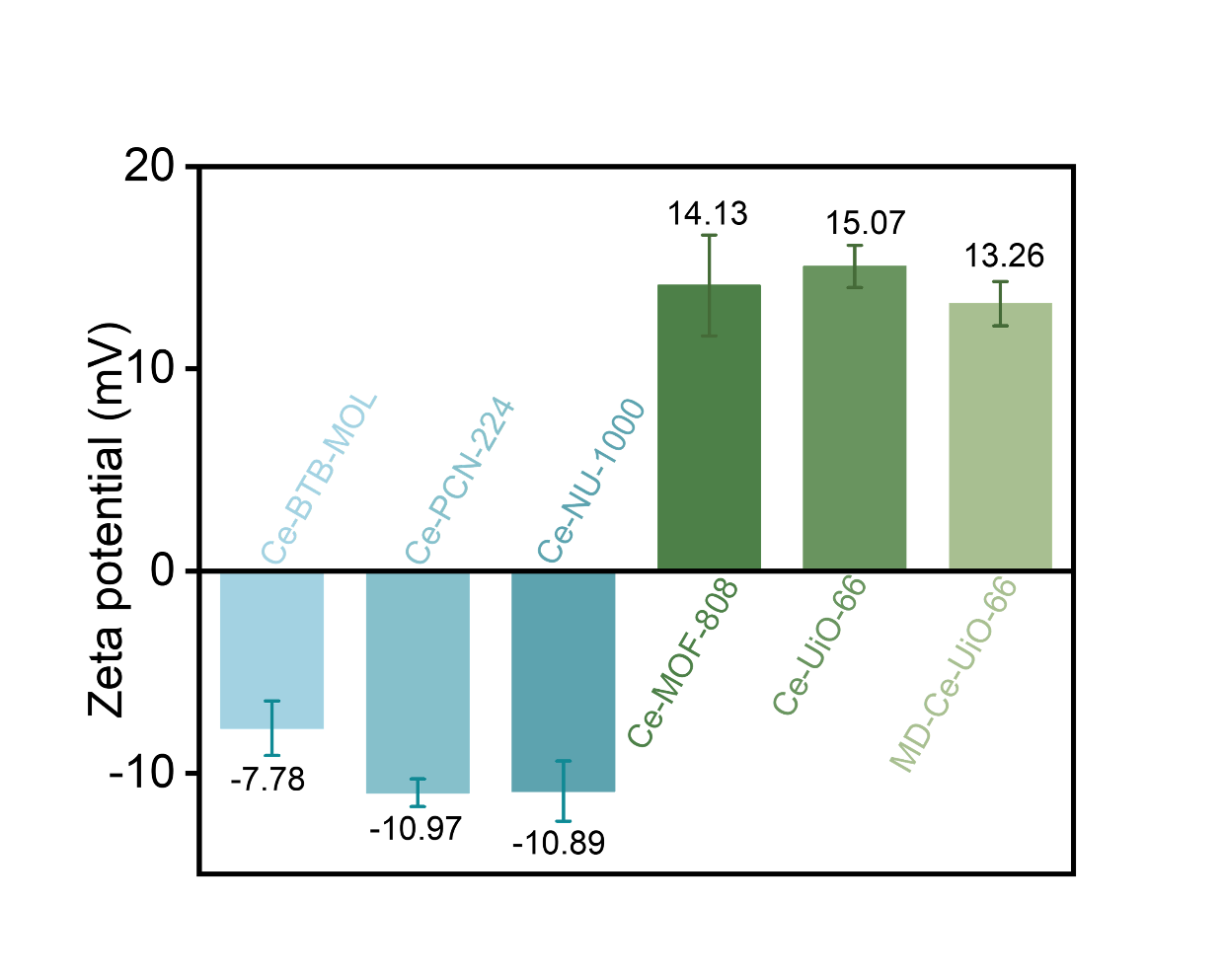


**Figure S36**. The zeta potentials of different Ce-MOF nanoagents.


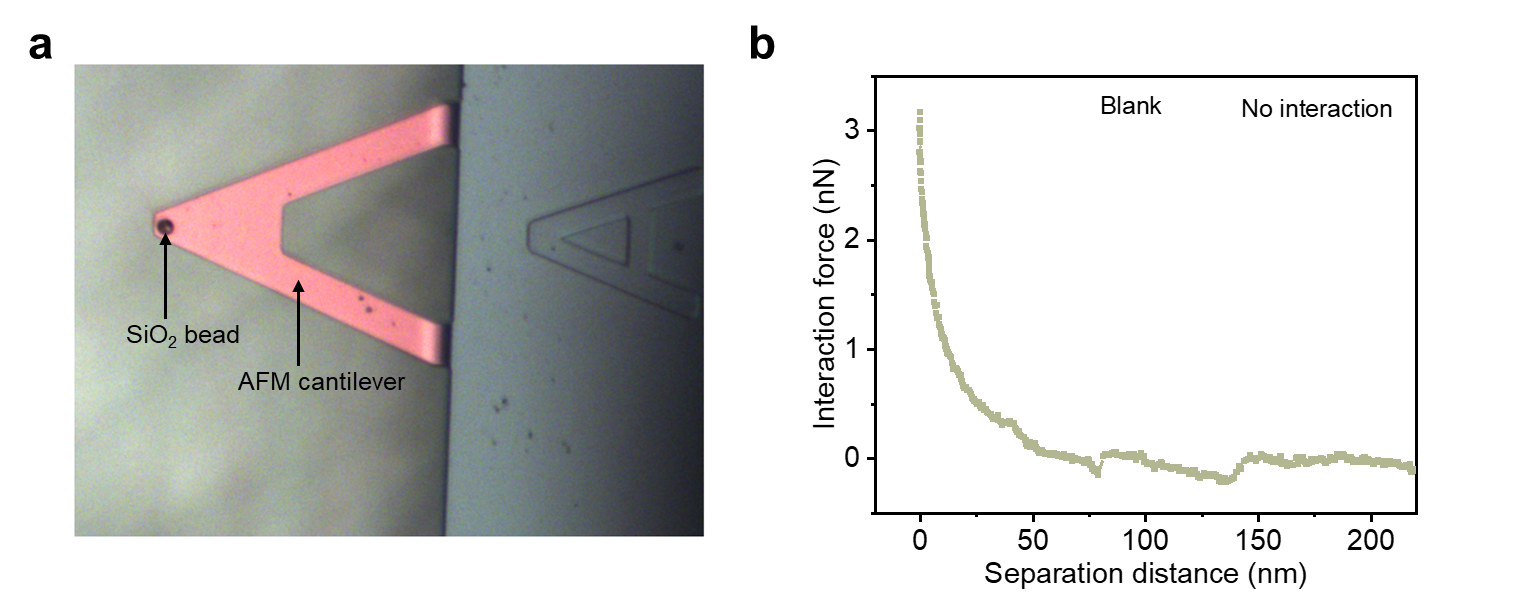


**Figure S37**. (a) The optical image of SiO_2_ bead mounted on the AFM cantilever. (b) AFM force-distance curve between *E. coli* and smooth glass sheet, showing no interaction.


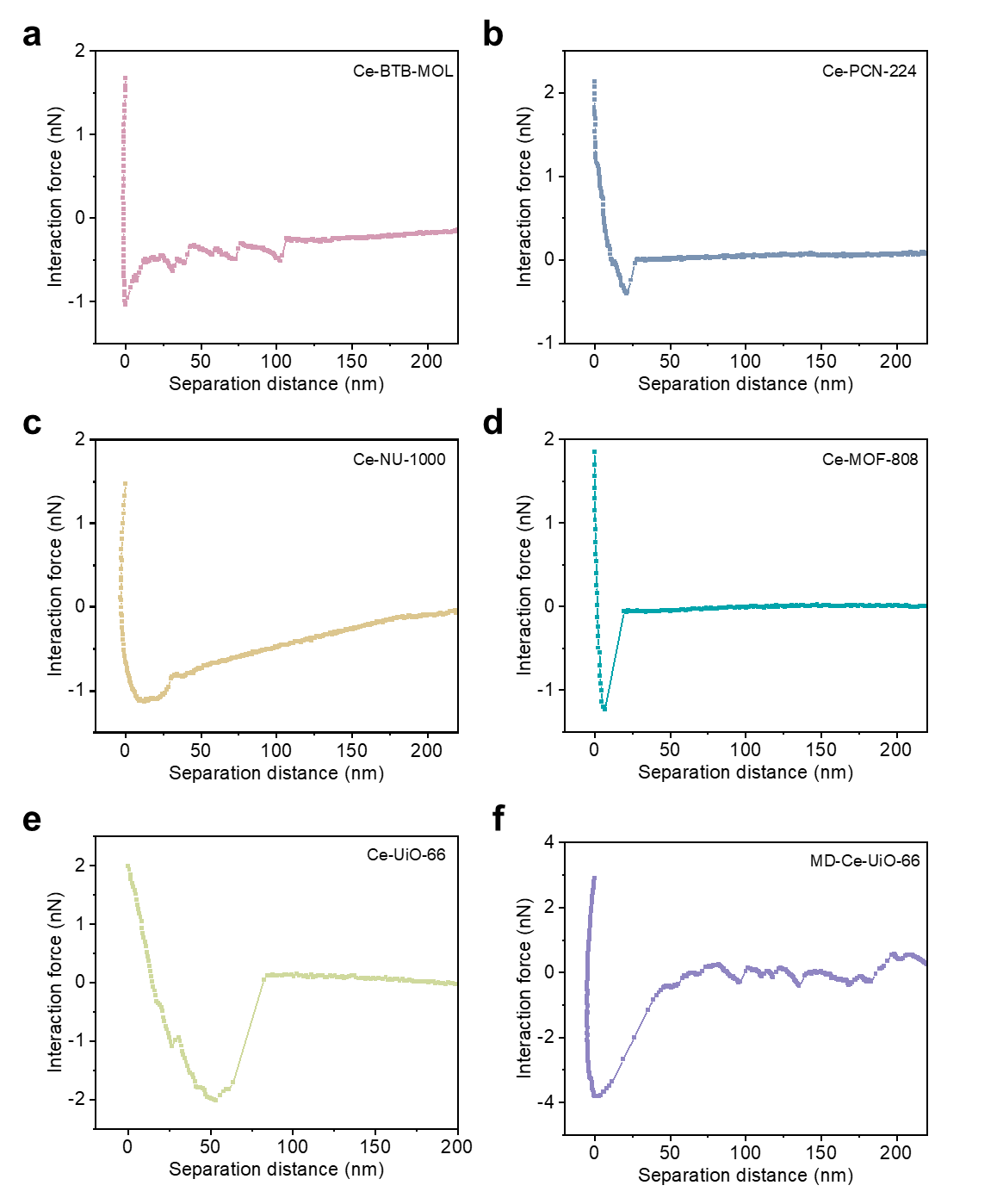


**Figure S38**. The AFM force-distance curves between *E. coli* and different Ce-MOFs: (a) Ce-BTB-MOL; (b) Ce-PCN-224; (c) Ce-NU-1000; (d) Ce-MOF-808; (e) Ce-UiO-66; (f) MD-Ce-UiO-66.


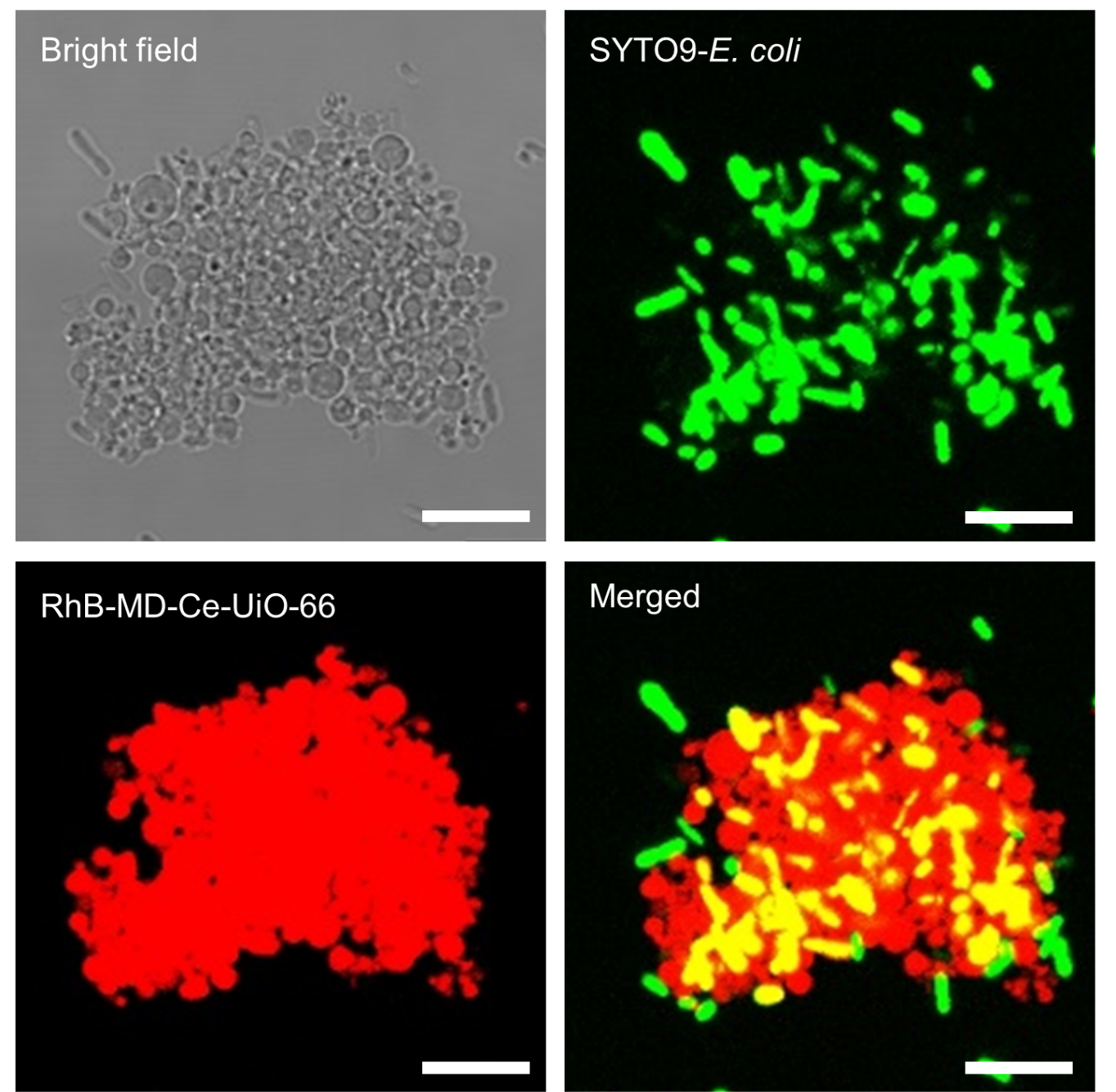


**Figure S39**. CLSM images showing the spatial distribution of SYTO9-*E. coli* and RhB-MD-Ce-UiO-66. Scale bar: 10 μm.


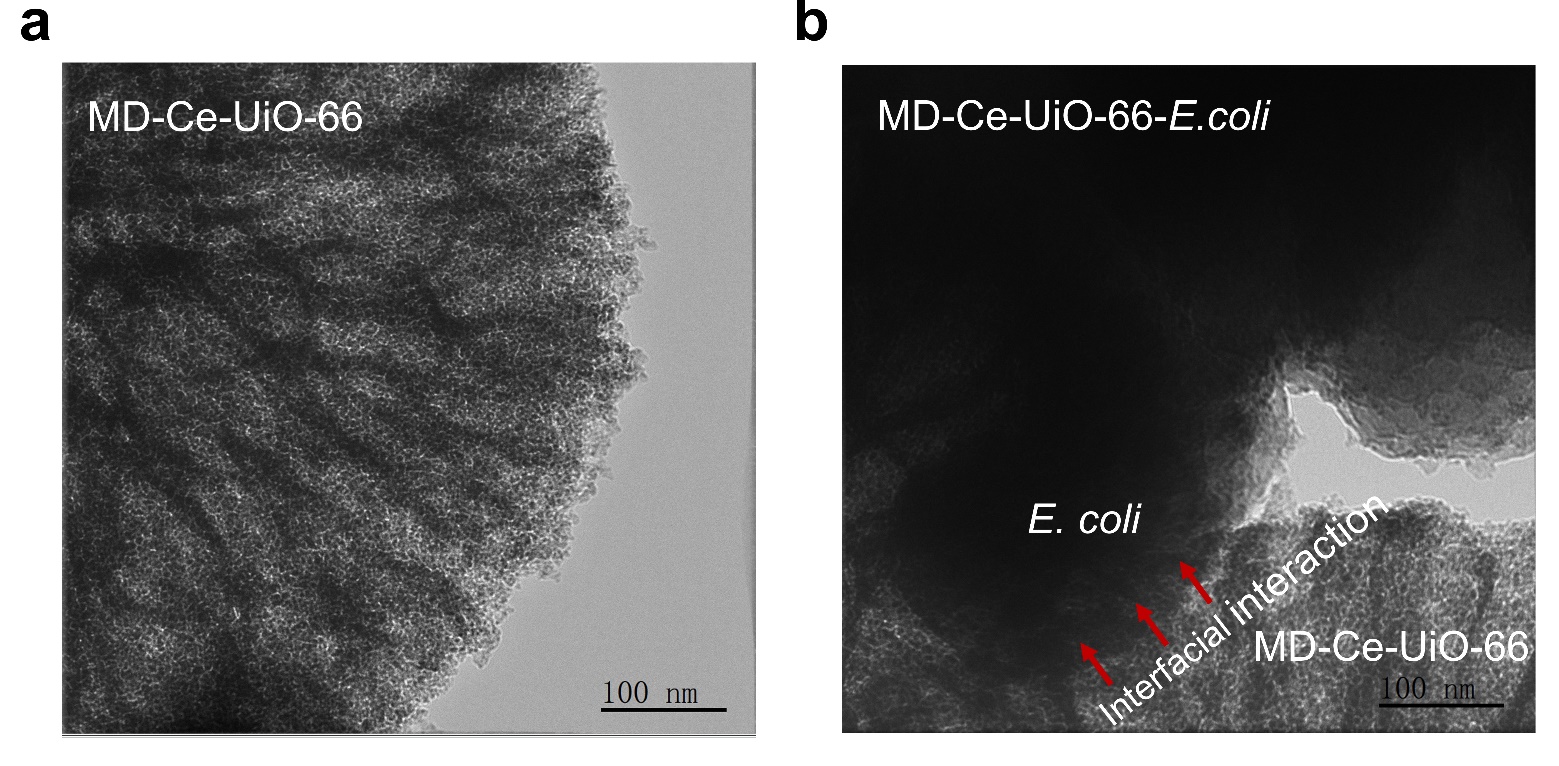


**Figure S40**. Cryo-EM images of the surface of MD-Ce-UiO-66 (a) and the interfacial interactions between *E. coli*. and MD-Ce-UiO-66 (b).


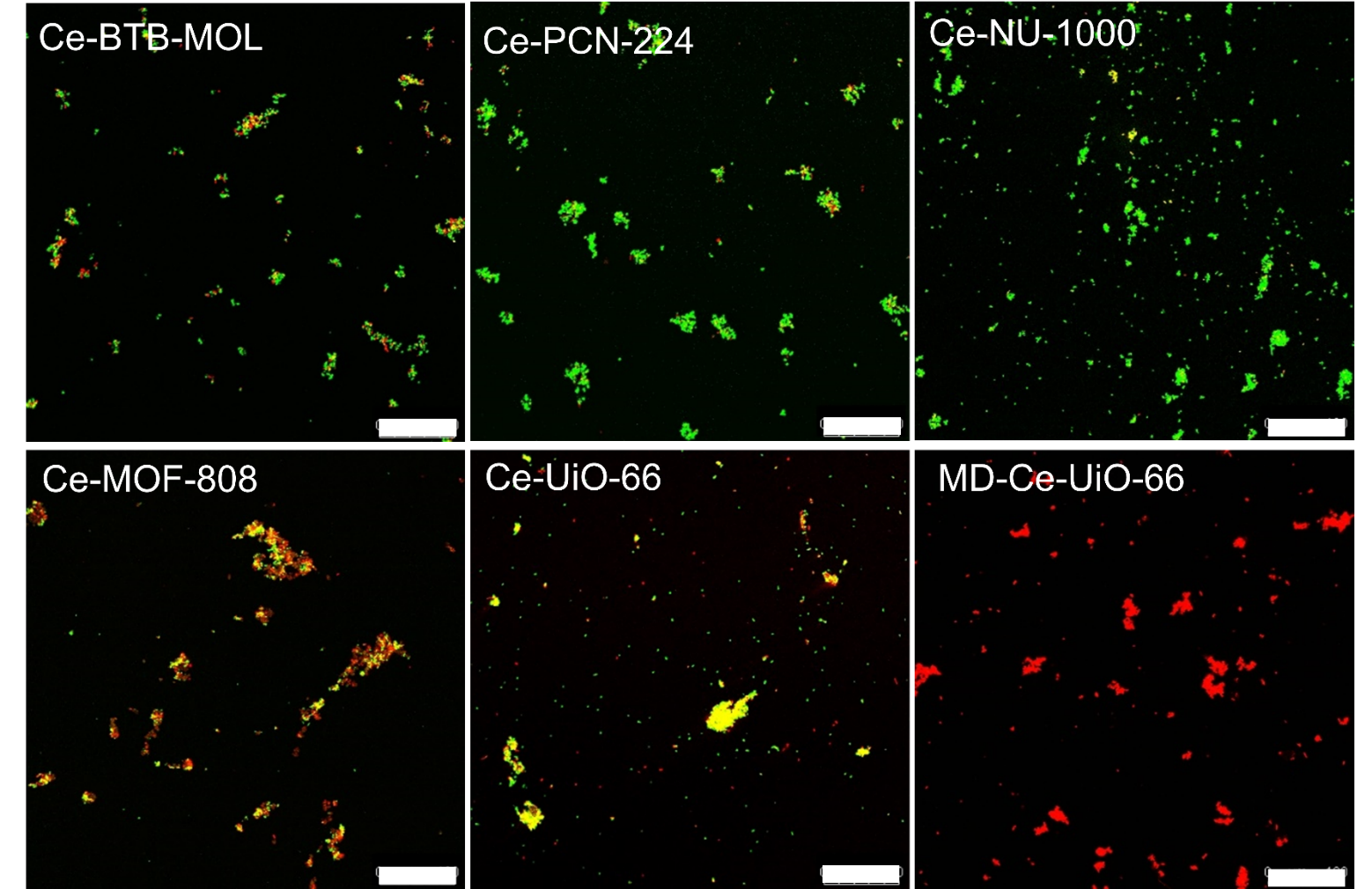


**Figure S41**. SYTO9/PI two-color fluorescent images for the live (green fluorescence) and dead (red fluorescence) bacterial staining assay of different Ce-MOFs. Scale bar: 10 μm.


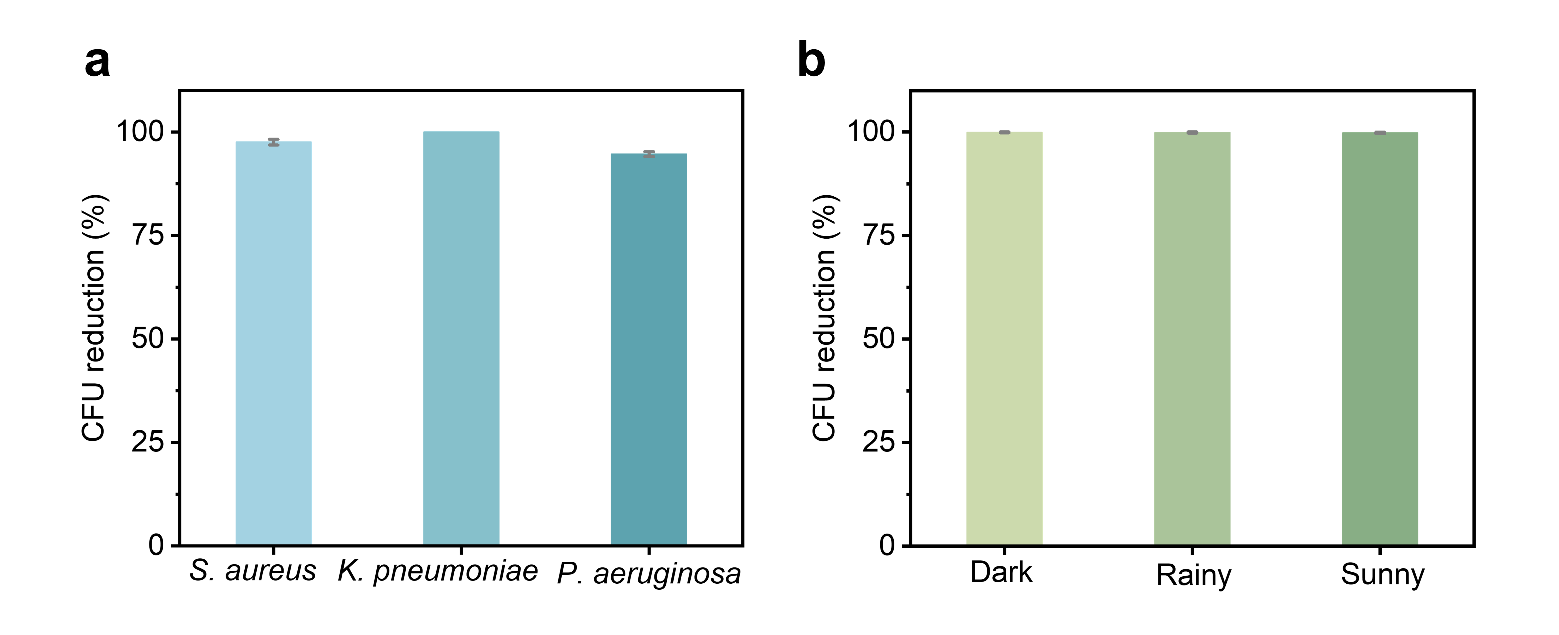


**Figure S42**. (a) The bacterial viabilities of *S. aureus*, *K. pneumoniae* and *P. aeruginosa* after treatment with MD-Ce-UiO-66. (b) The antibacterial performances of MD-Ce-UiO-66 for *E. coli* in the dark and on a rainy and a sunny day.


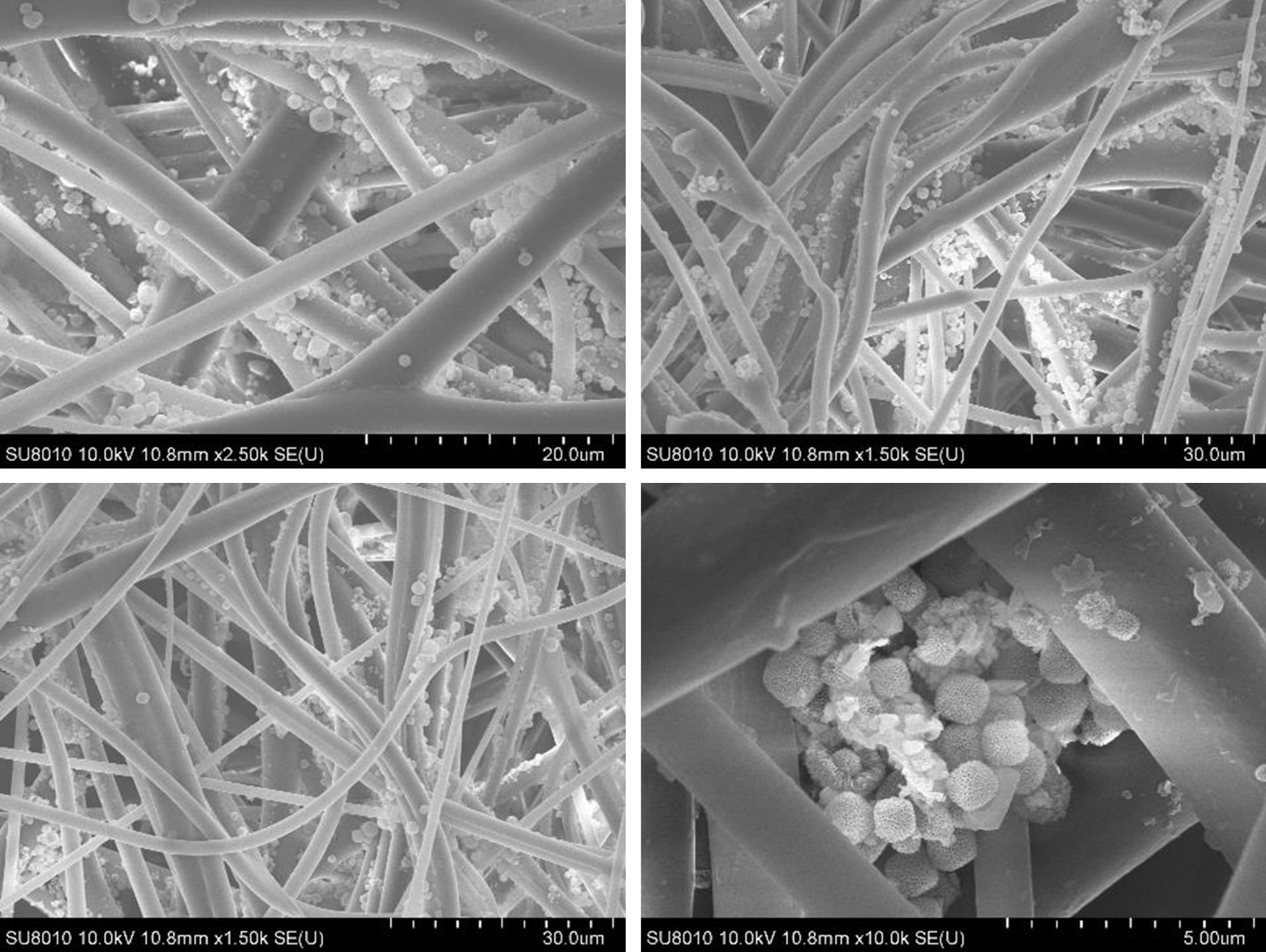


**Figure S43**. SEM images of the MD-Ce-UiO-66-loaded on fibrous membrane, which was used as the second layer for face mask.


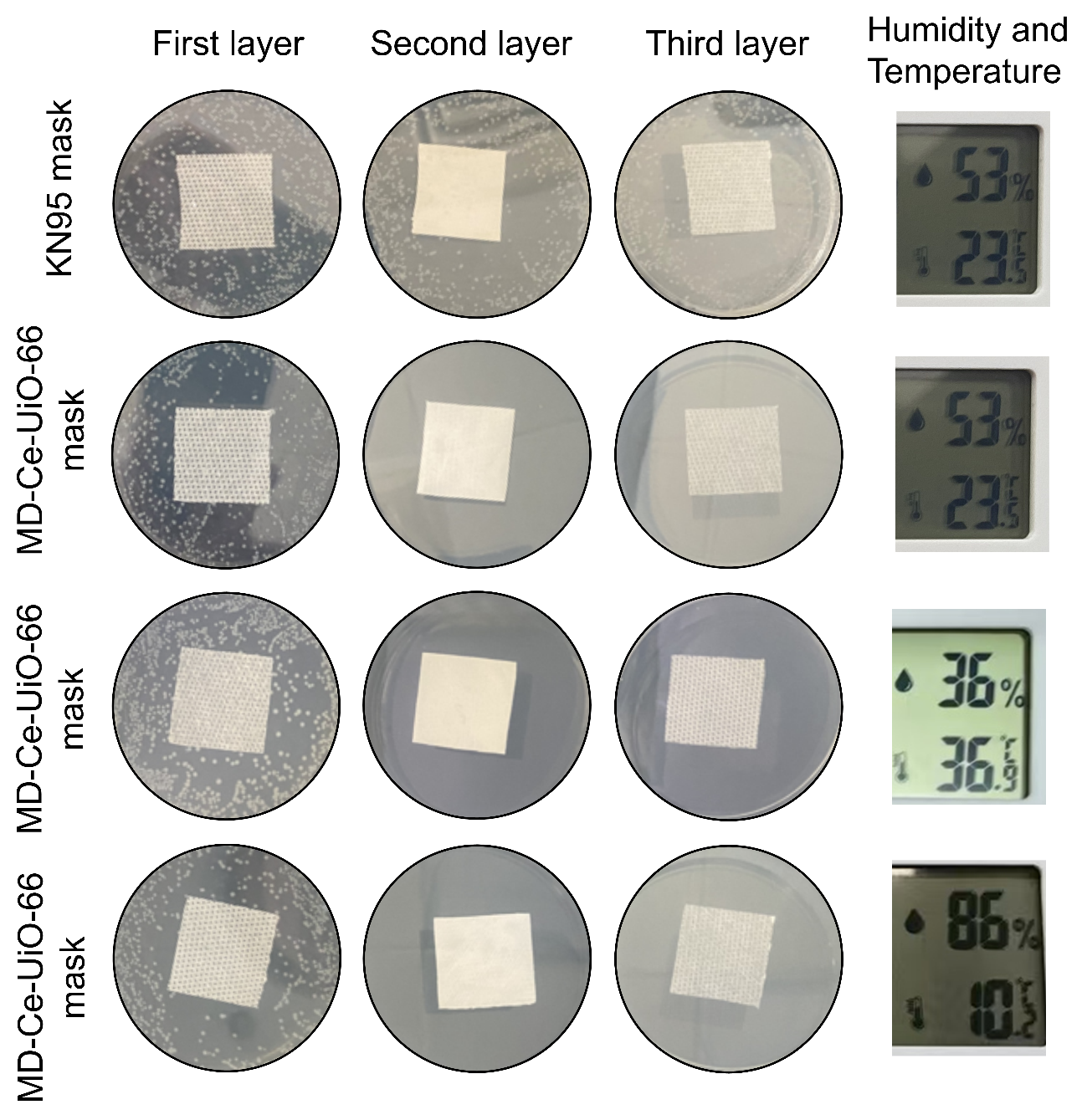


**Figure S44**. *E. coli* residual on the first, second, and third layers of MD-Ce-UiO-66 mask and commercially available KN95 mask under different humidity and temperature conditions.


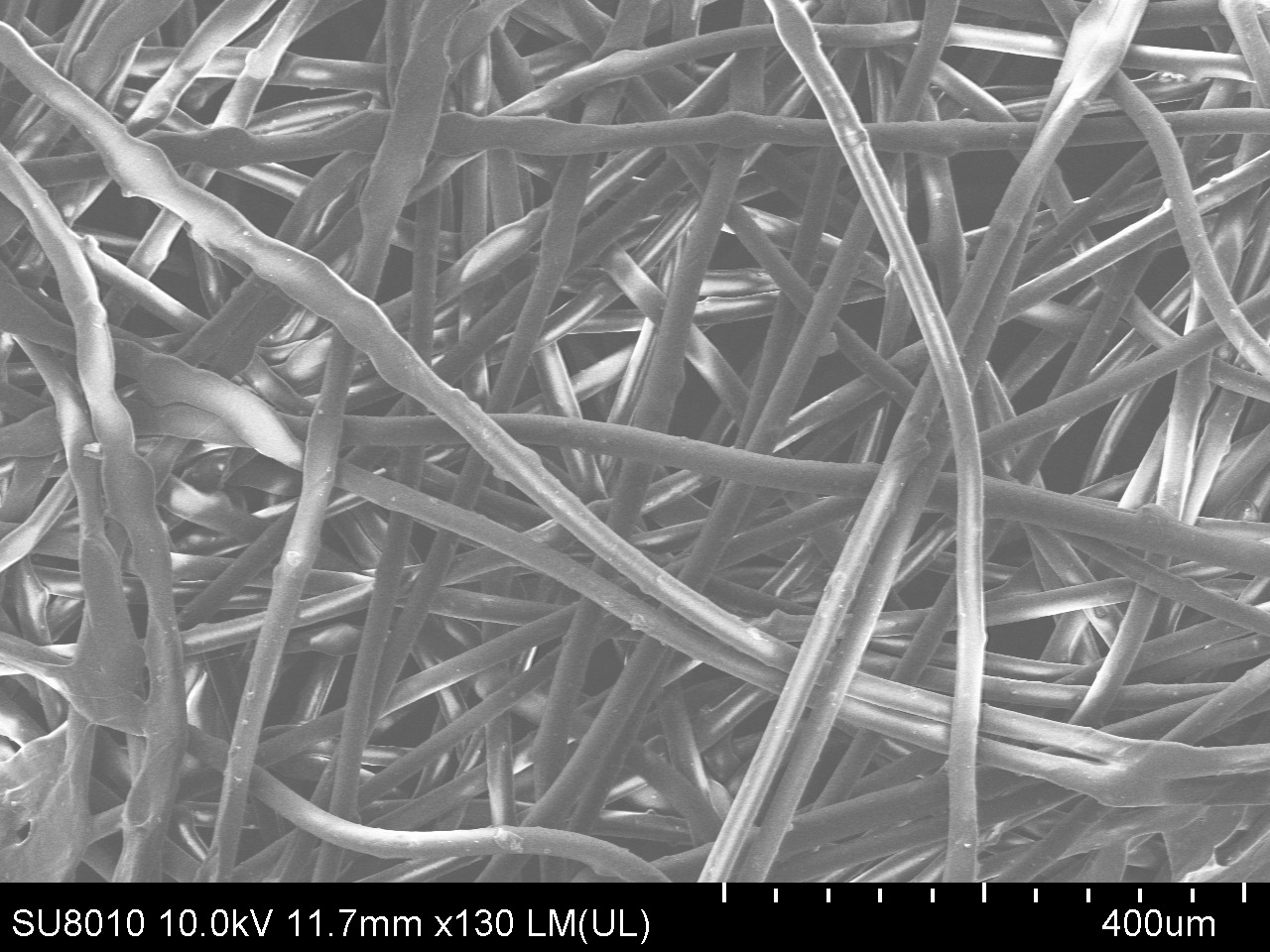


**Figure S45**. The SEM image of the third layer of MD-Ce-UiO-66 mask, which undergone a simulated respiratory droplet propagation experiment.

**References**

[1] M. Lammert, M. T. Wharmby, S. Smolders, B. Bueken, A. Lieb, K. A. Lomachenko, D. D. Vos, N. Stock, *Chem. Commun.* **2015**, *51*, 12578-12581.

[2] J. Yang, K. Li, J. Gu, *ACS Mater. Lett.* **2022**, *4*, 385-391.

[3] Y. Song, Y. Pi, X. Feng, K. Ni, Z. Xu, J. S. Chen, Z. Li, W. Lin, *J. Am. Chem. Soc.* **2020**, *142*, 6866-6871.

[4] S. L. Estes, M. R. Antonio, L. Soderholm, *J. Phys. Chem. C.* **2016**, *120*, 5810-5818.

[5] S. Smolders, A. Struyf, H. Reinsch, B. Bueken, T. Rhauderwiek, L. Mintrop, P. Kurz, N. Stock, D. E. De Vos, *Chem. Commun.* **2018**, *54*, 876-879.

[6] M. Lammert, C. Glißmann, H. Reinsch, N. Stock, *Cryst. Growth Des.* **2017**, *17*, 1125-1131.

[7] L.-L. Ling, W. Yang, P. Yan, M. Wang, H.-L. Jiang, *Angew. Chem. Int. Ed.* **2022**, *61*, e202116396.

[8] D. Feng, W.-C. Chung, Z. Wei, Z.-Y. Gu, H.-L. Jiang, Y.-P. Chen, D. J. Darensbourg, H.-C. Zhou, *J. Am. Chem. Soc.* **2013**, *135*, 17105-17110.

[9] P. Li, R. C. Klet, S.-Y. Moon, T. C. Wang, P. Deria, A. W. Peters, B. M. Klahr, H.-J. Park, S. S. Al-Juaid, J. T. Hupp, O. K. Farha, *Chem. Commun.* **2015**, *51*, 10925-10928.

[10] A. Sharma, J. Lim, S. Jeong, S. Won, J. Seong, S. Lee, Y. S. Kim, S. B. Baek, M. S. Lah, *Angew. Chem. Int. Ed.* **2021**, *60*, 14334-14338.

[11] G. C. Shearer, S. Chavan, S. Bordiga, S. Svelle, U. Olsbye, K. P. Lillerud, *Chem. Mater.* **2016**, *28*, 3749-3761.

[12] L. Valenzano, B. Civalleri, S. Chavan, S. Bordiga, M. H. Nilsen, S. Jakobsen, K. P. Lillerud, C. Lamberti, *Chem. Mater.* **2011**, *23*, 1700-1718.

[13] G. Kresse, J. Furthmüller, *Comp. Mater. Sci.* **1996**, *6*, 15-50.

[14] G. Kresse, J. Furthmüller, *Phy. Rev. B* **1996**, *54*, 11169-11186.

[15] J. P. Perdew, K. Burke, M. Ernzerhof, *Phys. Rev. Lett.* **1996**, *77*, 3865-3868.

[16] G. Kresse, D. Joubert, *Phy. Rev. B* **1999**, *59*, 1758-1775.

[17] P. E. Blöchl, *Phy. Rev. B* **1994**, *50*, 17953-17979.

[18] S. Grimme, J. Antony, S. Ehrlich, H. Krieg, *J. Chem. Phys.* **2010**, *132*, 154104.

[19] X. Jing, Y. Wu, D. Wang, C. Qu, J. Liu, C. Gao, A. Mohamed, Q. Huang, P. Cai, N. M. Ashry, *Environ. Sci. Technol.* **2022**, *56*, 16707-16715.

[20] X. Li, B. E. Logan, *Langmuir* **2004**, *20*, 8817-8822.

[21] Q. Huang, H. Wu, P. Cai, J. B. Fein, W. Chen, *Sci. Rep.* **2015**, *5*, 16857.
